# Respirable Metals, Bacteria, and Fungi during a Saharan–Sahelian Dust Event in Houston, Texas

**DOI:** 10.1021/acs.est.3c04158

**Published:** 2023-11-09

**Authors:** Sourav Das, Alyvia McEwen, Joseph Prospero, Daniel Spalink, Shankararaman Chellam

**Affiliations:** † Department of Civil & Environmental Engineering, Texas A&M University, College Station, Texas 77843, United States; ‡ Rosenstiel School of Marine and Atmospheric Science, University of Miami, Miami, Florida 33149, United States; § Department of Ecology and Conservation Biology, Texas A&M University, College Station, Texas 77843, United States; ∥ Department of Chemical Engineering, Texas A&M University, College Station, Texas 77843, United States

**Keywords:** bioaerosols, aerobiology, airborne metals, mycobiome, bacteriome, urban aerosols, next-generation sequencing

## Abstract

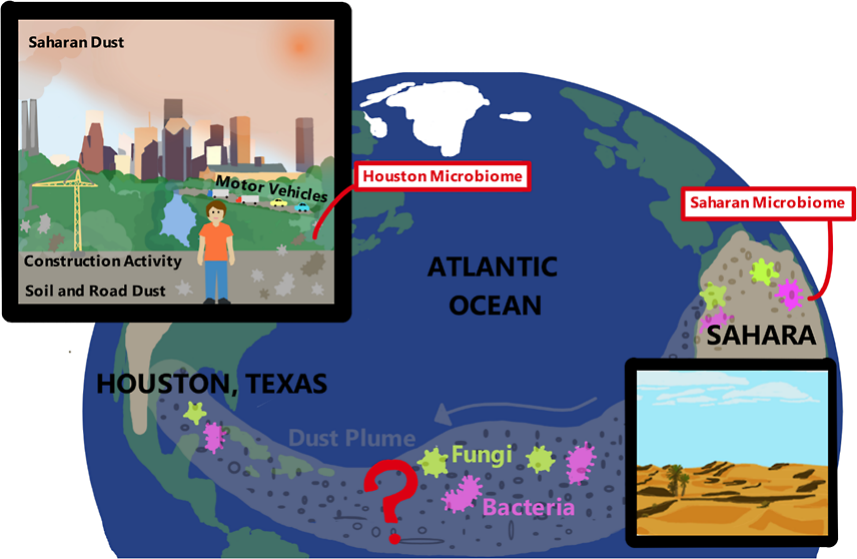

Although airborne bacteria and fungi can impact human, animal, plant, and ecosystem health, very few studies have investigated the possible impact of their long-range transport in the context of more commonly measured aerosol species, especially those present in an urban environment. We report first-of-kind simultaneous measurements of the elemental and microbial composition of North American respirable airborne particulate matter concurrent with a Saharan–Sahelian dust episode. Comprehensive taxonomic and phylogenetic profiles of microbial communities obtained by 16S/18S/ITS rDNA sequencing identified hundreds of bacteria and fungi, including several cataloged in the World Health Organization’s lists of global priority human pathogens along with numerous other animal and plant pathogens and (poly)extremophiles. While elemental analysis sensitively tracked long-range transported Saharan dust and its mixing with locally emitted aerosols, microbial diversity, phylogeny, composition, and abundance did not well correlate with the apportioned African dust mass. Bacterial/fungal diversity, phylogenetic signal, and community turnover were strongly correlated to apportioned sources (especially vehicular emissions and construction activities) and elemental composition (especially calcium). Bacterial communities were substantially more dissimilar from each other across sampling days than were fungal communities. Generalized dissimilarity modeling revealed that daily compositional turnover in both communities was linked to calcium concentrations and aerosols from local vehicles and Saharan dust. Because African dust is known to impact large areas in northern South America, the Caribbean Basin, and the southern United States, the microbiological impacts of such long-range transport should be assessed in these regions.

## Abstract 2

Ambient aerosols in South Texas during an intercontinentally transported African dust episode contained microbiota (bacteria and fungi) that impact human, animal, plant, and ecosystem health and were diluted in anthropogenic metals.

## Introduction

Because aerosols impact human and ecosystem health,^1−9^ much research has focused on characterizing their chemical composition and epidemiological/toxicological effects to develop scientific policy enabling targeted emission control strategies to reduce their mass concentrations.^8,10,11^ In contrast, comparatively fewer reports have considered airborne microorganisms even though they also influence public health and the environment.^1,12−14^

Ambient aerosols in population centers across the globe are a mixture of local emissions and long-range transported particles, including desert dust.^2,3,9,15−27^ Houston, Texas, is no exception with its air quality being dominated by local anthropogenic emissions^28,29^ and periodically impacted by aeolian dust from North Africa, principally the Sahara-Sahel region.^20−22^ Greater Houston has a population of more than 7 million (the 5^th^ largest in the United States) and faces scrutiny about its air quality and environmental justice.^16,28,30^ Consequently, the physical–chemical characteristics of particulate matter (PM) in this region have been studied in detail, but like in most other places, the aerosols’ biological composition is relatively unknown.^10,11,16−18,21,22^

Windblown dust from North Africa^20,25^ adversely impacts human health^2,9,19,23−26,31^ and disperses microorganisms to distal locations,^2,9,25,26,32−34^ which to date has been largely delineated in places relatively proximal to the source including Africa, the Mediterranean Sea, the Middle East, and southern Europe.^2,9,23,24,26,27,35−44^ Although few investigations have identified microorganisms associated with African dust in the Caribbean, Middle East, and remote portions of the Atlantic Ocean,^27,32,38,40,41,45−47^ none have considered population centers in the North American mainland. Furthermore, the available African dust microbiological investigations have focused on bacteria,^35−38,40,42,48−51^ and only a few reports of fungi exist.^34,41,52,53^ Fungi are also important to enumerate because they are subject to long-distance dispersal^34,53^ and little attention has been paid to them even though invasive fungal diseases and antifungal resistance are rising across the globe.^54^ Additionally, a recent study has recommended fungal measurements to adequately model their global-scale transport.^34^

To fill in the previously identified knowledge gaps, we initiated a first-of-its-kind study in a North American metroplex to comprehensively characterize the elemental composition, bacteriome, and mycobiome in respirable particulate matter (PM_10_) that included samples collected during a Saharan-Sahelian dust event. We focused on PM_10_ to capture both bacteria and fungi because most fungi are larger than 2.5 μm, and although individual bacteria are smaller than 2 μm, they exist generally as larger aggregates in aerosols.^42^ This research was driven by two hypotheses: (i) peak African dust intrusion would modify the microbial community in ambient aerosols and (ii) the abundance of specific bacteria and fungi would be correlated to concentrations of individual elements. Specific objectives were to (i) measure ∼50 major and trace elements including the main group and (inner)transition metals to perform source apportionment,^33^ (ii) amplify the V3 and V4 hypervariable regions of 16S rDNA and the highly variable internal transcribed spacer (ITS) region of rDNA (18S/ITS) and use bioinformatics to identify the composition and abundance of prokaryotes and eukaryotes, respectively,^33^ and (iii) obtain empirical clues to interrelationships between elemental concentrations and source contribution estimates with microbial diversity within our data set. Our principal motivation was to characterize the elemental and microbiological composition of urban aerosols in unison for human, animal, plant, and ecosystem health purposes.

## Materials and Methods

Most procedural details are provided in the Supporting Information to comply with journal length restrictions.

### Aerosol Sampling, Elemental Analysis, Saharan Dust Identification, and Source Apportionment

Nine daily PM_10_ samples (labeled S1–S9) were collected on polytetrafluoroethylene (PTFE) filters (Table 1) in Houston, Texas (29.73372–95.25759). Information on sampling, elemental analysis, Saharan dust identification, and source apportionment is in Sections S1–S7.

**Table 1:** Sampling Summary, Aerosol Mass Concentrations, Initial Categorization of African Dust Influence during One Episode in the Year 2018^a^^,^^c^

sample ID	start date	end date	PM_10_ (μg/m^3^)	PM_2.5_^b^ (μg/m^3^)	PM_10_/PM_2.5_ mass ratio	apportioned Saharan dust contribution (μg/m^3^)	La/Ce	La/V
S1	10 Aug	11 Aug	44.7	12.2	3.7	3.2 (low)	0.67	0.28
S2	11 Aug	12 Aug	58.1	19.8	2.9	24.8 (medium, rising)	0.54	0.25
S3	12 Aug	13 Aug	115.7	37.9	3.1	65.7 (peak, dominant)	0.52	0.29
S4	13 Aug	14 Aug	104.7	30.7	3.4	54.3 (peak, dominant)	0.51	0.28
S5	14 Aug	15 Aug	84.4	22.2	3.8	42.5 (peak, dominant)	0.54	0.26
S6	15 Aug	16 Aug	64.6	13.7	4.7	12.0 (medium, dropping)	0.52	0.22
S7	16 Aug	17 Aug	74.4	13.9	5.4	2.0 (low)	0.52	0.20
S8	17 Aug	18 Aug	55.2	11.3	4.9	14.0 (medium)	0.58	0.22
S9	18 Aug	19 Aug	45.1	9.3	4.8	2.1 (low)	0.55	0.14

a Elemental mass ratios La/Ce and La/V are also provided.

b Measured by the Texas Commission on Environmental Quality (TCEQ) at the same location.

c Differences in elemental ratios during high dust days (i.e., apportioned dust ≳ 25 μg/m^3^; samples S2, S3, S4, and S5) and “regular days” (i.e., apportioned dust ≤ 14 μg/m^3^, samples S1, S6, S7, S8, and S9) are summarized in Table S2, providing evidence of North African dust influence.

### Microbial Detection, Classification, and Statistical Analyses

Nucleic acids were extracted from filters using commonly accepted protocols via bead-beating and purification and centrifugal filtration with modifications.^27,42,47,49,50^ Following quality control measures, sequence reads were organized into operational taxonomic units (OTUs) at a threshold of 97% similarity. OTU is an operational definition of a classification unit (genus, species, grouping, etc.) commonly used in population genetics to facilitate data analysis. Further information about DNA extraction, polymerase chain reaction (PCR) amplification, and data processing is in Section S14. Generalized dissimilarity modeling (GDM) was implemented in the R package *gdm* v1.5.0–9.1^57^ to characterize the mixed effects of fluctuating pollution sources and elemental compositions on microbial beta diversity across sampling days.^58,59^ Separate GDMs were developed for bacteria and fungi, with predictor variables consisting of either estimated aerosol source contributions or elemental concentrations. To test whether daily OTU abundance was phylogenetically autocorrelated, Pagel’s λ was calculated on 999 Monte Carlo simulations using the “phylosig” function in the R package *phytools*.^60^ This analysis tested the hypothesis that evolutionarily related OTUs exhibited similar abundances across days, which would be expected if the ecological tolerances of the OTUs in response to the pollution source and composition were evolutionarily conserved. The R package *ggtree*(61) was used to visualize phylogenetic patterns of OTU richness, abundance, and significant correlations to pollution sources and elemental predictors. Details on DNA extraction, PCR amplification, data processing, statistical analysis, and GDM are given in Section S14.

## Results and Discussion

### Aerosol Mass Concentrations, Elemental Composition, and Evidence for African Dust Intrusion

**Figure 1:**
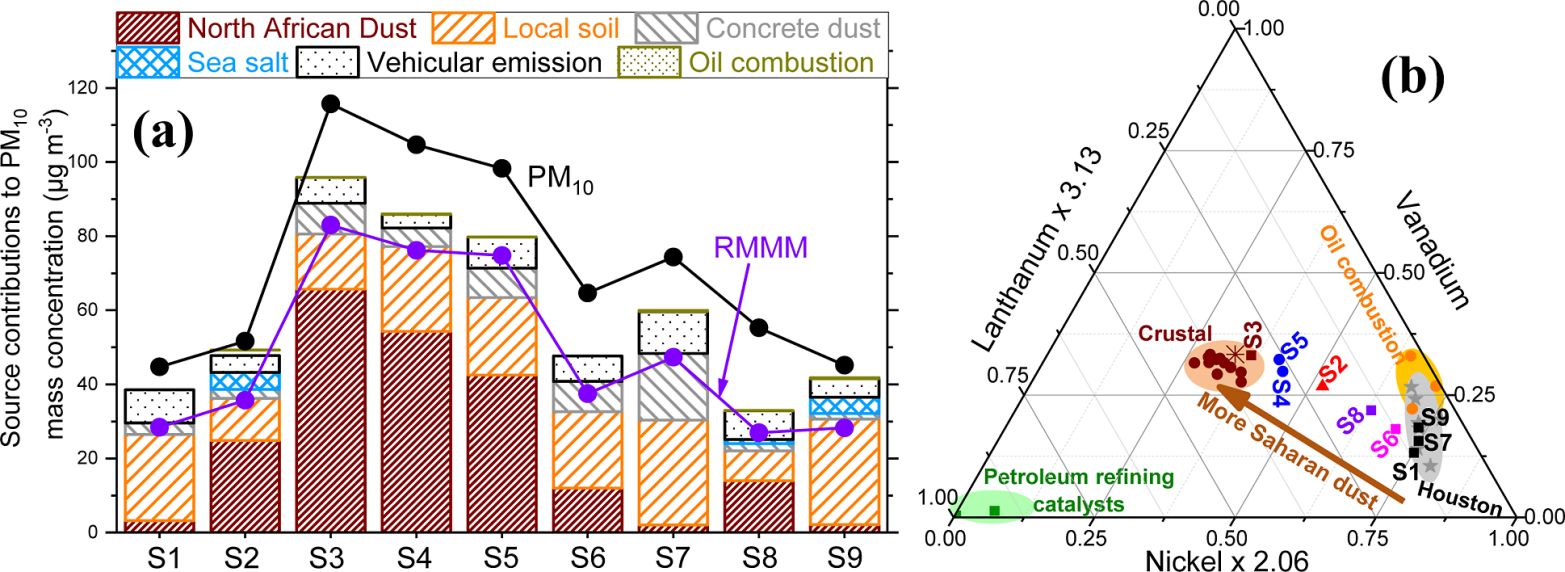
Left panel (a) summarizes the CMB modeling results of daily samples (S1–S9) depicting all source contribution estimates. Crustal material from North Africa and local Houston soil (bottom two bar stacks hatched with brown and orange lines, respectively) dominated the PM_10_ mass concentrations during this study. The reconstructed measured mineral material (RMMM, violet-colored dots and lines) compares well with the total apportioned mineral dust (African dust + local soil + concrete dust) validating their quantitative estimates. Aerosols released from construction activities (third from the bottom and labeled as “concrete dust” in gray hatches) and motor vehicles (fifth from the bottom in black dots) were the major contributors to PM_10_. Oil combustion aerosols (top stack in yellow-green dots) contributed negligibly to PM_10_ mass but substantially modified the La–V–Ni chemistry. Sea salt (fourth from the bottom in blue cross hatches) contributions are consistent with Houston’s proximity to the Gulf of Mexico coast. The gap between the PM_10_ concentrations (black dots and lines) and the top of each stacked bar represents the unapportioned mass that cannot be solely resolved by elemental analysis (i.e., secondary aerosols, which averaged 18% through this study duration).^17,63^ The right panel (b) summarizes simultaneous three-component variations in La–V–Ni where concentrations have been normalized so that the UCC values^65^ appear at the centroid using the multiplication factors 1 for V, 2.06 for Ni, and 3.13 for La. This representation accurately and quantitatively captured Saharan dust source contribution estimates evidenced by samples having lower African contributions plotting closer to the nickel apex, while days with progressively higher dust shifting closer to the UCC. Analogously, samples with greater proportions of anthropogenic nickel and vanadium^29,66^ (from high-temperature industrial oil combustion, shipping, and metals recycling) toward the beginning and end of sampling (S1, S7, and S9) moved away from the centroid, i.e., closer to the nickel apex and those with diluted local industrial emissions during the peak event (S3, S4, and S5) migrated closer to the centroid. Neither the gravimetric PM_10_ mass nor the CMB-estimated African dust mass decreased monotonically after the dust peak (Figure 1a). Specifically, S7 had higher contributions from locally resuspended soil/road dust and construction activities, resulting in a higher measured PM_10_ mass concentration than its predecessor S6. Additionally, S8 had a substantially higher apportioned Saharan mass value than its predecessor S7. In other words, not only did local emissions fluctuate day-to-day but they mixed variably with long-range transported dust. This aspect of ground-level aerosol behavior cannot be captured by satellite observations and synoptic-scale modeling, highlighting the value of elemental characterization in support of CMB modeling.^21,22^ Importantly, the La–Ni–V ternary plot faithfully captured aerosol mixing signatures at the ground level, and the location of individual samples on the straight line connecting the southeastern Ni-apex to the UCC centroid in Figure 1b was accurately captured by CMB estimates of African dust contributions (e.g., S8 migrated closer to the UCC compared to S6 and S7). Cumulatively, these results provide the necessary information to test our first hypothesis and potentially link metagenomic data with African dust (and aerosol sources).

Ambient PM_10_ concentrations jumped sharply from 45 μg/m^3^ in sample S1 (prepeak) to 116 μg/m^3^ in sample S3 (peak event) before gradually receding to routine levels in sample S9 (postpeak), which was traced to North Africa (Table 1).^21,62−64^Sections S6–S13 provide significantly more details on aerosol mass and elemental concentrations, synoptic-scale modeling to detect African dust, and its influences on the atmospheric geochemistry of Houston. In brief, the presence of long-range transported dust during our sampling campaign was indicated by a lower PM_10_/PM_2.5_ mass ratio during the peak event (average 3.4 for samples S2–S5) compared to routine days (average 4.7 for samples S1, S6–S9) as previously reported in Houston.^21,22^ Additionally, the average La/Ce ratio throughout this study was 0.55, which compared favorably with the upper continental crust and Barbados Saharan dust values of 0.50^62,65^ further evidencing dominant crustally sourced dust during the entire study. Vanadium-rich industrial oil combustion aerosols, an important source in Houston, were detected by a lower La/V ratio of 0.24 compared with a North African dust value of 0.38.^21,63^ These qualitative observations were quantitatively validated via chemical mass balance (CMB) modeling (Figure 1a) that confirmed the dominance of North African dust for samples S2–S5 contributing nearly half their measured total PM_10_ mass (average 48 ± 9%, range 34–57%) during the peak episode. Saharan dust was also detected in all other samples but in smaller amounts, contributing an average of 12 ± 9% (2–25%) consistent with its presence on days before and after the peak intrusion.^21,22^ Other sources are described in Section S5.

Figure 1b summarizes simultaneous variations in La, V, and Ni concentrations (labeled with sample numbers) after normalization by upper continental crust (UCC) abundances.^65^ These elements were chosen because V and Ni are among the most abundant metals in crude oil,^66^ and La was mostly crustally derived during this study. Concurrent variations of these components in ambient PM_10_ followed a linear trend spanning nearly the entire distance between the Ni apex and the UCC centroid, signifying that these metals mostly arise from the mixing of oil combustion emissions and crustal aerosols. As shown in Figure 1b, ternary variations in La–Ni–V spanned the distance between the Ni apex and UCC centroid in a straight line, demonstrating that these elements almost solely arose from the mixing of local anthropogenic emissions and long-range transported crustal aerosols. Samples S1, S7, and S9 that were collected at the beginning and toward the end of the campaign had the lowest amounts of African dust. Consequently, these samples were dominated by locally emitted aerosols having strong anthropogenic characteristics and moved farthest away from the centroid and closest to the Ni apex, as shown in Figure 1b. Aerosols with increasingly higher Saharan dust contributions moved progressively closer to the centroid (S6 → S8 → S2 → S4 ∼ S5 → S3, as summarized in Table 1). In other words, stronger crustal signatures associated with greater amounts of apportioned African dust mass diluted local industrial Ni emissions, thereby shifting aerosols progressively closer to the centroid. The peak event (S3) asymptotically approached the UCC coordinates corresponding to an estimated two-thirds of its mass originating in North Africa (see Table 1).

### Bacterial Community Structure

Following quality control, 16S rDNA gene sequencing generated an average of 30,981 reads per sample, varying between 20,005 and 65,154. 848 bacterial OTUs were recorded over the sampling campaign, representing 17 phyla, 46 classes, 117 families, and 121 genera. The classification of OTUs was low beyond the family level (Figures S15 and S16), especially in S2 where 59% of OTUs were unclassified even to phylum. Commonly used alpha diversity indices including Chao1 (252 ± 111) and Shannon (6.3 ± 1.6) were calculated to quantify microbial diversity (Figure S17 and corresponding discussion), which indicated substantial daily variation in the richness of rare OTUs but little variation when accounting for total OTU abundance. Bacterial rank abundance and petal diagrams are shown in Figures S18 and S19.

**Figure 2:**
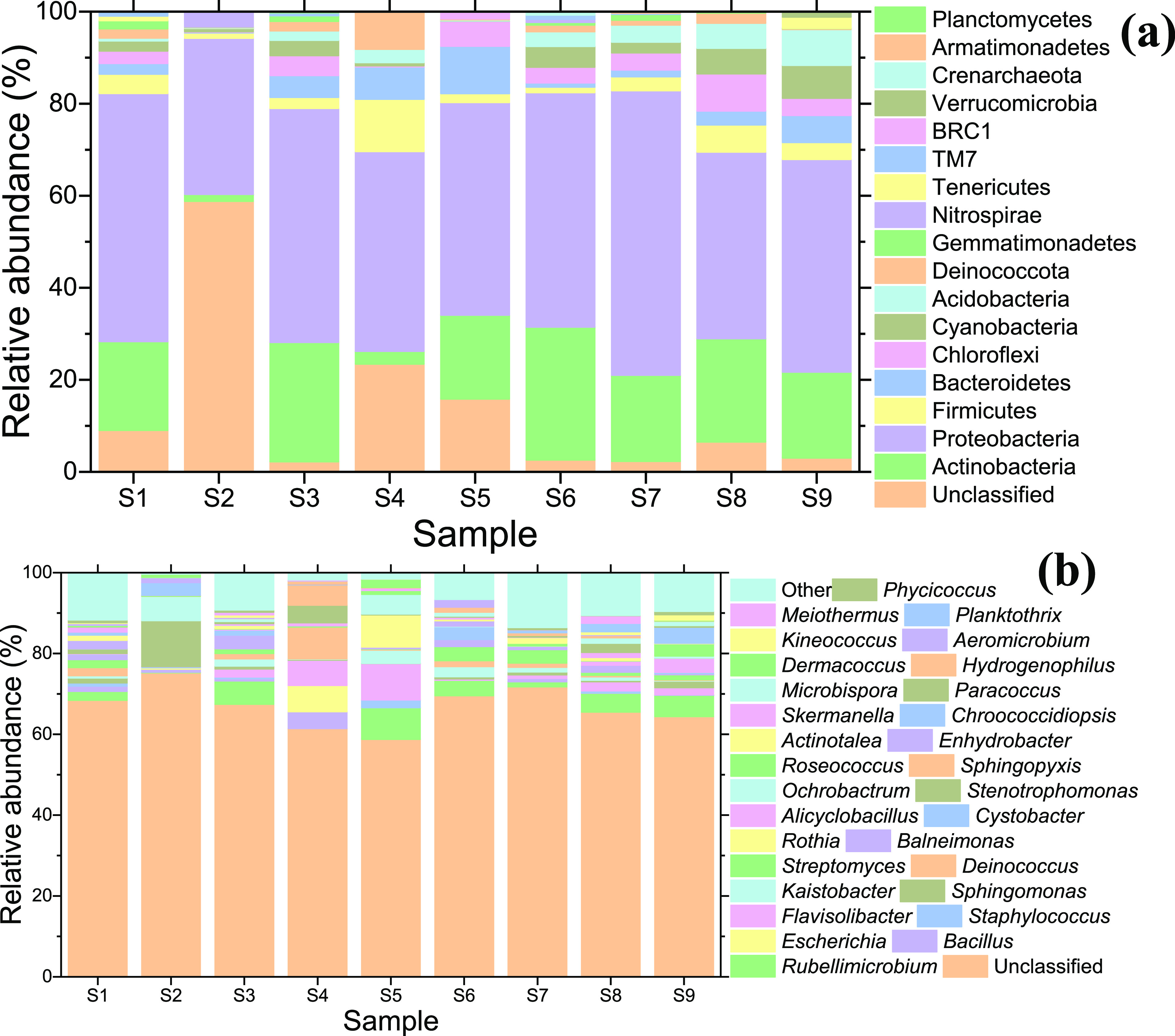
Relative abundance of bacterial phyla (top) and the top 30 bacterial genera (bottom).

As summarized in Figure 2 (and Figure S25 heatmap), the dominant bacterial phyla across all sample days were Proteobacteria (average 47.5%) and Actinobacteria (17.4%), followed by notable abundances (>5%) of Firmicutes on S4; Bacteroidetes on S4, S5, and S9; Chloroflexi on S5 and S8; Cyanobacteria and Acidobacteria on S8 and S9; and Deinococcota on S4, which generally agrees with the Saharan dust microbiome near the source^42^ and in southern Europe and Israel.^36,40,49^ Nine of the 17 total phyla were present across all sample days. Firmicutes have been previously identified in African dust^13,36,40−42,67^ and are common in airborne bacterial communities.^51^ Proteobacteria and Actinobacteria include many soil-inhabiting bacteria and are core phyla in airborne bacteriomes in studies in many parts of the world.^14,36,38,40−42,49,51,68−70^ Proteobacteria was represented predominately by the class Alphaproteobacteria^41,49^ (33%) and orders Rhodospirillales (12.6%, associated with marine sources) and Sphingomonadales (8.5%, reported in air and plant litter^71^). Actinobacteria’s largest representation was of the sporulating order Actinomycetales (14.8%), which is ubiquitous in soil and water^71^ and along with Bacteroidetes has been reported in southern Europe under Saharan influence.^36^

Dominant genera recovered in our analyses include those that have previously been linked to African dust, are pathogenic, or both. These include *Rubellimicrobium*, noted during African dust events in the Eastern Mediterranean and coastal Europe;^49,50^*Bacillus*, an animal and plant pathogen ubiquitous in deserts^14^ and Saharan dust;^36,42^*Escherichia*, a human pathogen identified in an African dust intrusion in southern Spain^36^ and in Mali;^42^*Staphylococcus,* a component of the desert soil/dust microbiome^14^ and a leading nosocomial antibiotic-resistant pathogen; *Sphingomonas*, reported in desert dust from North Africa and in the Middle East^41,72,73^ and common in the aerobacteriome;^50,70^*Kaistobacter*, a soil-inhabiting species but rarely reported in urban and dust-related bioaerosols;^74^*Deinococcus* and *Pontibacter*, previously identified in Saharan dust/soil;^14,40,75^*Streptomyces*, a plant pathogen found in desert soil/dust^14^ and decaying biomass; and *Rothia*, an opportunistic soil pathogen.

Due to their tremendous diversity (a single gram of fresh soil can contain up to 12,000 prokaryotic genomes^76^), the microbiome remains largely unclassified beyond kingdom/phylum to date. This is in part because most prokaryotic species cannot be cultured and so have remained undescribed. Using metagenomics to identify unique bacterial and fungal genomes is a very new application of emerging technologies,^77^ and it has resulted in the identification of countless unique genomes; the classification of these genomes has yet to catch up. Additionally, exposure to harsh environments (e.g., dehydration and exposure to ultraviolet radiation) during long-range transport in the ∼3–5 km high Saharan air layer can fragment the genome, resulting in microorganisms becoming “unclassified” based on available bioinformatics databases. Hence, the “unclassified OTUs” are not of much concern herein, which rather just reflects the state of the field that most microbiota are not classified beyond kingdom/phyla.

### Fungal Community Structure

ITS rDNA gene sequencing resulted in a mean of >109,000 counts per sample, which was significantly higher and less variable than that of bacteria. A total of 1345 fungal OTUs were identified representing 4 phyla, 20 classes, 69 orders, 164 families, and 286 genera. The classification of fungal OTUs was more precise than that for bacteria, with >20% of reads identified to species (Figure S16). The Chao1 and Shannon indices were 500 ± 370 and 6.5 ± 1.1 (Figure S17), respectively, demonstrating that fungi behaved like bacteria by exhibiting substantial daily fluctuations in the richness of seldom-occurring OTUs, which, however, remained relatively consistent when taking into account the total OTU abundance. Fungal rank abundance and petal diagrams are shown in Figures S18 and S19.

**Figure 3:**
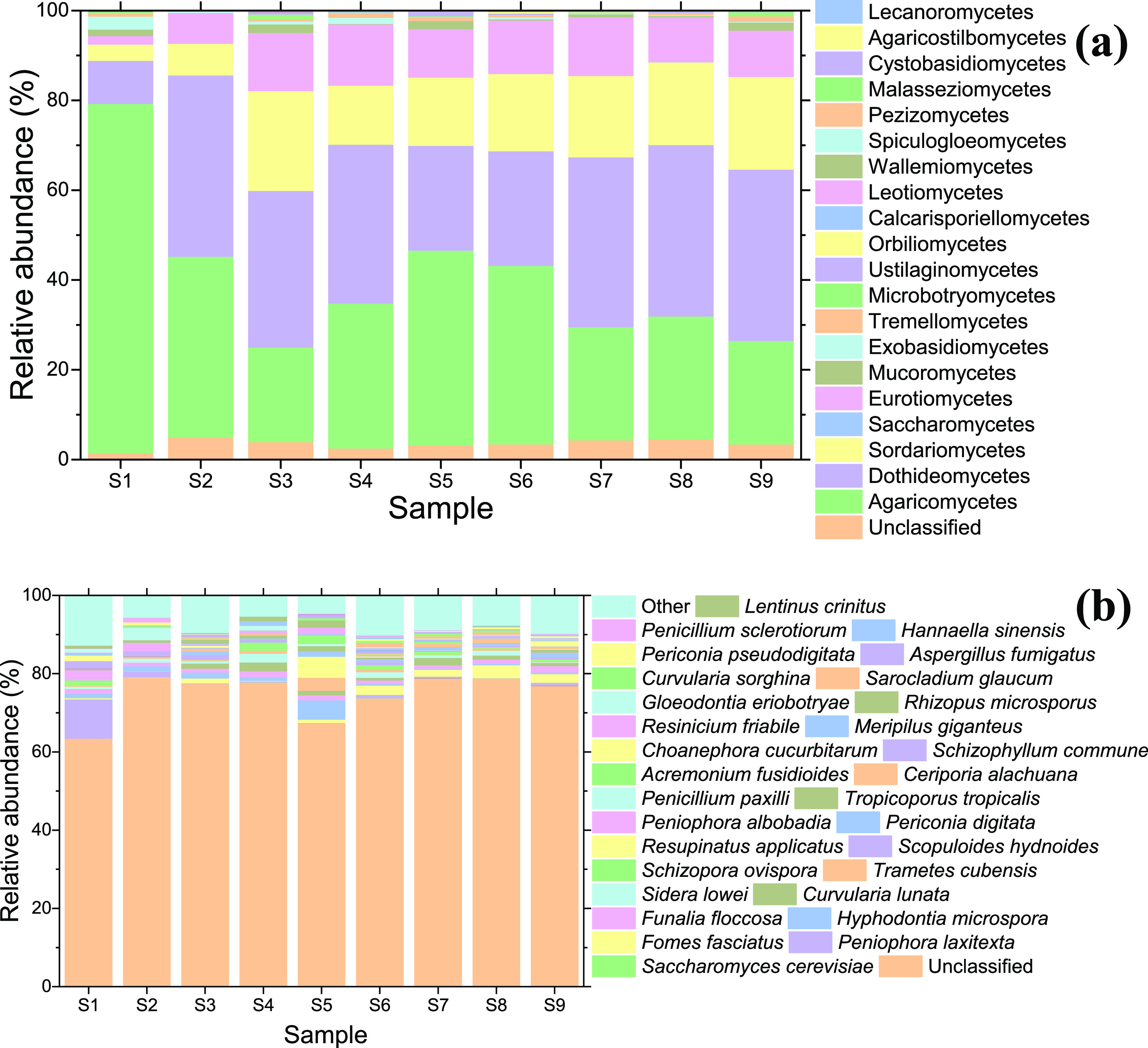
Relative abundance of fungal classes (top) and the top 30 fungal species (bottom). Note that the unclassified OTUs appear at the bottom of both (a,b). The other OTUs appear at the top of each stacked bar in (b). *Peniophora albobadia* is in column 1 of the legend, whereas *P. laxitexta* is in column 2 in (b).

**Figure 4:**
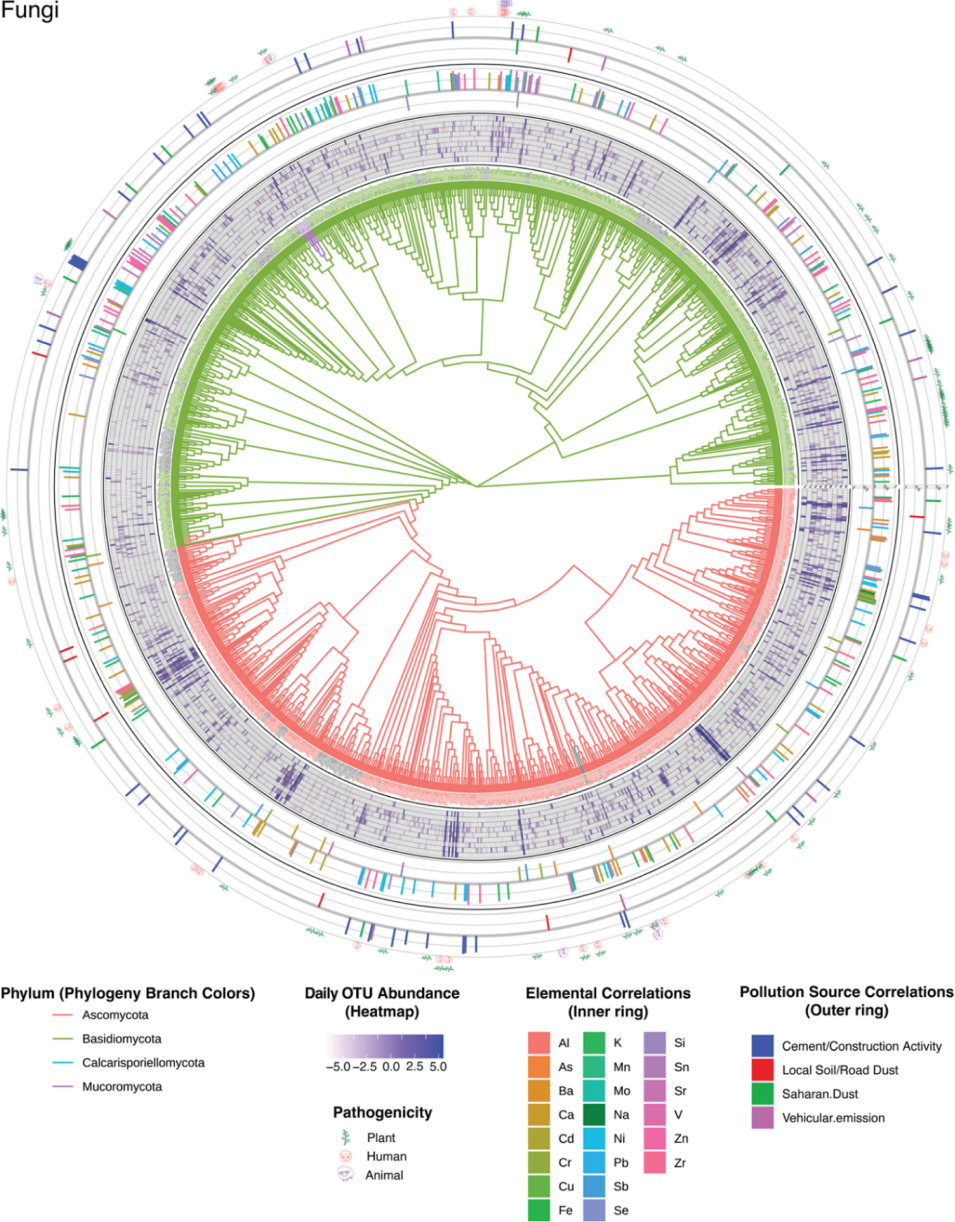
From the innermost ring to the outermost: Fungal phylogenetic tree; fungal OTU heatmap (second from center) indicating relative abundances of each OTU from samples S1 (inner) to S9 (outer); positive (pointing outward) and negative (pointing inward) correlations between elemental concentrations and fungal OTU variations (third from center); positive (pointing outward) and negative (pointing inward) correlations between aerosol pollution source and fungal OTU variations (outermost). Some OTUs were significantly correlated to more than one element or source, in which case only the strongest correlations are visualized here. Tables S8 and S9 summarize all of the significant correlations.

Most fungal OTUs were designated to Ascomycota (58.5%) and Basidiomycota (38.8%), the kingdom’s two largest phyla (Figure 4 heatmap).^27,70,78^ Dominant fungal classes (Figure 3 top panel) were Agarimicomycetes (36.7%), Dothideomycetes (31.5%), Sordariomycetes (15.1%), and Eurotiomycetes (10.1%). Agaricomycetes are a large class of wood-decaying spore-forming fungi that have been detected in aerosolized form.^56^ Dothideomycetes contain several plant pathogens as well as saprobic fungi found in soils and freshwater habitats.^79^

At the genus level, dust intrusion caused a strong dilution of *Peniphora* (Peniophoraceae), which constituted roughly a third of all measured genera on S1. *Nigrospora* (Trichosphaeriaceae), which includes several phytopathogenic species, was the most dominant genus across the sampling campaign and has been reported in Saharan-Sahelian dust.^14^ Genera that have been previously isolated from desert soils,^80^ (hyper)arid environments,^81^ desert dust,^14,46,82,83^ and in the United States households^70^ were also identified from the families Chaetomiaceae (*Chaetomium*, *Scytalidium*, and *Thielavia*), Ceratobasidiaceae (*Thanatephorus*), Cladosporiaceae (*Toxicocladosporium*), Davidiellaceae (*Cladosporium*), Mucoraceae (*Rhizopus*), Nectriaceae (*Fusarium*), Pleosporaceae (*Alternaria* and *Stemphylium*), and Thrichocomaceae (*Aspergillus*, *Penicillium*, *Talaromyces*, and *Thermomyces*). The top 30 fungal species identified are shown in Figure 3b, which includes several human and plant pathogens (listed more comprehensively in SI Table S10) as well as those that serve beneficial roles (e.g., *Curvularia* and *Saccharomyces*).

Because this is one of the first data sets to simultaneously characterize the airborne bacteriome and mycobiome for North African dust, strong conclusions cannot be drawn regarding comparisons between fungi and bacteria in Figures 2 and 3. We can expect fungal genomes to be potentially better protected from the harsh environmental conditions encountered during transport in the Saharan air layer compared with bacteria because they are more readily sporulating. This may result in a higher survival rate and potentially less variability in fungal diversity resulting from stochasticity. As seen in Figures 2 and 3, the number of unclassified bacterial and fungal OTUs tended to increase on sample days with higher dust concentrations. This may indicate the long-distance dispersal of biota from the Saharan region, where fewer microbiota are fully classified. However, we were unable to confirm this pattern with our current data set.

### Bacterial and Fungal Beta Diversity

We anticipate a high degree of day-to-day variation (beta diversity) in bacterial and fungal communities at the ground-level receptor site in terms of what might be captured on the filters. The average Bray–Curtis beta diversity among sampling days was 0.85 in bacterial communities and 0.52 in fungal communities, indicating that bacterial samples were substantially more dissimilar to each other than fungal communities over the 9 day sampling campaign. This beta diversity was visualized using principal coordinate analysis (PCoA; Figures S26 and S27). The first three principal coordinates explained ∼50% of the total variance for bacteria and ∼70% for fungi. In bacteria, the high overall beta diversity was manifested in a diffuse spread of samples across all PC axes (Figures S26a,b). The first sampling day, S1 (before dust incursion), was distinct from the remaining samples along PC3, while the last sampling day, S9 (after dust incursion), was distinct along PC2 (Figure S26b). In fungi, samples S1 and S2 were distinct from a tight cluster of the remaining samples along PC1, samples S1, S2, and S5 were distinct along PC2, and samples S1 and S1 were distinct along PC3 (Figure S27b). Additionally, the low-dust sample day (S1) prior to the major influx of North African dust (S2) was more dissimilar to the high-dust days and low-dust days following the dust event. This suggests lingering effects of dust on community composition, which have been noted in other studies of dust intrusion^20^ and are consistent with low quantities of African dust arriving before the peak intrusion and remaining after the main dust plume had migrated away. We initially reported such behavior in Houston using trace elemental measurements and more recently developed isotopic techniques to accurately estimate source contributions.^21,22^ Results and discussion of principal component analysis (PCA), a similar ordination analysis, are given in Figures S20 and S21. At this stage, we do not make strong mechanistic inferences based on this empirical observation. However, we explore the relationship between species abundance, biotic turnover, and daily variation in the elemental composition and source of pollution using linear regression and general dissimilarity modeling, as discussed below.

### Microbial and Geochemical Correlations

As the central motivation of this study was to establish a baseline understanding of the impacts of Saharan dust incursion in Houston, Texas, on local aerosolized microbial diversity, we present empirical evidence on the correlations between pollution fluctuations and variations of both alpha (i.e., number of species) and beta (i.e., change in overall community composition through time) diversity throughout the dust storm event. Determining the underlying processes (e.g., whether certain pollutants inhibit or promote the growth of particular biota or whether biota themselves travel alongside Saharan dust) is beyond the scope of this foundational study, though the evidence reported herein provides a hypothetical framework for subsequent studies examining the nexus of long-distance dispersal, local pollution dynamics, and biodiversity.

Our phylogenetic analysis recovered relationships among bacterial (Figure S25) and fungal phyla (Figure 4) that were largely consistent with previous studies, though not all phyla were recovered as monophyletic.^84,85^ Daily fluctuations in the abundance of each OTU are visualized in the heatmaps in Figures S25 and 4 (second from the innermost concentric ring), which show that some OTUs were present in high abundance in all samples, while others were present at lower abundances and in fewer samples. On all sampling days, the phylogenetic signal quantified with Pagel’s λ was lower in bacteria (0–0.26) than in fungi (0.13–0.36, Table S11). For context, a value of 0 indicates that OTU abundance is essentially randomly distributed across the phylogeny, whereas a value of 1 suggests that the phylogeny perfectly predicts abundance in accordance with a Brownian motion model of evolution. The phylogenetic signal in fungi was significantly >0 on all sampling days but was significant in bacteria only in samples S2, S5, S6, S8, and S9. These results indicate that, while alpha diversity was substantially higher in fungi than in bacteria, closely related fungal OTUs responded more similarly to daily deviations in pollution source and composition than did closely related bacteria OTUs.

Fluctuations in OTU abundances (heat maps in Figures S25 and 4) were significantly correlated (*p* < 0.05) to variations in both the aerosol elemental composition (inner ring of histograms) and pollution sources (outer ring of histograms) across sampling days (Tables S8 and S9). The abundances of 229 bacterial OTUs (27%) were significantly related to concentrations of specific elements, most notably K (*n* = 26), Zr (*n* = 13), Ni (*n* = 12), Na (*n* = 8), Se (*n* = 8), and Ca (*n* = 7) as shown in the inner correlation ring of Figure S25. The abundances of 650 fungal OTUs (48%) were also significantly related to concentrations of specific elements but a somewhat different set: Ca (*n* = 63), Ni (*n* = 57), Zn (*n* = 52), Mo (*n* = 49), Pb (*n* = 48), Sn (*n* = 47), and K (*n* = 47) (Figure 4). All examined elements were significantly related to the abundance of at least one bacterial and fungal OTU. Of the significant correlations, most had positive coefficients. The abundance of 20 bacterial and 153 fungal OTUs (11%) was negatively correlated to specific elements.

Compared to the elemental composition, fewer OTUs were significantly correlated to variations in concentrations of specific pollution sources (Figures S25 and 4, outer ring of histograms). In bacterial communities, 36 OTUs (6%) varied with emission source, including construction activity (*n* = 19), vehicular emissions (*n* = 16), Saharan dust (*n* = 5), and local road dust (*n* = 3) (outermost correlation ring of Figure S25). In fungal communities, 77 OTUs (6%) varied with emission source, including cement (*n* = 47), vehicular emissions (*n* = 41), Saharan dust (*n* = 12), and local road dust (*n* = 8) (outermost correlation ring of Figure 4). Only 10 bacterial and 13 fungal OTUs exhibited inverse relationships with contributions of pollution sources. While some PM_10_ pollution sources may inhibit the growth of particular microbial species, our purpose here is to report empirical relationships without speculating on the underlying causes of these correlations. Overall, we found no strong phylogenetic pattern of the OTUs that was significantly correlated to pollution sources or individual elements, beyond the general trend that these correlations were more frequent in the more diverse phyla.

**Figure 5:**
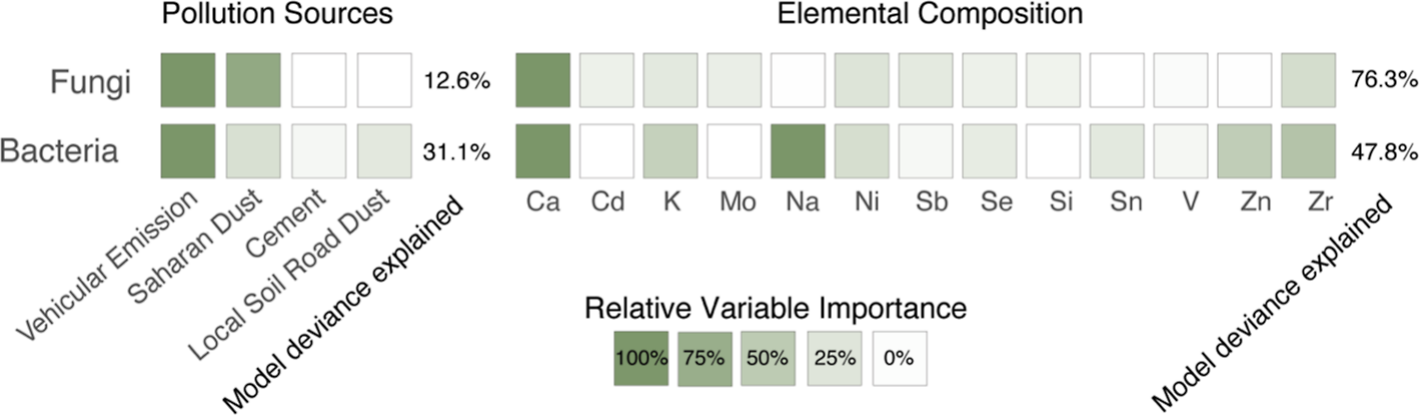
Summary of the relative importance of each element and pollution source in explaining the changes in biodiversity observed across the sampling period.

GDM was used to identify associations between bacterial and fungal community beta diversity across days with fluctuations in aerosol pollution source (abbreviated as pGDM) and elemental composition (abbreviated as cGDM) and is summarized in Figure 5. Unlike the analyses associated with Figures S25 and 4 that linked changes in individual OTU abundance to distinct explanatory variables, GDM examines total changes in community composition, accommodating both the collinearity of variables and the nonlinearity of the relationship between the ecological distance and turnover. The cGDMs explained a greater proportion of model deviance (bacteria: 47.8% and fungi: 76.3%) than the pGDMs (bacteria: 31.1% and fungi: 12.6%), likely a result of the greater explanatory power of the more numerous independent variables in the model. The bacterial pGDM performed substantially better than the fungal pGDM, while the opposite trend was observed in the cGDMs. Notably, daily fluctuations in elemental composition, but not pollution sources, were a strong predictor of fungal turnover in our data set. For both fungi and bacteria, vehicular emissions and Saharan dust contributions were most important for explaining beta diversity across days. In other words, fungal and bacterial communities were more different from day to day depending on variations in the estimated influences of vehicle emissions and Saharan dust than on variations in local road dust emissions. For fungi, Ca and Zr were the elemental variables most important for explaining beta diversity, whereas Ca, Na, K, Zn, and Zr were most important for explaining bacterial turnover (Figure 5).

### Overall Trends and Associations

Collectively, although our measurements enable us to draw statistically significant inferences related to biodiversity, emission sources, and elemental composition, we interpret them strictly as correlative and not causal. Nonetheless, several key patterns emerge. First, the majority of bacterial and fungal OTUs did not show a significant correlation in their daily abundances to concentrations of apportioned PM_10_ sources, highlighting the complexity of linking the occurrence of microbiota to specific polluters. On the other hand, substantially more of the OTUs were sensitive to individual elements than they were to pollution sources. While these may reflect the limitations of ecological tolerances of individual OTUs (i.e., some elemental concentrations might promote growth of one species, while inhibiting another), we suggest that gross patterns of OTU abundance are more likely related to other processes, such as local competition, microclimate, and weather, or simply stochasticity in the airborne microbiome, at least across a 9 day period in the Houston metropolitan area.

Second, the abundances of relatively few (*n* = 17) individual bacterial and fungal OTUs were significantly associated with measured concentrations of Saharan dust. These 17 OTUs, insomuch as they can be genetically identified, all belong to taxonomic families and phyla that are cosmopolitan in distribution. Thus, it remains unclear whether the closest relatives (i.e., geographic source of origin) of the OTUs are Saharan, given the paucity of data from North Africa. Indeed, given the collinearity between aerosol sources and elemental concentrations (Figure S22), the significant relationships between individual OTU abundances and concentrations of Saharan dust could even be an artifact. Because “crustal” elements such as Al, Ti, and Si typically signifying dust are also emitted locally in Houston (e.g., industrial operations and soil/road dust), a lack of correlation between these metals and microbial diversity does not affect the likelihood of African dust impacting the bacterial and fungal communities.^29,86^

Third, fungi and bacteria show distinct patterns in their daily alpha and beta diversity, phylogenetic signal, and turnover responses to the pollution source and elemental composition. Fungal communities exhibited far higher alpha diversity but lower beta diversity than did bacterial communities. In both simple linear regression and composite GDMs, concentrations of airborne calcium emerge as the most important element for explaining daily fluctuations in fungal OTU abundance (Figure S22) and community turnover, but this is just one of several elements that are important for explaining the higher turnover in bacterial communities (Figure 5). Calcium concentrations are themselves strongly correlated to the source contributions of vehicular emissions and cement, which were important predictors of the abundance of the OTU and overall compositional turnover. Further, calcium levels and mineralogy in desert dust are highly variable both in source regions and receptor locations.^21,62,87^ Na, K, Zn, and Zr, elements associated with Saharan dust concentrations, were important predictors of turnover only in the bacterial cGDM, although Saharan dust was recovered as important for both pGDMs. Fungal communities also showed greater and more consistent phylogenetic signals than bacterial communities, indicating that closely related fungal species exhibit more similar responses to daily fluctuations in explanatory variables than bacteria.

Notably, most of the literature linking aerobiome diversity to pollution sources or chemical composition has examined these dynamics across large spatial domains and longer seasonal variations. These studies have consistently found that the microbiome community composition is strongly influenced by season^88−90^ wherein both the diversity and turnover of biota were based on the time of year. Herein, we provide ample evidence that daily fluctuations in airborne bacterial and fungal communities are also highly dynamic, even across nine consecutive days. This is due in large part to the ubiquity of aerosolized microbial communities on a global scale as well as their local stochasticity.^12,36,51,70,78^ Nevertheless, our models suggest correlations between biodiversity and environmental conditions. The underlying causal structure of this correlative framework, however, remains obscure.

Finally, we have only limited evidence to suggest that the Saharan dust incursion into Houston substantially altered the diversity of local airborne fungal and bacterial communities. Of the 2193 OTUs identified in this study, only 17 showed a significant increase in abundance as dust entered Houston. Each of these 17 taxa is cosmopolitan, and several taxa were detected in our samples in low concentrations before Saharan dust was notably present. While GDMs suggest that Saharan dust concentrations are important for predicting fungal and bacterial beta diversity, this effect is more likely associated with the interactive effects of the dust on local dynamics rather than purely the transcontinental migration of biota.

### Potential Health and Environmental Impacts

Beyond examining links between Saharan dust and aerosolized biodiversity, we also identified the presence of multiple opportunistic human, plant, or animal pathogenic bacterial/fungal species in the study area (Table S10 and Figure S23). These include bacteria such as *Escherichia coli*, *Propionibacterium acnes*, *Roseomonas mucosa*, and *Haemophilus parainfluenzae*. Several genera listed in the World Health Organization’s (WHO) global priority pathogens list of multidrug- and antibiotic-resistant bacteria and tuberculosis^91^ were detected including *Acinetobacter*, *Enterococcus*, *Haemophilus*, *Mycobacterium*, *Pseudomonas*, and *Staphylococcus*. Other pathogens measured included Gram-negative *Achromobacter*, *Caulobacter*, *Chitinophaga*, *Chryseobacterium*, *Clostridium*, *Corynebacterium*, *Gordonia*, *Listeria*, *Megasphaera*, *Nocardioides*, *Ochrobactrum*, *Propionivibrio*, *Sphingomonas*, *Stenotrophomonas*, and *Williamsia* and the Gram-positive *Actinomyces*, *Bacillus*, *Micrococcus*, *Nocardioides*, *Propionibacterium*, *Roseomonas*, and *Rothia*. The plant pathogen *Erwinia* and the filamentous cyanobacterium *Planktothrix* capable of seeding harmful algal blooms were also detected as was *Rhizobium*, a nitrogen fixer and *Streptomyces* an infrequent plant/human pathogen.

Fungi responsible for invasive human diseases such as *Aspergillus fumigatus*, *Fusarium* spp., and *Talaromyces* spp. that are listed as being priority pathogens for global public health by WHO^54^ appeared in nearly every sample along with all four most prominent allergenic fungal genera, viz., *Alternaria*, *Cladosporium*, *Aspergillus*, and *Penicillium*.^39^ Their total relative abundance peaked in S3, the date of highest dust loading (Figure S24). Numerous genera of plant or human pathogenic fungi, including *Cercospora*, *Curvularia*, *Flavodon*, *Fomes*, *Fuscoporia*, *Funalia*, and *Magnaporthe* were also detected. Several pathogenic species peaked in abundance synchronous with North African dust, including *Curvularia lunata*, *Rhizopus microsporus*, *Penicillium sclerotiorum*, *Phellinus gilvus*, *Exserohilum rostratum*, *Aureobasidium pullulans*, and *Curvularia trifolii*. We also detected saprobic fungi that play a critical role in carbon cycling, such as *Basidiomycota*, *Neurospora*, *Peniophoraceae,* and the species *Phanerochaete chrysosporium* and *Trametes versicolor*.

(Poly)extremophilic and thermophilic genera of bacteria (*Chroococcidiopsis*, *Deinococcus*, *Hydrogenophilus*, *Meiothermus*, and *Saccharomonospora*) and fungi^80^ (*Alternaria*, *Aspergillus*, *Chaetomium*, *Cladosporium*, *Coprinopsis*, *Penicillium*, *Rhizopus*, *Scytalidium*, *Stemphylium*, *Talaromyces*, *Thanatephorus*, *Thermomyces*, *Thielavia*, and *Wallemia*) were also present suggesting their viability at receptor locations even after spending several days in the Saharan air layer.^34,55^

## Implications and Future Work

Our study attempted to identify the sources of both the inorganic and microbiological components in aerosols collected in Houston, Texas. To that end, we characterized the elemental composition in detail and coupled it to DNA sequencing. Our study is unique because aerosols in southern Texas are often impacted by African dust, allowing us to characterize the effects of advected intercontinental African dust on the aerosol elemental composition and microbial community in a populous industrialized metroplex on the North American mainland. Both fungi and bacteria were evaluated because they can be dispersed long distances, cause disease, and antibacterial and antifungal resistance is on the rise globally.^34,53,54,91^ To the best of our knowledge, this is the first rigorous simultaneous investigation of the effects of transatlantic intercontinental transport on microbial population and metals variability.

It was difficult to quantitatively delineate North African influences on bacterial and fungal communities, partially because of the mixing of locally emitted bioaerosols. Various measures of prokaryotic and eukaryotic diversity, phylogeny, composition, and abundance did not strongly or uniquely correlate with African dust mass (i.e., we did not provide strong evidence to support or refute our first hypothesis that North African intrusion modified the aerobiome). Nonetheless, microbial diversity, phylogenetic signal, and turnover responses were strongly interlinked with apportioned sources (especially construction activities) and elemental composition (especially calcium). Hence, we obtained evidence to support our second hypothesis that certain bacteria and fungi would correlate with elemental concentrations. However, we could not provide insights regarding the underlying mechanisms connecting aerosol chemistry and microbiology. Importantly, our studies might be applicable to other United States metroplexes under the influence of North African dust because the dust-associated microbial communities are thought to be relatively homogeneous in urban areas across its contiguous 48 states.^70^

This short-term study provides the foundation for designing future investigations over longer time scales to better discriminate between long-range transported microorganisms and those from local sources, i.e., by collecting size-resolved samples when local and regional sources dominate aerosol composition (e.g., November-April in Houston) and comparing them to samples collected during the active Saharan dust season [e.g., in the Caribbean Basin and southern North America (June-September)].^15,20,41^ It is emphasized that because fungi are typically larger than 2.5 μm and airborne bacteria are typically aggregated,^42^ collecting PM_1_ or PM_2.5_ might omit a substantial fraction of these microorganisms whereas PM_10_ collection should resolve this issue.

Future research also needs to complement metagenomically identified bacteria and fungi by monitoring culturability to better assess microorganisms’ role in ecosystem, human, plant, and animal health.^12,14,36,46,92−94^ Such studies should also measure other water-soluble aerosol components and metal speciation, some of which might affect viability. Additionally, airborne pollutants have the capacity to influence aerobiome abundance, diversity, transport, and deposition, and conversely, bioaerosols can facilitate the transport and deposition of airborne pollutants.^95,96^ The interplay between bioaerosols and anthropogenic emissions is complex and dependent on multiple factors, including pollutant type, concentration levels, and meteorological parameters. Longer-term measurements, including microbial viability, can provide clues to such interactions. Our work also suggests that it might be important to investigate the oxidative potential of bioaerosols in conjunction with metals. Together, they could generate reactive oxygen species, which are thought to be a good indicator of toxicity.^4,5,8^ This work could also be useful in identifying mineral dust and bioaerosols that are nuclei for cloud condensation and ice formation, thereby influencing the radiation budget, climate, and the hydrological cycle.^2,12,56^

Finally, it is being increasingly recognized that climate change has contributed to changes in both dust quantity and the concentrations of certain inorganic species as exemplified in the five decades since monitoring began in Barbados.^20,97^ Modeling suggests that the global-scale transport of the plant pathogenic soilborne fungus *Fusarium oxysporum* is very sensitive to wet and dry removal processes.^34^ However, analogous information is not available about the associated microbiological variability. Measurements like ours are needed over longer timeframes, which can serve as a baseline with which to elucidate future changes. The strategy used in our study can be directly implemented to study Saharan dust transported to other receptor regions, e.g., South America,^98−100^ the Middle East, and Europe, all of which are frequently impacted by African dust. In these cases, the dust may originate in different African source regions, travel by different paths, and occur in different seasons.^99,101^ This is particularly important because of the interplay between climate, which influences the potential African source areas (and thereby its characteristics) and the role of dust in climate forcing.^12,40^

## Supporting Information Available

Information on instrument setup, quality control details, blanks and detection limits, CMB source apportionment details, satellite and aerosol model images, elemental concentrations and ratios measurements, additional methodology for nucleic acid extraction, gene sequencing, statistical analysis, bioinformatics including relationships between OTUs and sources/elements, and list of pathogenic bacteria/fungi and their presence over the entire sampling period are provided (PDF)
