# Supplementary material for: Respirable Metals, Bacteria, and Fungi during a Saharan–Sahelian Dust Event in Houston, Texas
Source: Environ Sci Technol. 2023 Nov 9;57(48):19942–55. doi: 10.1021/acs.est.3c04158 (PMC10862556; doi:10.1021/acs.est.3c04158)
Supplement: Supplementary file 1 — es3c04158_si_001.pdf [file es3c04158_si_001.pdf]

Supporting Information

Respirable Metals, Bacteria, and Fungi during a Saharan-Sahelian Dust Event in Houston, Texas

Sourav Das[a], Alyvia McEwen[a], Joseph Prospero[b], Daniel Spalink[c], and Shankararaman Chellam[a,d,]*

[a] Department of Civil & Environmental Engineering, Texas A&M University, College Station, TX, 77843, USA

[b] Rosenstiel School of Marine and Atmospheric Science, University of Miami, Miami, FL, 33149, USA

[c] Department of Ecology and Conservation Biology, Texas A&M University, College Station, TX, 77843, USA

[d] Department of Chemical Engineering, Texas A&M University, College Station, TX, 77843, USA

* Professor and holder of the A.P. & Florence Wiley Professorship III and corresponding author, Zachry Department of Civil & Environmental Engineering, Texas A&M University, College Station, TX 77843-3136, USA. Phone: (979) 458 5914; chellam@tamu.edu

Number of pages: 77

Number of figures: 27

Number of tables: 12

SI Table S0. Instrumental settings and operating parameters for DRC-ICP-MS (PerkinElmer® NexION® 300 with ammonia as the cell gas).

Instrument	PerkinElmer® NexION® 300
Nebulizer	Concentric (Meinhard) [Type A0.5]
Spray chamber	Baffled quartz cyclonic
Torch injector	Quartz
Auto lens	On
RF power	1600 W
Sample	18 L min^{-1}
Nebulizer	0.96-1.18 L min^{-1}
Auxiliary	1-1.2 L min^{-1}
Interface	Platinum cones
Sampler cone	1.1 mm orifice diameter
Skimmer cone	0.9 mm orifice diameter
Scanning mode	Peak hopping
Sweeps/reading	20
Readings/replicate	1
Replicates	3
Dwell time	50 ms (standard mode); 100 ms (DRC mode)
Sampling parameters	AS-93 plus auto-sampler
Sample flush time	45 s
Sample flush pump speed	20 rpm
Read delay	65 s
Read delay pump speed	24 rpm
Wash time	60 s
Wash pump speed	24 rpm
Cell gas	NH_3 (0.2-1.0 mL min^{-1})
RPq	0.25-0.75
RPa	0

Section S1. Quality Assurance and Quality Control (QA/QC)

Ten milligrams of two National Institute of Standards and Technology Standard Reference Materials (SRMs) – SRM 1648a (urban particulate matter) and SRM 1633b (Coal Fly Ash) were digested and their elemental composition measured by ICP-MS using identical methods as aerosol samples. This served as a quality assurance/quality control (QA/QC) check. All elements from SRM 1648a and SRM 1633b were quantitatively recovered (86 – 112%) in comparison with National Institute of Standards and Technology (NIST) provided values (**SI Figure S1a and SI Figure 1b,** respectively) validating our sample digestion and instrumental analysis techniques.

^{72}Ge, ^{115}In, and ^{209}Bi were used as internal standards to monitor ICP-MS performance and sensitivity drift. Apart from internal standardization, this also provided with analysis of duplicate samples; thus, verifying measurement repeatability and precision. Blanks were measured made at regular intervals to check any carryover and baseline intensity of each element. Baseline of each measurement was calculated by interpolation of the values obtained from intermediate blank sample analysis. A blank filter was placed inside the sampling instrument as a negative control and its elemental concentrations were subtracted from each sample herein. Additionally, standards whose concentrations were equal to that of the mid-point of our calibration graph were prepared and analyzed at regular intervals to monitor measurement uncertainties. An acceptable range of ±10% uncertainty was adopted for drift check.

Polyatomic interferences in Cd, Ga, and other trace metals were removed by measuring the interfering isotope and is corrected by a mathematical formula that was empirically developed by running several elemental standards at the time of instrument setup.

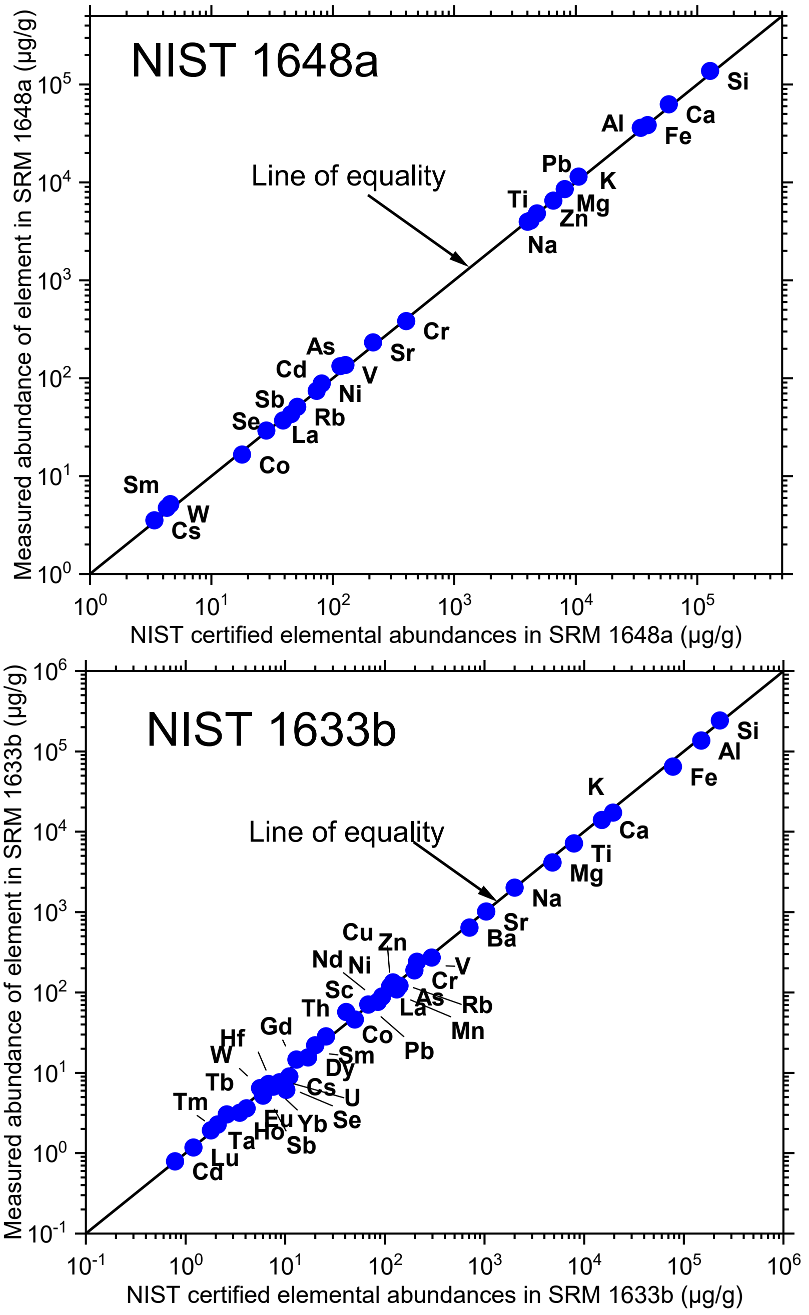

SI Figure S1. Quantitative recovery of all elements from National Institute of Standards and Technology Standard Reference Materials NIST 1648a and NIST 1633b validate our microwave dissolution and ICP-MS analysis procedures.

SI Table S1. Filter blank values. Values with less than symbol "<" notifies below detection limit.

Element	Blank filter concentration (ng m^{-3})
Na	53 ± 22
Mg	2 ± 1
Al	8 ± 2
Si	<109
K	<4
Ca	125 ± 66
Ti	<0.69
V	<2.5
Cr	<0.31
Mn	<0.09
Fe	<5.3
Ni	<0.09
Co	<0.16
Cu	<0.01
Zn	<0.03
Ga	<0.16
As	<0.13
Se	<0.34
Rb	<0.13
Sr	<0.01
Y	<0.09
Zr	<0.13

Element	Blank filter concentration (ng m^{-3})
Mo	<0.13
Cd	<0.13
Sn	<0.13
Sb	<0.13
Cs	<0.09
Ba	<0.69
La	<0.04
Ce	<0.05
Pr	<0.02
Nd	<0.01
Sm	<0.01
Eu	<0.01
Gd	<0.01
Tb	<0.01
Dy	<0.01
Ho	<0.01
Er	<0.01
Yb	<0.01
Lu	<0.01
Pb	<0.16
Th	<0.04
U	<0.03

Section S2. Site information and sample preparation and handling

The Clinton Drive site is located in a densely populated and economically depressed area (26.4% poverty rate) inhabited largely by ethnic minorities (91% Hispanics, African Americans, American Indians, and Alaska Natives) bringing environmental justice issues into focus. Clinton Drive is close to several heavily trafficked roads including Interstate-610 and Interstate-10, which had annual average daily traffic (AADT) counts of 178,800 and 194,945 vehicles, respectively and is within the Houston Ship Channel region, a hyper-industrialized section of the city. Therefore, air quality indices are frequently in the unhealthy range at this site, which consequently has been the target of many investigations [1-4].

A ThermoFisher 2025i Partisol sequential air sampler was used to collect samples on PTFE filters. All sampler parts (sampler inlets, filter cassettes, filter cartridge, carrier box support, and filters) were disinfected with 70% ethyl alcohol followed by 5% hypochlorite solution. Filters were disinfected by exposing them to UV light for 15 minutes inside a biosafety cabinet (Baker SterilGARD, Class II Type A2). Filters were refrigerated at -80°C immediately after removing from the sampler (using sterile tweezers), weighed to measure PM_{10} mass concentrations, cut into two halves using a sterilized ceramic knife inside a biosafety cabinet (one for elemental analysis and the other for sequencing), and again stored at -80 °C in sterile petri dishes until further analysis.

Note that only PM_{10} was sampled in this study. Correspondingly, the PM_{10} inlet was always attached to the sampler and a by-pass tube was inserted to replace the $PM_{2.5}$ inlet as is always practiced for these samplers. Aerosols were not dried, but samples were immediately cryopreserved at -80 °C as commonly practiced for medium- to long-term storage.

Section S3. Elemental analysis

The filter half designated for elemental analysis was digested first in 3 mL of trace metal grade concentrated HNO3 with additional optima grade concentrated HF in a ratio of 0.3 mL for every 10 mg of sample, based on our previous work [2, 3, 5, 6]. This was followed by adding 5% w/v boric acid solution to mask the insoluble fluoride complexes and was re-digested. Each stage was performed at 200°C and

200 psig in a microwave oven (MARS 6, CEM Corporation) for a dwell time of 20 min. The digested aliquot was then diluted to 2% nitric acid matrix before analyzing with Inductively Coupled Plasma – Mass Spectrometry (ICP-MS), with instrumental settings summarized in Supplementary Information (SI) Table S0. Forty six elements were measured including 30 from Groups 1-16 (Na, Mg, Al, Si, K, Ca, Ti, V, Cr, Mn, Fe, Co, Ni, Cu, Zn, Ga, As, Se, Rb, Sr, Zr, Mo, Cd, Sn, Sb, Cs, Ba, Pb, Th, and U) and 16 rare earths (Sc, Y, La, Ce, Pr, Nd, Sm, Eu, Gd, Tb, Dy, Ho, Er, Tm, Yb, and Lu). Al, V, Cr, Fe, Ni, Cu, and Zn were analyzed in Dynamic Reaction Cell (DRC) mode using ammonia as the cell gas to reduce polyatomic interferences [2, 5-8]. More information including instrument settings and quality control information above and in in earlier publications [2, 5, 6, 8].

Section S4. Evidence of North African Dust from Satellite and Software Products

Days of significant dust intrusion were first qualitatively deduced using publicly available atmospheric products such as TERRA and AQUA satellite images [9], dust Aerosol Optical Depth (AOD) data by United States Navy's NAAPS model [10], HYSPLIT back trajectory models [11, 12], and the Global Modeling and Assimilation Office (GMAO) atmospheric composition maps [13] before quantitatively evaluating source contributions as described next.

Section S5. Source Apportionment

The United States Environmental Protection Agency's Chemical Mass Balance (CMB v8.2) model was employed to quantify contributions of various sources to PM_{10} mass concentrations [2, 3, 14, 15]. Na, Mg, Si, K, Ca, Ti, Co, Cu, As, Se, Sr, Sn, Sb, Mo, Ba, Pb, Al, V, Cr, Fe, Ni, Zn, Y, La, Ce, Pr, Nd, and Sm were chosen as fitting species based their importance as elemental tracers and level of uncertainty in our laboratory measurements. Sb, Cu, Cd, Sn, Pb, Mo, As and Zn were selected as traffic-related metals [6, 15, 16], Zn, Pb, Cr, Mn, Mg, Co, and Ni were selected to track high-temperature industrial processes [8], rare earths (La, Ce, Nd, Pr, Sm, Gd, and Eu) were chosen for petroleum refining activities (fluidized bed catalytic cracking or FCC) and crustal matter [4, 6, 7], V and Ni were chosen for

oil combustion and shipping activities [17, 18], and Al, Si, Ti, Ca, Fe and Y were used to isolate crustal mass [3, 6].

The chemical composition of Houston's local soils was established through a comprehensive analysis of twelve samples collected in various sampling locations in Houston (in and around the receptor site). Detailed descriptions of this composition can be found in our previous publications [3, 6, 19]. The CMB model takes into account variations in soil composition, and these differences are reflected in the final results. Uncertainty in the composite soil profiles not only propagates into the CMB model's uncertainty output but also influences the results due to the model's reliance on a variance-weighted linear regression. This is a core aspect of CMB modeling, which not only requires average concentrations but also their uncertainties as input. Nevertheless, we acknowledge the potential for heterogeneity in Houston soil sources, and as a result, the findings presented in this study (or in any study using CMB modeling) are labeled as "estimates" and are not presented as absolutely guaranteed accuracy. The CMB model we used accounts for the differences in soil composition and includes these variations in the final quantitative estimates.

The CMB model is based on an effective-variance least squares method (EVLS) and is weighted based on uncertainties associated with elemental concentrations in air as well as in source profiles. Selection of species/elements that goes into this model determines the accuracy of the output. Note that potential source areas in Africa are heterogeneous inducing substantial variations in the dust composition based on where they originate from [20-24]. Hence, dust storms are not all the same and may differ in ratios of crustal elements. For this reason, we sampled aerosols in Barbados during the same dust storm that we captured in Houston and used its composition as African dust source profile for source apportionment [25]. In other words, the elemental composition of African dust input to the CMB model was measured for aerosols captured in Barbados from the same dust storm after the dust cloud traversed most of the Atlantic Ocean (not directly from the Sahara-Sahel African region). Note that dust travels from Northern Africa and passes through Barbados a few days before reaching southeastern United States

as shown in back trajectories, satellite images, and synoptic scale models shown below in SI Section S7. In any event, the physicochemical characteristics of transatlantic dust are different from Saharan source areas because of size fractionation and influence of marine aerosols during long distance transport. But importantly capturing the same dust event largely eliminated uncertainties induced by weathering, size fractionation, or spatiotemporal heterogeneity.

CMB estimated sources other than North African dust that are only briefly mentioned in the main manuscript due to length constraints. They included resuspended local soil/road dust, which was the second most dominant source, contributing 31±16% (13–63%) over the study duration. Construction activities and vehicular emissions also had significant impacts averaging 9±6% (3–24%) and 11±5% (3–20%), respectively. Although oil combustion modified V atmospheric chemistry as described in the previous section, it contributed only negligibly to PM_{10} mass 0.18±0.25% (0–0.8%). Sea salt was another minor contributor to mineral matter, contributing an average of 2.3±3.8% (0–9.7%).

The RMMM is a stoichiometric reconstruction of mineral mass in our PM samples as explained in our earlier publications [2, 3, 25]. This gap between RMMM and PM_{10} in Figure 1a is completely logical and not because of modeling errors. Most of the gap arises due to the presence of non-metallic secondary aerosols that are formed *in situ* in the atmosphere, which is a common feature in the summertime and in Texas. These secondary particles are formed due chemical reactions in atmosphere, forming compounds such as ammonium sulfate and ammonium nitrate as well as secondary organic aerosols. Secondary PM is devoid of metals. A smaller portion of the gap is attributed to non-mineral PM emitted locally. Please note that to establish modeling accuracy, we should focus on the gap between the RMMM symbols and the bottom three components of each bar in Figure 1a (Saharan dust, construction activities, and local soil). As can be visually observed, there is close agreement between the apportioned mineral dust sources and RMMM, validating our CMB results. The mineral mass closely follows the total sum of African dust, local soil, and construction dust, which are enriched in mineral elements such as Na, Al, Ca, Ti, Mg, K, Fe. Uncertainties in measurement of both PM as well as

calculation of RMMM can also cause some discrepancy. Uncertainty in RMMM may include errors arising from both elemental measurement as well as the assumption that all mineral elements are present is their most stable oxide form. In any case, the majority of the difference between CMB apportioned mass and total measured PM_{10} mass indicates the presence of secondary PM, which lack metals.

Section S6. $PM_{2.5}$ and PM_{10} concentrations over the duration of the study period and analysis to deduce presence of African dust

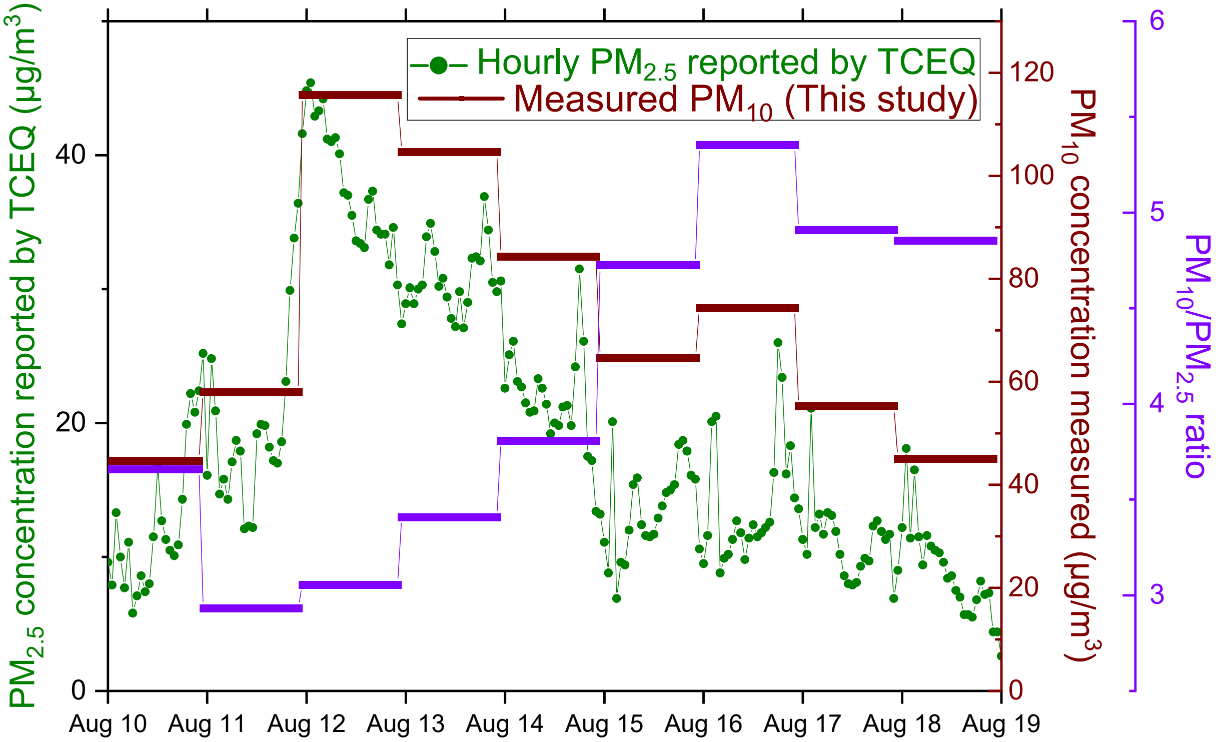

SI Figure S2. Daily PM_{10} concentrations measured in this study depicted in brown color using the first right y-axis. Hourly $PM_{2.5}$ concentrations measured by the Texas Commission on Environmental Quality are depicted as green dots using the left y-axis. The $PM_{10}/PM_{2.5}$ ratios are also shown in violet color using the second right y-axis.

$PM_{2.5}$ exhibited similar trends as PM_{10} increasing ~3-fold in sample S3 (August 10-11) from sample S1 before progressively declining to routine levels in sample S9 (August 18-19). Presence of long-range transported dust during our sampling campaign was also validated by the observed decrease of the $PM_{10}/PM_{2.5}$ ratio during the peak event (average 3.4 for S2-S5, collected between August 11-15) compared to routine days (average 4.7 for S1, S6-S9, collected between August 15-19) as we have previously reported in Houston [3, 25]. Additionally, as summarized in Table 1 of the main manuscript,

the average La/Ce ratio of 0.55 during this study compared favorably with the upper continental crust and Barbados African dust values of 0.50 [5, 26]. In contrast, the La/Ce ratio for petroleum refining aerosols are significantly higher, even greater than 1 [4, 8, 18, 19, 25, 27]. Because La/Ce ratios were approximately equal to 0.5, emissions from local petroleum refineries [4, 8, 18] were deemed insignificant and rare earths were dominated by other sources throughout the sampling campaign, which was confirmed by CMB and La-Ce-Sm ternary analysis (SI Section S11). Note that our research team has pioneered the use of light lanthanoid ratios to analyze and interpret variations in crustal and oil refining aerosols in atmospheric chemistry [4, 8, 18, 19, 25, 27, 28].

An additional consideration is that oil combustion, a well-recognized industrial source in Houston, releases vanadium-rich (and nickel-rich) aerosols thereby reducing the La/V ratio compared with crustal dust [2, 3, 8]. This was manifested consistently during the sampling campaign where the average La/V ratio in Houston was 0.24 (Table 1 in the main manuscript), which is lower than the North African dust value of 0.38 [2, 3]. Reduced La/V ratios in Houston aerosols compared to measurements for transatlantic Saharan dust in Barbados reveal local V contributions from industrial oil combustion operations.

Section S7. Detecting African Dust Using Aerosol Products and Satellite Images.

Following up on $PM_{10}/PM_{2.5}$ ratios, Saharan-Sahelian dust's presence was qualitatively inferred based on TCEQ's daily air quality forecasts [29], NASA satellite images (SI Figure S3), and GMAO Framework for Live User-Invoked Data (FLUID) forecast for Aerosol Optical Thickness [13]. Additionally, HYSPLIT back trajectories tracked air masses back to western Sahara-Sahel region between 11th August – 14th August 2018 (see SI Figures S4, S5, and S6). The NAAPS model also simulated North African dust in Houston during the same timeframe identified in Table 1 of the main manuscript (SI Figure S7). Aerosol Optical Depth (AOD) when combined with TCEQ predictions and HYSPLIT back trajectories, give us strong, but only qualitative evidence of the presence of desert dust in Houston during the corresponding sampling period.

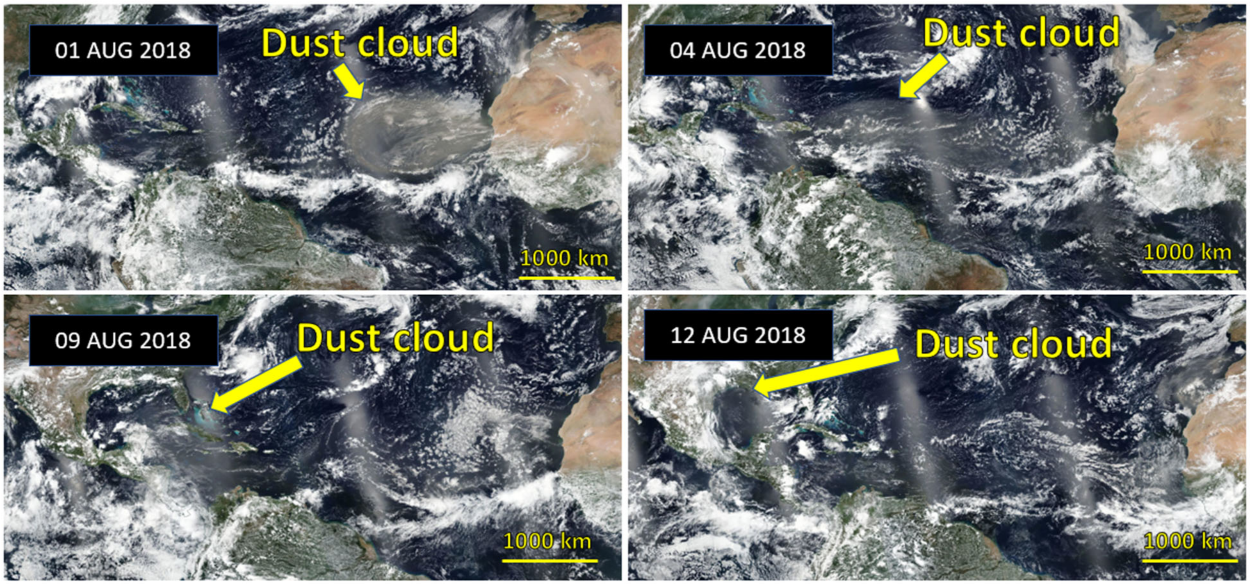

SI Figure S3. Satellite images from NASA Worldview show progression of Saharan dust cloud from North Africa to the Texas coast (https://worldview.earthdata.nasa.gov/).

Summary of HYSPLIT Back Trajectories

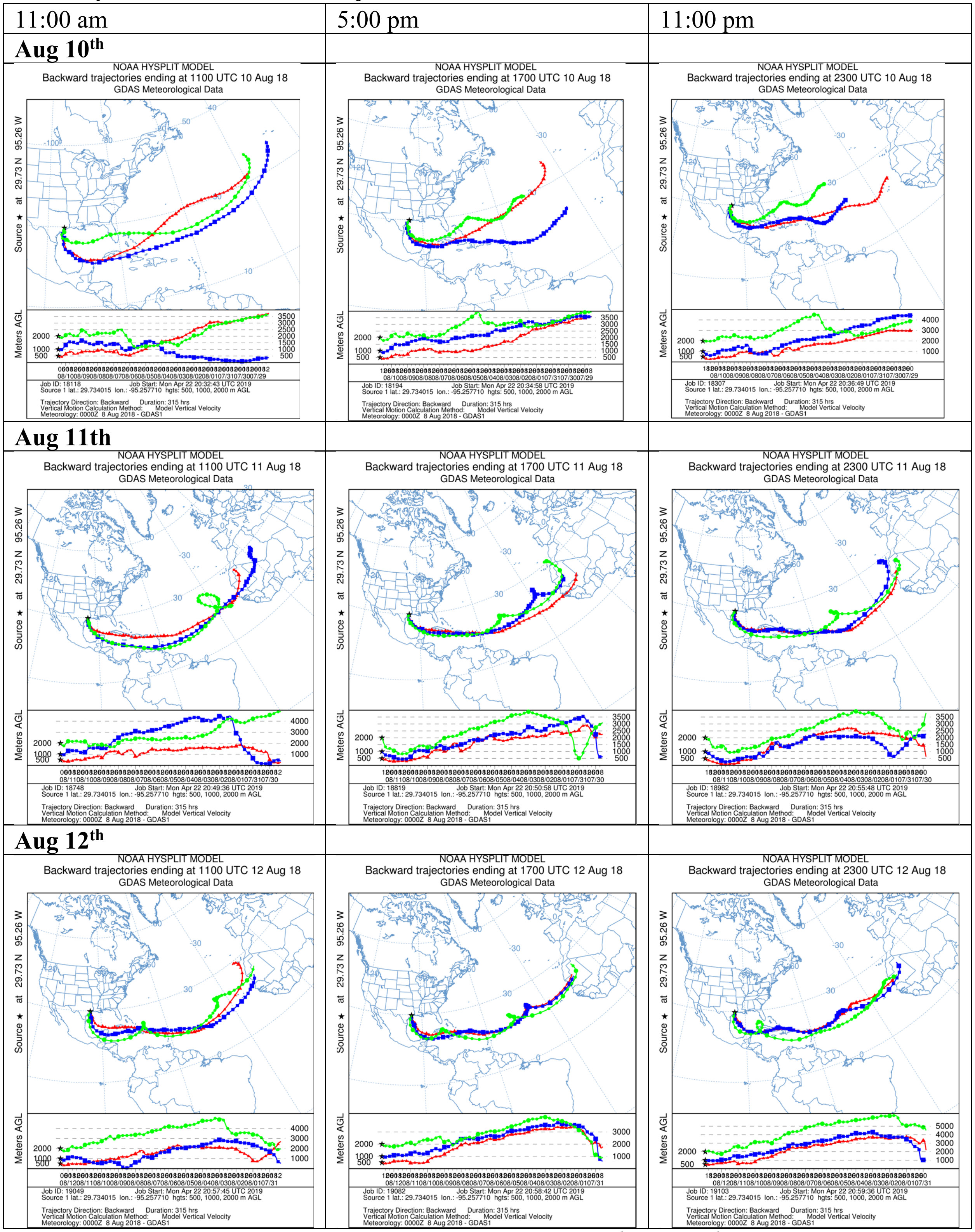

SI Figure S4. HYSPLIT back trajectories for Houston air masses from 10th Aug to 12th Aug, 2018

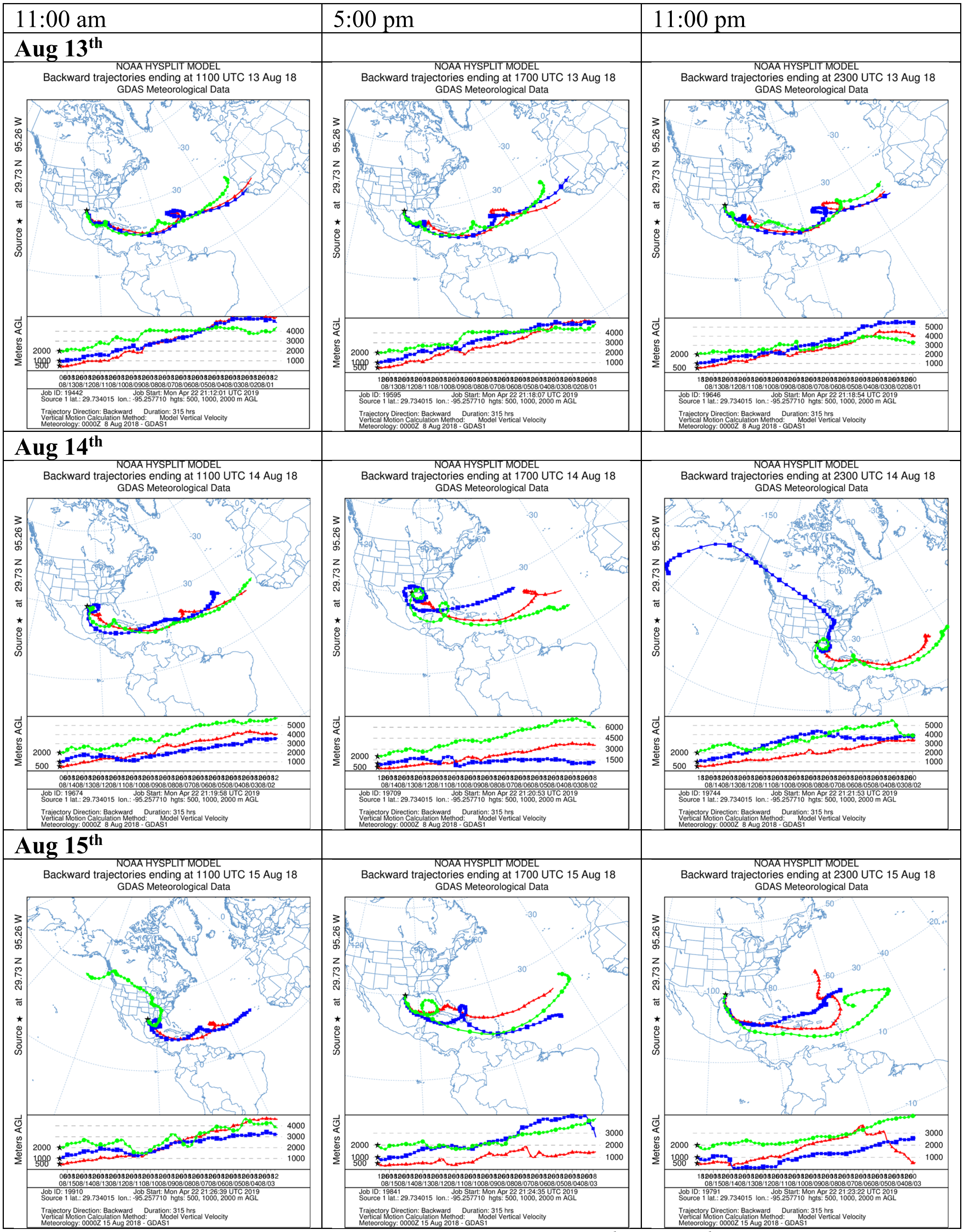

SI Figure S5. HYSPLIT back trajectories for Houston air masses from 13th Aug to 15th Aug, 2018

11:00 am	5:00 pm	11:00 pm

Aug 16th

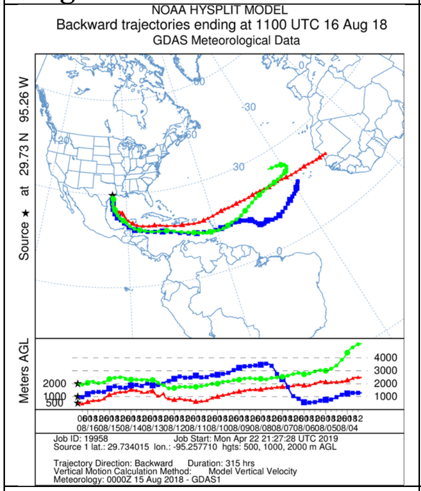

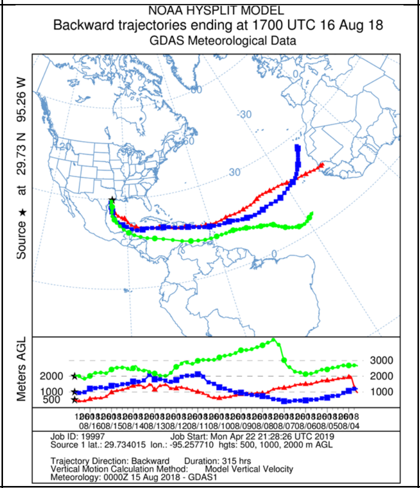

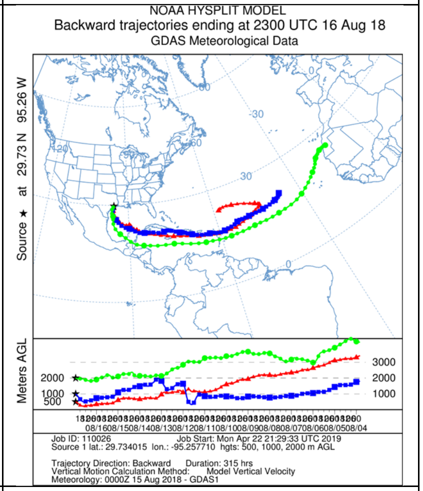

Aug 17th

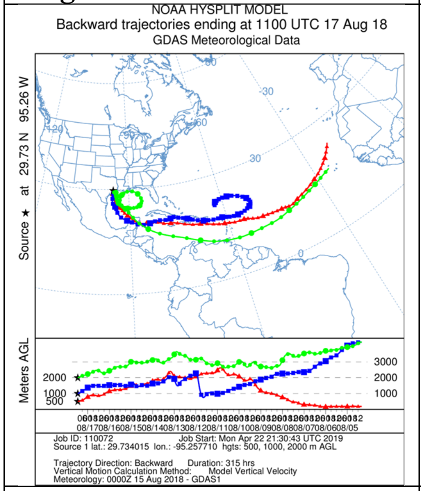

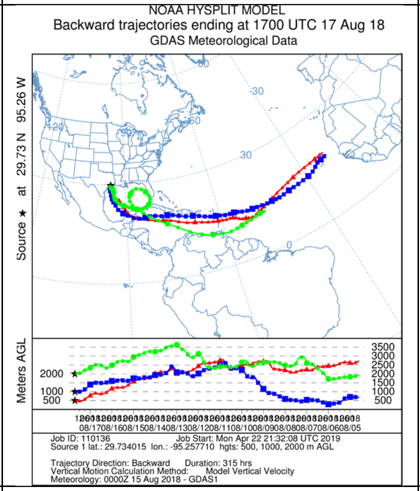

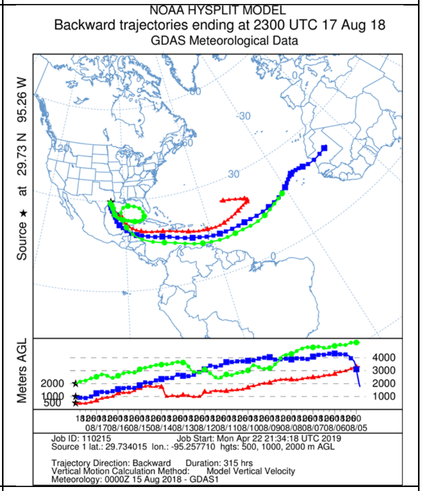

Aug 18th

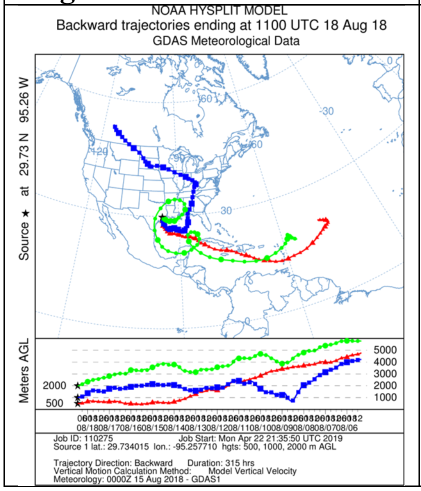

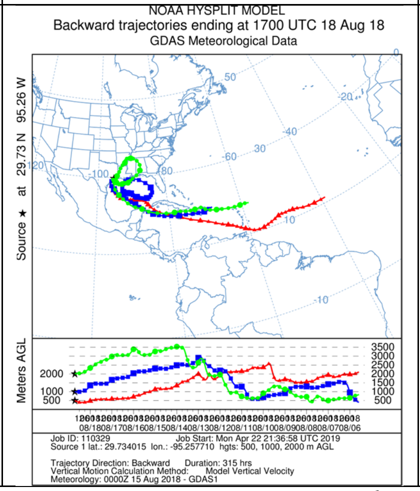

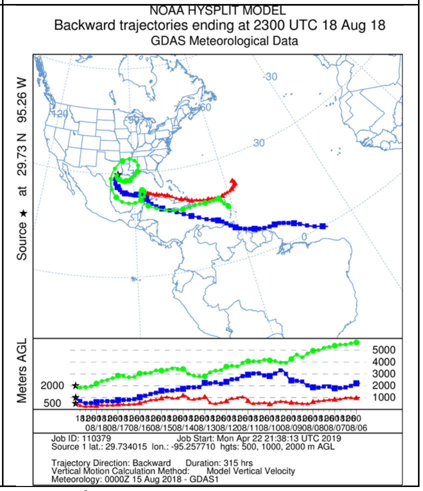

SI Figure S6. HYSPLIT back trajectories for Houston air masses from 16th Aug to 18th Aug, 2018

Note the following parameters used in generating these back trajectories:

- Number of trajectories tracked – 3
- Height(s) of trajectory destination point – 500, 1000, 2000 meters
- Duration of each trajectory – 315 hours (maximum allowed by the model)
- Number of Trajectory Starting Locations – 1
- In the HYSPLIT air back trajectory model, the air parcel itself is the tracer and its position is modelled using meteorological parameters.
- The Global Data Assimilation System (GDAS) model was employed to create back trajectories, using the following meteorological parameters:
- The wind speed and direction: These are the main variables that determine the horizontal movement of the air parcel along the trajectory. They are interpolated from the GDAS grid to the location of the air parcel at each time step.
- The temperature and humidity: These are the variables that affect the density and buoyancy of the air parcel, which in turn influence its vertical movement. They are also interpolated from the GDAS grid to the location of the air parcel at each time step.
- The pressure and geopotential height: These are the variables that define the vertical coordinate system of the GDAS grid. They are used to calculate the height of the air parcel above sea level or ground level, depending on the chosen vertical motion option. In our trajectories the default (recommended) option was chosen.
- The omega field: This is a variable that represents the vertical pressure velocity. It is used to calculate the vertical movement of the air parcel when the default vertical motion option is selected. It is also interpolated from the GDAS grid to the location of the air parcel at each time step.

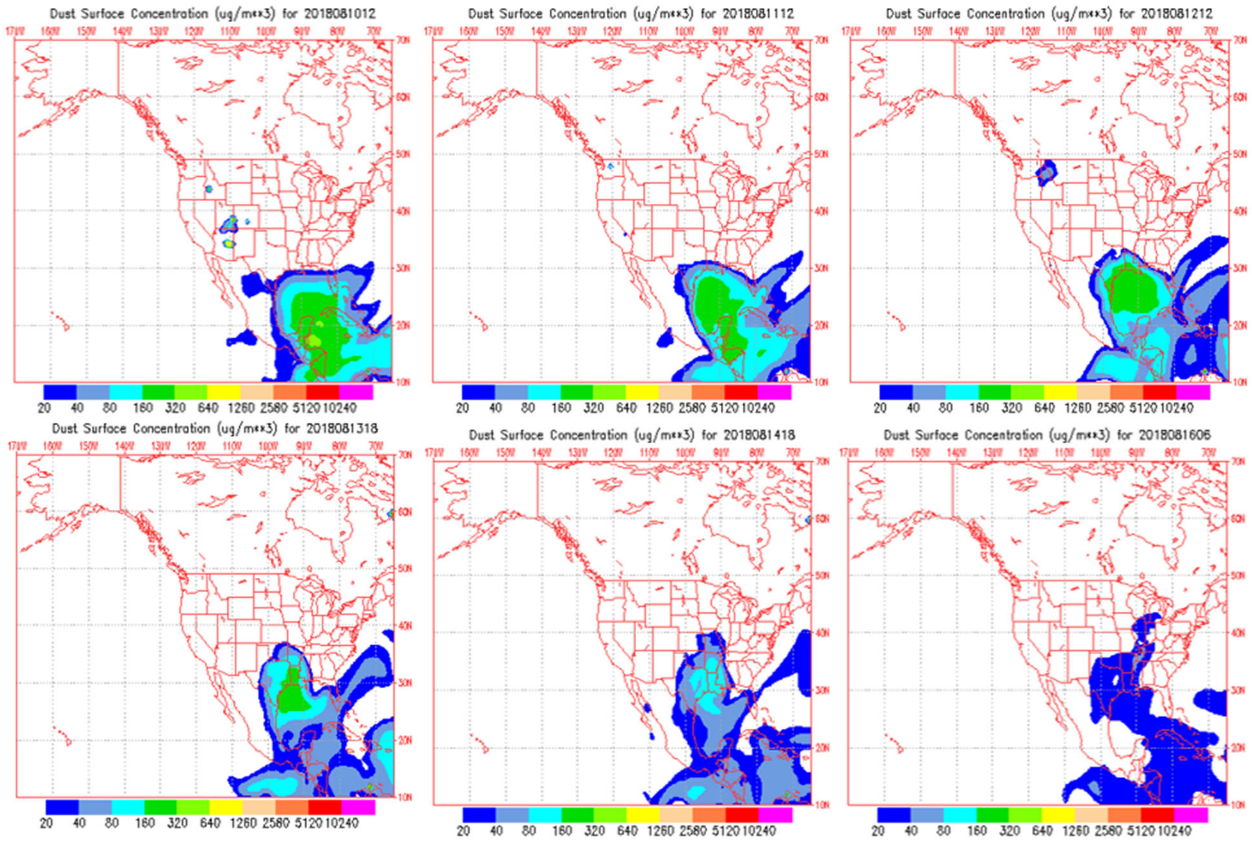

SI Figure S7. United States Navy's NAAPS aerosol model simulated dust mass over Houston from 10th Aug to 16th Aug 2018. Simulations for 11th Aug to 14th Aug depict Saharan dust clouds.

Lingering long-range transported dust at low concentrations cannot be visualized (let alone quantified) by satellite observations for which reason we performed elemental analysis. This reiterates the importance of dust's elemental speciation for source apportionment. Details are given next.

Section S8. Elemental concentrations

SI Figure S8 summarizes elemental concentrations in PM_{10} over the entire study duration. Crustal elements (e.g., Na, Mg, Al, Si, Ca, Ti, and Fe) dominated as expected from aeolian resuspension of desert dust being the important aerosol source. Importantly, rare earth elements (REEs) were consistently detected, which exhibited abundances following the Oddo–Harkins rule with even atomic numbered REEs being more abundant than their immediate neighbors with odd atomic numbers except for samarium anomalies (i.e., La < Ce > Pr < Nd > Sm > Eu < Gd > Tb < Dy > Ho < Er > Tm < Yb >

Lu). Numerous main group (e.g., Ga, As, Sn, Sb, and Pb) and transition metals (V, Cr, Co, Ni, Cu, Zn, Mo, and Cd), which are typical of anthropogenic activity were also detected.

As seen, in **SI Figure S8**, elemental concentrations varied over nearly 7-orders of magnitude e.g., from 2 pg/m^3 for thulium and lutetium in samples S1 and S9 to 18 μg/m^3 for silicon in sample S5. The large variability in elemental composition is attributed to the inherently high variability associated with local PM sources in the Houston area [3, 4, 19, 30-32].

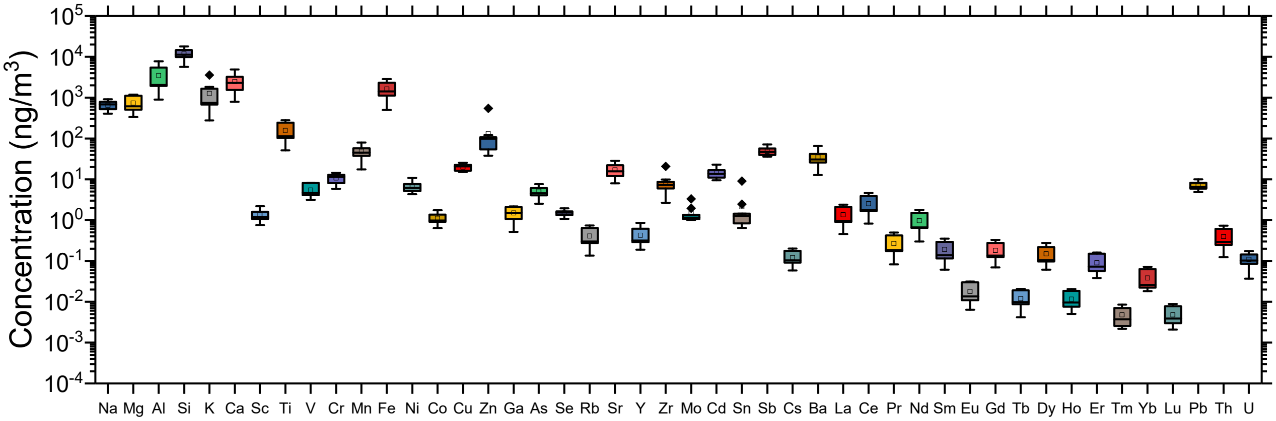

SI Figure S8. Box plot of elemental concentrations in PM_{10} over the study duration. The y-axis is in logarithmic scale to accommodate the 7 orders of magnitude differences in concentrations of individual elements. The box encompasses the 25% and 75% percentiles, the whiskers span 1.5 times the interquartile range, and outliers are shown as diamonds. Inside each box, the horizontal line is the median value and the hollow square symbol (□) is the average value. Each box summarizes a total of 9 daily measurements made between August 10 – 19, 2018. Individual boxes have been arbitrarily colored to help with distinguishing between neighboring boxplots and colors may repeat due to the number of elements measured (46) exceeding colors available.

SI Table S2 summarizes elemental ratios serving as source signatures to track the emissions of petroleum refining catalytic cracking units (La to other light lanthanoids – Ce, Pr, Nd, and Sm), oil combustion activities (La/V), and major crustal metals (Ti, Fe, and Al) during the sampling campaign. The same ratios for African dust measured by us in Barbados [5] and average crustal values [26] are also included for comparison. Primary particles emitted from uncontaminated rocks, soils, and minerals exhibit La depletion relative to Ce and abundances given by the Oddo-Harkin rule [26]. The corresponding La to light lanthanoids ratios (La/Ce, La/Pr, La/Nd, and La/Sm) overlapped closely with the aerosol values

measured throughout the sampling campaign as did other major elemental ratios (e.g., Ti/Al, Fe/Al, and Ti/Fe). These results demonstrate the crustal nature of dust during the entire sampling period.

SI Table S2. Elemental ratios to validate the overall crustal (North African) origin of the air mass. High dust days – S2, S3, S4, S5

Ratio	Average	Ratio during high dust days	Ratio on other days	Crustal ratio [26]	Barbados African dust [5]
La/Ce	0.55±0.05	0.53±0.01	0.57±0.06	0.49	0.48±0.01
La/Pr	5.26±0.56	4.91±0.16	5.54±0.63	4.37	4.2±0.03
La/Nd	1.46±0.19	1.35±0.04	1.54±0.22	1.15	1.1±0.01
La/Sm	7.27±0.64	6.91±0.21	7.55±0.75	6.60	5.9±0.10
La/V	0.24±0.05	0.27±0.02	0.21±0.05	0.320	0.38±0.03
Ti/Al	0.050±0.010	0.044±0.009	0.054±0.003	0.047	0.050±0.002
Fe/Al	0.54±0.11	0.43±0.06	0.62±0.06	0.48	0.51±0.02
Ti/Fe	0.094±0.013	0.100±0.010	0.090±0.010	0.098	0.110±0.005

Oil combustion aerosols are enriched in V relative to La resulting in low La/V ratios such as 0.045±0.015 for oil-fired power plants [33] and 0.01-0.11 for shipping [34-36]. This is an important industrial and economic activity in Houston, Texas as shown by us in previous publications. Even though North African dust dominated aerosols during this study, the influence of oil combustion is manifested by a substantially lower La/V ratio in Houston aerosols (0.24) compared with the Saharan value (0.38), i.e., a 37% reduction as shown above in SI Table S2.

SI Table S2a. Summary of elemental concentrations on high dust and low dust days. All values are in ng/m^3.

	High dust days					Low dust days			
Element	Concentration	Std dev	Min	Max		Concentration	Std dev	Max	Min
Na	348	108	201	456		266	53	201	344
Mg	443	162	281	593		248	60	168	304
Al	2480	1243	1380	3903		853	240	443	1018
Si	6551	2623	3688	9030		4746	1525	2827	6673
K	923	584	463	1771		301	93	137	371
Ca	1215	1011	389	2456		1159	384	725	1668
Sc	0.73	0.15	0.56	0.89		0.51	0.08	0.38	0.60
Ti	101	31	70	135		45.9	12.6	25.6	56.6
V	3.15	1.08	2.06	4.10		1.95	0.36	1.57	2.35
Cr	5.26	2.19	2.87	7.26		4.84	1.51	2.90	6.47
Mn	27.8	12.9	14.6	39.7		18.7	6.4	8.7	25.8
Fe	1023	404	590	1426		533	170	249	705
Ni	2.10	0.47	1.46	2.56		3.94	0.999	3.05	5.45
Co	0.59	0.14	0.44	0.73		0.428	0.07	0.32	0.49
Cu	8.90	3.50	4.29	11.85		9.82	2.12	7.55	12.68
Zn	34.7	19.5	10.9	52.5		87	104	19	273
Ga	0.76	0.33	0.44	1.04		0.65	0.31	0.26	1.09
As	2.47	0.67	1.77	3.05		2.23	0.96	1.25	3.80
Se	0.51	0.24	0.22	0.79		0.37	0.34	0.02	0.83
Rb	0.27	0.10	0.18	0.37		0.12	0.03	0.07	0.15
Sr	9.68	4.54	5.60	14.18		6.50	1.83	3.99	8.57
Y	0.25	0.08	0.16	0.33		0.14	0.03	0.09	0.17
Zr	4.42	0.88	3.21	5.21		2.90	1.14	1.33	4.28
Mo	0.52	0.14	0.33	0.68		0.84	0.50	0.50	1.65
Cd	5.68	0.69	4.90	6.54		7.27	1.63	4.73	8.96

Sn	0.70	0.38	0.32	1.22
Sb	21.9	10.1	11.9	36.0
Cs	0.07	0.03	0.05	0.10
Ba	15.1	7.8	7.7	23.2
La	0.86	0.32	0.59	1.20
Ce	1.627	0.595	1.113	2.303
Pr	0.175	0.065	0.117	0.249
Nd	0.633	0.223	0.441	0.885
Sm	0.124	0.045	0.084	0.176
Eu	0.012	0.004	0.008	0.016
Gd	0.114	0.042	0.074	0.161
Tb	0.007	0.003	0.005	0.01
Dy	0.100	0.043	0.056	0.138
Ho	0.007	0.003	0.004	0.01
Er	0.058	0.022	0.036	0.077
Tm	0.003	0.001	0.002	0.004
Yb	0.025	0.011	0.016	0.036
Lu	0.003	0.001	0.002	0.004
Pb	3.28	1.60	1.49	4.98
Th	0.26	0.10	0.17	0.37
U	0.06	0.03	0.04	0.09

1.28	1.82	0.32	4.53
23.5	5.2	17.9	28.5
0.04	0.01	0.03	0.05
17.5	9.9	6.4	32.6
0.411	0.104	0.227	0.476
0.729	0.198	0.409	0.885
0.075	0.021	0.041	0.092
0.272	0.08	0.15	0.332
0.055	0.015	0.031	0.069
0.005	0.001	0.003	0.007
0.055	0.014	0.035	0.068
0.004	0.001	0.002	0.005
0.044	0.010	0.030	0.052
0.004	0.001	0.003	0.005
0.027	0.007	0.019	0.036
0.002	0.001	0.001	0.002
0.011	0.002	0.009	0.013
0.002	0.001	0.001	0.002
3.30	0.59	2.57	4.12
0.12	0.03	0.06	0.15
0.04	0.01	0.02	0.05

Section S9. Elemental Enrichment Factors

Because resuspended dust from the Sahara-Sahel region originates from surface soils [24, 37-39], it is abundant in mineral metals such as Ca, Mg, Al, Fe, Na, K, and Ti [40]. Consequently, African dust intrusion in highly polluted urban atmospheres such as Houston should dilute abundances of anthropogenic transition metals (e.g., Cr, Ni, Cu, Zn, Mo, Cd, W, and Ba) and post-transitional metal(loid)s (e.g., As, Sn, Sb, and Pb) [6, 41]. This hypothesis was evaluated by analyzing the temporal evolution of Enrichment Factors (EFs) of these elements referenced to Ti in the upper continental crust [42] as depicted in SI Figure S9 and the formula is given below:

$$Enrichment\ factor\ (X) = \frac{\left(\frac{X_{sample}}{Ti_{sample}}\right)}{\left(\frac{X_{UCC}}{Ti_{UCC}}\right)}$$

These metals were significantly enriched with EFs ranging between ~20 (for Cr, Ni, W, and Pb) to 5,000 (for Cd) on August 10 as expected from their considerable local industrial and vehicular emissions [6, 8, 16, 41, 43]. EFs of these elements decreased substantially during August 11-14, 2018, which is consistent with incursion of massive quantities of crustal elements. Zinc showed the highest percentage reduction (84%), and arsenic showed the lowest percentage reduction (51%). Fractional decreases for all other anthropogenic metals were between the arsenic minimum and zinc maximum as shown in SI Figure S9. EFs gradually climbed back up during August 15-19. The temporal behavior of numerous anthropogenic elements' EFs (i.e., initially high, then decreasing, and then increasing) is exactly coincident with the $PM_{10}/PM_{2.5}$ mass ratio profile (SI Figure S2) and Saharan-Sahelian dust visualized by satellites and synoptic-scale models (SI Section S7). This provides further evidence for North African dust's presence in Houston during August 11-14, 2018.

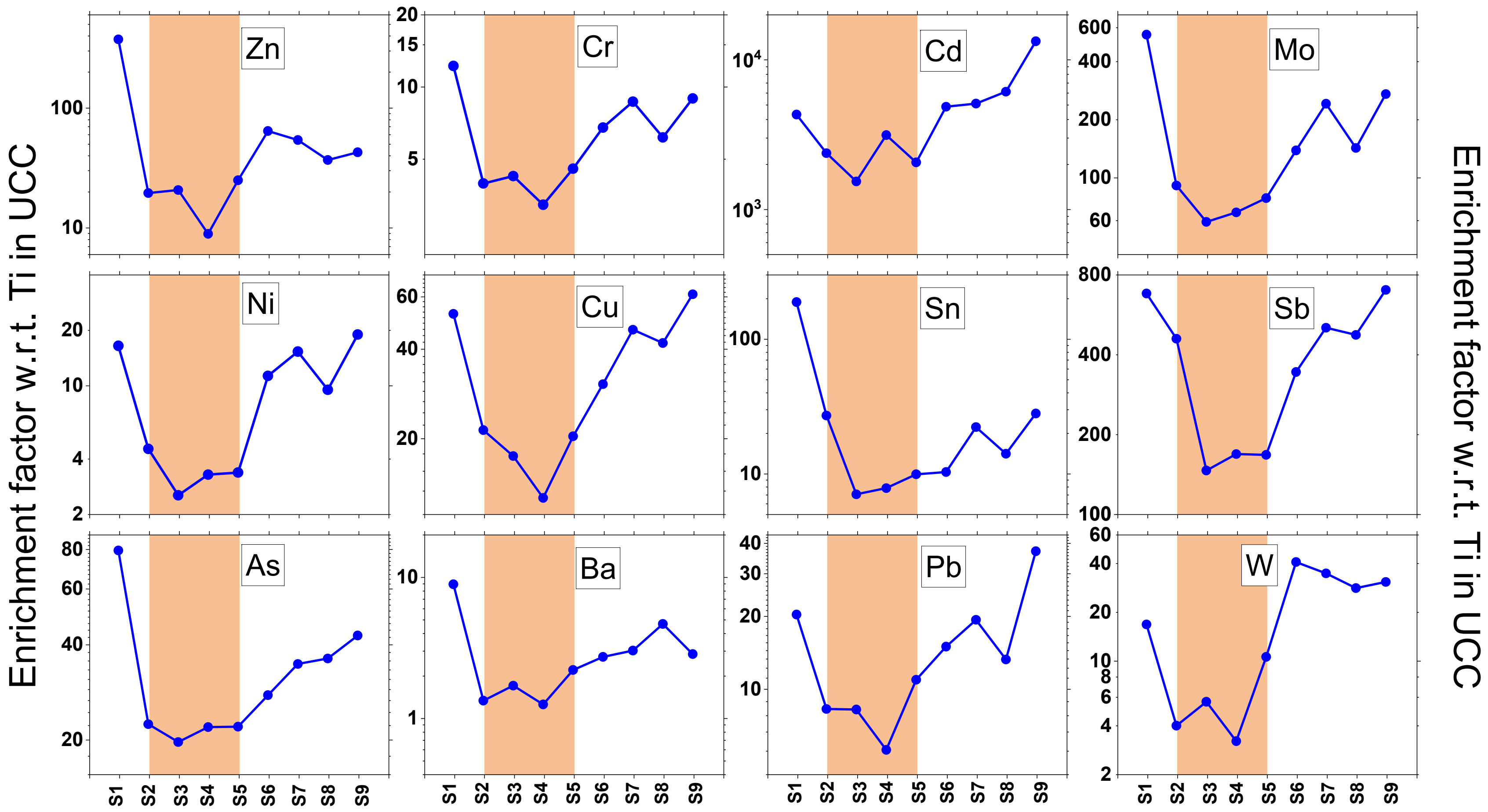

SI Figure S9. Reduction in enrichment factors of anthropogenic elements referenced to titanium in the upper continental crust from 11 Aug to 14 Aug 2018 indicating crustal dust intrusion during the same period into Houston atmosphere. Note y-axes are in log-scale because of substantial variations in EFs.

Note that the enrichment factors of all metals did not evolve similarly in magnitude, which is attributed to variations in their local emissions and reported by us earlier [2-4, 7, 8, 15, 19]. This is a recurring theme in Houston where many aging refineries and industries often experience process upsets and release (sometimes large quantities of) metals to the local atmosphere. For example, there are differences in enrichment factor patterns of As Cu, and Zn, which indicates different sources might be contributing to these sources. Cu and Zn are released with industrial emissions and non-tailpipe emissions of motor vehicles while As is emitted via fuel combustion and high temperature industrial operations.

Additional subtle features in elemental behavior in SI Figure S9 can be interpreted. As observed, the enrichment factors of several anthropogenic metals (e.g., Cr, Mo, Ni, Cu, Sn, Sb, and Pb) did not rise monotonically after the peak episode (samples S2-S5 background colored in light brown). In particular, the enrichment factor of these metals in the penultimate sample (S8) dropped before rising again in sample S9. This is consistent with CMB apportionment of higher North African dust in sample S8 compared with its neighbors (samples S7 and S9). This is also shown in the ternary plot in the main manuscript (Figure 1b) where sample S8 is closer to the upper continental crust (UCC) centroid than sample S6, which is chronologically closer to the peak episode. We have observed similar behavior in other African dust episode that we have monitored using light rare earths when petroleum refining aerosols were dominant in Houston [2, 3, 6, 25]. In this sampling campaign, refining was not a major contributor as explained below in SI Section S11 but high temperature (combustion) operations demarcated industrial and crustal emissions. Hence, mixing between locally emitted anthropogenic aerosols and long-range crustal dust was evidenced clearly via elemental analysis demonstrating its value for quantitative source apportionment (especially delineating foreign aerosols that are not covered under the United States Environmental Protection Agency's Clean Air Act).

Section S10. Aluminium Enrichment as Further Evidence of Saharan Dust

The higher aluminum content of African dust [3, 5, 44] increases the Al/Ca, Al/Fe, and Al/Si ratios when compared to other crustal sources including local soil in Houston. Al/Si for African dust lies between 1.1

and 3.3 [3, 5] in contrast to local Houston soil that has Al/Si between 0.3 to 0.7 [6, 41]. Similarly, Al/Ca and Al/Fe for North African dust lie between 1.1 – 2.5 and 1.8 – 2.2, respectively, whereas the same ratios for Houston soil are substantially lower ranging between 0.14 – 0.50 and 1.3 – 0.7, respectively [6]. The contrast in Al/Ca is greater than other ratios because of Ca enrichment in addition to Al depletion in Houston soil as compared to North African dust and average upper continental crust (UCC). Enrichment of Ca in local soil has been identified in our earlier work [6, 41] and has been attributed to Ca-rich sources in the Ship Channel area (near the sampling site for this manuscript). Increase of the Al/Ca, Al/Fe, Al/Si ratios on high dust days further validates that they were diluted with African dust on episodic days rather than local soil. The Ca-Al-Ti ternary plot (**SI Figure S11**) explains aluminum enrichment by showing Houston PM_{10} moving closer to African dust and average UCC (centroid) on episodic days compared to regular days that lie closer to local soil (towards Ca apex). Specifically focusing on aluminum revealed that the crustal mass dilution in samples S2-S5 primarily arose from African dust as opposed to local soil.

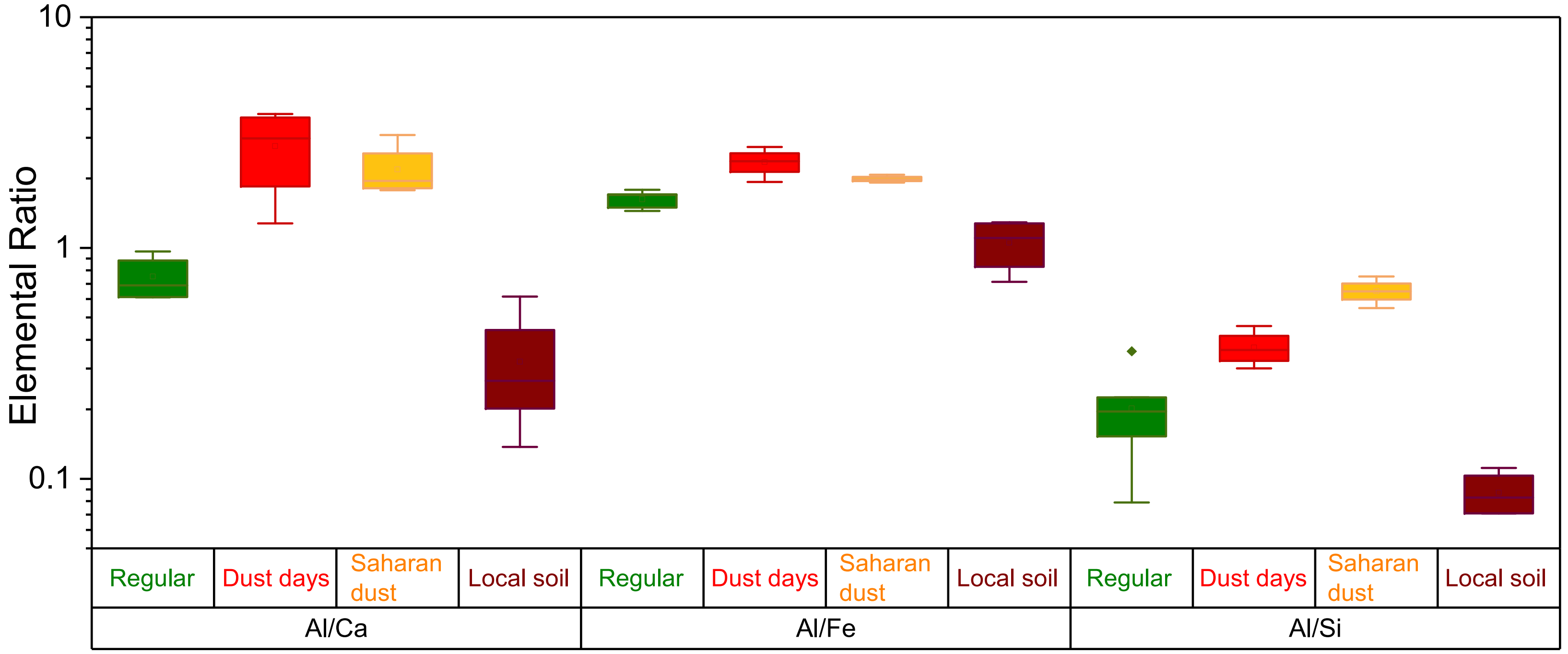

SI Figure S10. *Comparison of crustal element ratios during episodic days, regular days, Saharan dust [3, 5], and Houston soil [6, 41]. Higher ratios of Ca, Fe, and Si with Al indicates Saharan dust in Houston atmosphere during alleged dust episodes.*

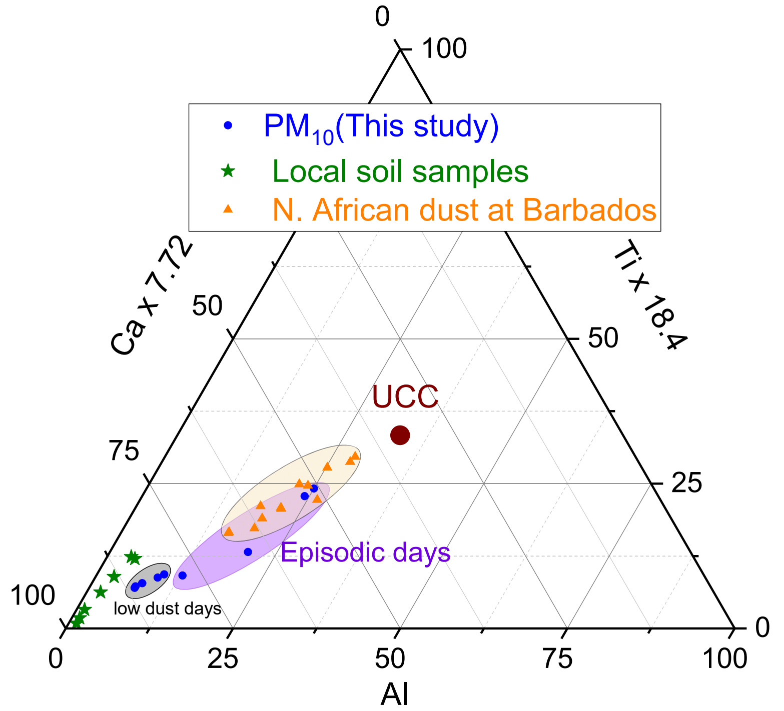

SI Figure S11. Al-Ti-Ca Ternary to differentiate local dust and African dust. Episodic days move towards the upper continental crust profile (centroid) while regular days samples move towards Ca-rich profile of Houston soil. The North African dust and local soil composition were taken from our previous studies [3, 5, 6, 41].

Section S11. Rare earths were dominated by crustal matter during entire study period exhibiting no influence from petroleum refining

Lanthanum ratios (La/Ce, La/Nd, and La/Sm) are useful to trace relative contributions of rare earths in areas under the influence of petroleum refining and mineral dust [8, 27, 45, 46]. The La/Ce ratio remained unchanged over the entire study and was approx. 0.5 overlapping with the crustal ratio reflecting crustal dominance for rare earths throughout the study duration. Our earlier measurements in Houston have revealed that on days dominated by refinery emissions, the La/Ce was significantly greater 1, i.e., of anthropogenic origins [2, 4, 7, 45, 46]. Since all the signature rare earth ratios measured in PM_{10} overlapped with their corresponding upper continental crust values, refinery emissions were negligible and crustal matter were the dominant source of rare earths. Also, La-Ce-Sm ternary plots (SI Figure S12) showed that all PM_{10} samples clustered near the profile of the upper continental crust (at the centroid) and local soil validating the crustal nature of all lanthanides during the entire course of this investigation.

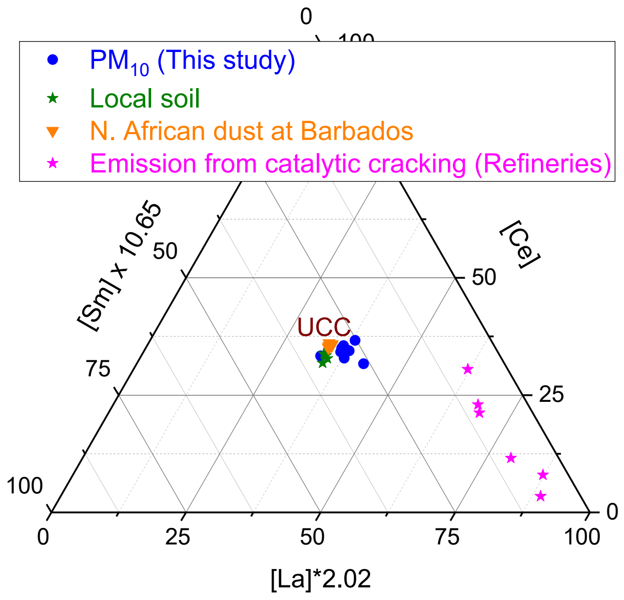

SI Figure S12. La-Ce-Sm ternary plot showed all samples grouping close to the upper continental crust and local soil profiles and plotting away from the La apex revealing that they were not emitted from anthropogenic sources such as petroleum refining.

Section S12. Validating mineral mass with Reconstructed Measured Mineral Matter (RMMM) mass.

Mineral matter is mostly constituted by oxides of Si, Al, Fe, Ca, Na, Mg, and Ti [42]. The Reconstructed Measured Mineral Matter (RMMM) is a parameter often used to estimate total mineral mass (North African dust + local soil + concrete dust in our case) by converting measured amount of the above elements into respective oxide mass [2, 3, 5, 47, 48]: $RMMM = 1.89 \times Al + 2.14 \times Si + 1.40 \times Ca + 1.67 \times Ti + 1.67 \times Mg + 1.43 \times Fe + 1.34 \times \mathrm{Na}$

In this study, the RMMM correlated with a slope of 1.04 ± 0.05 ($R^2 = 0.98$) with PM_{10} apportioned to total mineral mass (SI Figure S13), quantitatively validating our results of the total apportionment of PM to the three mineral sources (i.e., Saharan dust, local soil, and cementitious materials arising from construction activities).

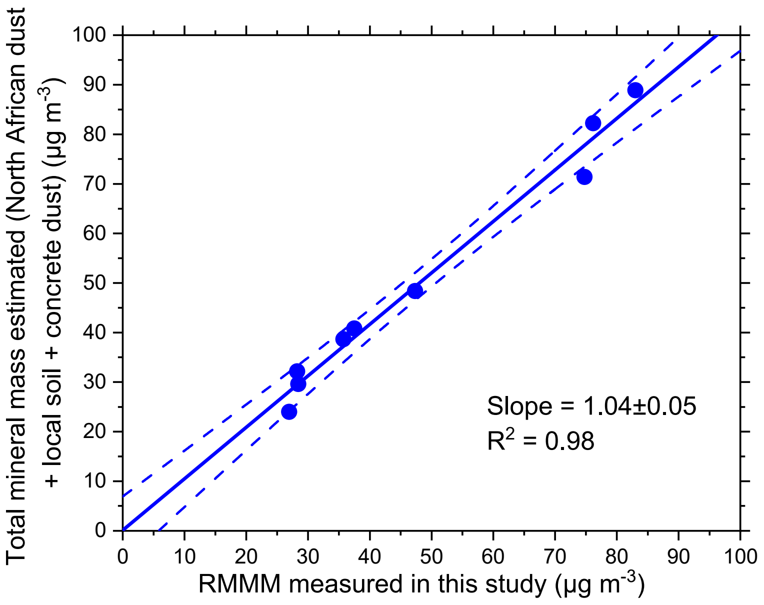

SI Figure S13. Reconstructed Measured Mineral Mass calculated in this study corelates with estimated total mineral mass with a slope ~1, validating our estimates of mineral mass with Chemical mass Balance (CMB) model.

Concentrations of major crustal elements (Al, Si, Ca, Mg, Fe, Na, and Ti) were converted to their respective oxides to calculate the Reconstructed Measured Mineral Material (RMMM) mass.[2, 3, 25] This equaled the total apportioned mass of mineral dust (North African + local soil + concrete) validating the robustness of our CMB estimates (slope 1.04±0.05 in SI Figure S13). Hence, the Reconstructed Measured Mineral Material (RMMM) mass[2, 3, 25] accurately estimated the total apportioned mineral dust mass (North African + local soil + concrete) validating CMB estimates. RMMM results coupled with La-Na-V behavior in **Figure 1b** of the main manuscript demonstrate the ability of elemental measurements to sensitively track ground-level behavior of African dust, a capability that is necessary to evaluate human exposure. Cumulatively, these results provide necessary information to test our first hypothesis and potentially link metagenomic data with African dust (and aerosol sources), which is pursued in the following sections.

Section S13. Validating CMB estimates of Saharan dust with Al/Si ratio.

Saharan dust is known to be more aluminous [3, 5, 44] and local soil in Houston is more siliceous [6, 41] in composition. To verify accuracy in differentiating local dust and Saharan dust by the chemical mass balance model, we used measured Al/Si ratio in PM samples and compared it with our apportionment

results. Peaks in Al/Si corresponded with high Saharan dust to local soil ratio (see Figure 1), validating estimates by CMB in this study. Although Ca/Al was demonstrated to be easily distinguishable parameter between Saharan dust and local soil, it has several interferences from other Ca-rich sources (like concrete dust and cement manufacturing plants) as reported in our earlier studies in Houston [3, 6], therefore, was not used for validation of Saharan dust apportionment.

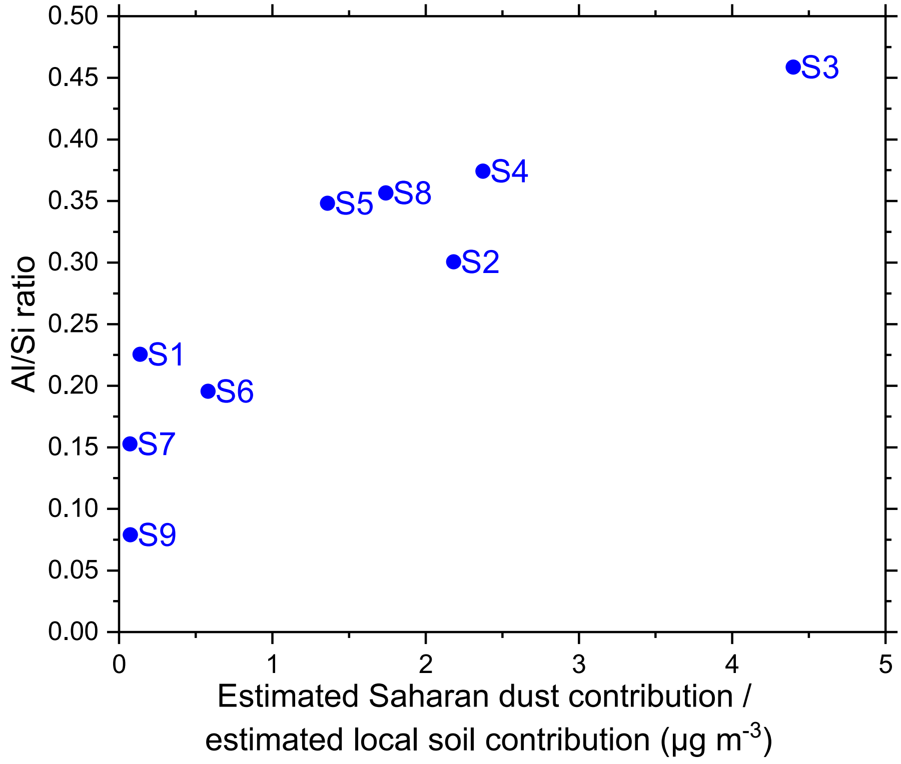

SI Figure S14. Al/Si, which can distinguish between crustal sources was synchronous with apportioned Saharan dust to local soil mass ratios validating CMB calculations of both crustal sources (North African and Houston soil).

SI Table S3. 24-hour averages of several environmental variables at sampling location Clinton Drive

	Aug 10-11	Aug 11-12	Aug 12-13	Aug 13-14	Aug 14-15	Aug 15-16	Aug 16-17	Aug 17-18	Aug 18-19
**CO (ppmv)**	0.25	0.12	0.12	0.13	0.13	0.15	0.16	0.13	0.1
**Oxides of Nitrogen (ppbv)**	17.68	9.69	10.58	18.79	15.79	16.88	17.35	11.7	6.19
**Ozone (ppbv)**	13.71	19.35	13.96	12.54	16.08	13.46	14.92	16.38	18.48
**Solar Radiation (Ly/min)**	0.24	0.27	0.34	0.41	0.39	0.33	0.32	0.35	0.38
**Temperature (°F)**	83.63	86.28	89.12	89.05	89.52	88.68	89.03	89.77	89.87
**Relative Humidity (%)**	84.3	78.43	70.24	68.26	69.01	74.71	74.31	70.23	70.65
**Wind Speed (mph)**	3.88	6.82	9.59	8.72	8.99	7.92	6.8	7	8.13

Section S14. Methodology for nucleic acid extraction, gene sequencing, statistical analysis, and generalized dissimilarity modeling and results

Nucleic acids were extracted from the PM_{10} filter samples using bead-beating and purification with centrifugal filters. Filters were cut in half, folded with sterile forceps, and placed into 2 mL bead beating tubes (National Scientific supply, BC20NA-PS), along with 200 mg of ≤ 106 μm diameter glass beads (Sigma, G-4649), 200 mg of 425-600 μm glass beads (Sigma, G-8772), and 650 μL of sodium phosphate buffer prepared aseptically by mixing 1.0 μL Tween 20 with 10 ml of 0.1 M sodium phosphate/10 mM EDTA (Teknova, Hollister, CA). The bead beating tube was mechanically disrupted with a mini-bead beater (BioSpec Products, Bartlesville, OK) for 3 min at 3450 oscillations/min and subsequently placed on ice for five minutes. After cooling, tubes were centrifuged at 7000×g for 2 min. 500 μL of supernatant was transferred to a Ultrafree-MC centrifugal filter (0.22 μm pore size, hydrophilic PVDF, 0.5 mL volume, UFC30GV0S) and centrifuged at 10,000×g for 3 min. Following the centrifugal pre-filtration, the filtrate was transferred to an Amicon Ultra-0.5 (100,000 Molecular Weight Cut-Off) centrifugal filter device (YM-100, Millipore) and centrifuged for 3 min at 7000×g. This step concentrated nucleic acids on the filter while allowing inhibitory compounds to pass through. The filter was first washed twice, each time by adding 200 μL of TE buffer to the retentate cup and centrifuging for 3 min at 7000×g. The acronym "TE" refers to a combination of Tris, a common pH buffer, and EDTA (ethylenediaminetetraacetic acid), a chelating agent. After the second wash, 100 μL of the TE buffer was added and centrifuged for 1 min at 7000×g and subsequently in 10 second pulses as needed to recover 100 μL through the filter. Nucleic acids were recovered from the top of the Amicon filter insert by inverting the filter into a fresh tube by centrifugation at 1000×g for 2 min. Final purification of nucleic acids was performed as per manufacturer's protocol using a polyvinylpolypyrrolidone spin column (Spin-IV-HRC, Zymo Research).

The 16S rDNA gene is divided into nine (V1-V9) hypervariable regions, each with its own degree of sequence diversity. More conserved, or unchanging, regions are best suited only for high level taxonomic identification whereas more variable regions can differentiate between genera or species [49, 50] Combining multiple hypervariable regions can increase sequencing resolution. V3-V4 is a commonly

selected combination in bioaerosol research [51-54]. The 18S/ITS rDNA gene is commonly used for taxonomic identification of eukaryotes and has a similar dynamic of conserved to hypervariable regions within it.

We followed the protocol recommended by Swift Biosciences to generate amplicons that covers all variable regions of the 16S rDNA, ITS1, and ITS2 genes in a single primer pool using the Swift Amplicon 16S + ITS kit. The PCR products (obtained following their protocol) were cleaned up using the Agencourt AMPure XP beads (Beckman Coulter Genomics) and the purified amplicon was resuspended in 25 μl TE buffer. The first round of purified amplicon was amplified using the Illumina adaptors specific dual indexed Nextera XT barcoded primers of index 1 (i7) adapter and index 2 (i5) adapter with 15 cycles of amplification followed by clean-up. The amplicons were purified with Agencourt AMPure XP beads, quantified with Qubit (Invitrogen), and verified the amplicon size with Agilent TapeStation 2200 system. The library pool was diluted to obtain a final concentration of 8 pM. The 600 μL of the library pool was loaded on to a MiSeq v2 reagent cartridge (500 cycle v2 kit) and 251 bp paired-end sequencing protocol (2×251 cycles) was performed on MiSeq platform (Illumina).

Raw sequence reads were cleaned and processed using QIIME v1.9.1.[55] Forward and reverse reads were merged, assigned to samples according to barcodes, and then trimmed to remove barcode and primer sequences. Sequences shorter than 200 bp, those containing ambiguous bases, and those with mean quality scores of <20 were discarded. The remaining sequences were then aligned to the reference UCHIME RDP 'Gold' database to identify and remove chimera sequences. Sequence reads were clustered into Operational Taxonomic Units (OTUs) using VSEARCH v.1.9.6 [56] and the SILVA 119 database [57] for 16S and the UNITE database for ITS [58].

OTU is an operational definition of a classification unit (genus, species, grouping, etc.) commonly used in population genetics to facilitate data analysis. In bioinformatics each sequence obtained from sequencing is assumed to be derived from a single species. All the sequences in a sample are classified to obtain information on species and genus. By classification, the sequences are grouped according to their

similarity, and one group is an OTU. For both gene regions, sequences were assigned to OTUs using a 97% threshold, with taxonomic categories parsed up to the species level, and subsequently rarified prior to downstream analyses.

Field and laboratory blanks were processed along with samples S1 – S9 serving as appropriate negative controls. No amplifiable amount of identified/reported DNA were found similar to earlier studies and validating our protocols [59-62].

Statistical analyses

To estimate day-to-day fluctuations in microbial biodiversity, Chao1 and Shannon indices were calculated for each sample using QIIME. Both indices account for species richness and abundance, though infrequently occurring species are more heavily weighted in Chao1. Similarities and differences among samples were visualized by conducting PCoA (Principal Coordinate Analysis) using a Bray-Curtis distance matrix (beta diversity) derived from OTU relative abundance data in the Python package "skbio.ordination." [63]

To test the hypothesis that the abundance of OTUs fluctuated across sampling days in conjunction with changes in the source and elemental composition of aerosol pollution, Pearson's correlations were used. To remove outlier effects, only OTUs with an abundance of at least 5 individuals in at least 7 of 9 sample days were considered, and only pollution sources and elements with substantial day-to-day variation were considered. OTUs that have fewer than 5 individuals across sequence days were extremely rare compared to the other OTUs. Hence, these were removed prior to statistical analyses to minimize spurious correlations. A subset of 27 elements (Na, Mg, Al, Si, K, Ca, Ti, V, Cr, Mn, Fe, Ni, Co, Cu, Zn, As, Se, Sr, Zr, Mo, Cd, Sn, Sb, Ba, La, Ce, and Pb) was employed for this aspect of the research based on their association with the four dominant apportioned sources (North African dust, local soil/road dust, construction activity (or cement), and vehicular emissions). Using these reduced datasets, the daily log-transformed abundance of each OTU was regressed against source and elemental concentrations.

Note that having more independent variables than the number of sources should increase the

number of significant correlations, all else being equal. However, we don't believe that this is a bias in our approach but argue instead that this pattern points to the increased value of using elemental data rather than source data alone. This is because, while the cement/construction industry releases more calcium than the other sources considered, it also co-emits many other elements. Likewise, other aerosol sources will also emit calcium (e.g., local soil/road dust, sea salt, North African dust, industrial emissions, etc.). We show the complicated nature of collinearity between major sources and elements in SI Figure S22. Note that we have also addressed issues related to collinearity and the use of isotopic analysis to better resolve African dust in Houston in our recent publications [6, 25], but is outside the scope of this manuscript.

Generalized dissimilarity modeling

Generalized dissimilarity modeling (GDM) was implemented in the R package gdm v1.5.0-9.1[64] to determine the mixed effects of fluctuating pollution sources and elemental compositions on bacterial and fungal beta diversity across sampling days [65, 66] Briefly, GDM uses maximum likelihood and flexible I-splines to transform the independent variables and estimate the relationship between beta diversity and environmental fluctuation, accommodating the curvilinearity of both dependent and independent variables [67]. Separate GDMs were developed for bacteria and fungi, with predictor variables consisting of either estimated aerosol source contributions or elemental concentrations. In each case, the proportion of deviance explained by the model and the sum of I-spline coefficients for each variable (i.e., the magnitude and importance of that variable in explaining beta diversity) were recorded.

An unweighted pair group method with an arithmetic mean (UPGMA) tree was generated in QIIME separately for both 16s and ITS sequences to visualize and analyze phylogenetic patterns of OTU abundance. To test whether daily OTU abundance was phylogenetically autocorrelated – i.e., whether closely related OTUs exhibit more similarities in their abundances than expected than if observed abundances were random – Pagel's λ was calculated on 999 Monte Carlo simulations using the 'phylosig' function in the R package phytools [68]. This analysis tested the hypothesis that evolutionarily related OTUs exhibited similar abundances across days, which would be expected if the ecological tolerances of

OTUs in response to pollution source and composition were evolutionarily conserved. The R package ggtree [69] was used to visualize phylogenetic patterns of OTU richness, abundance, and significant correlations to pollution sources and elemental predictors.

Quality control

Sequencing Data Quality Optimization

Sequencing errors might occur in high-throughput sequencing, and it is common that bases toward the end of the sequence reads have lower than average quality. In order to obtain higher quality and more accurate bioinformatic analysis results, it is necessary to optimize the raw data of the sequencing to obtain higher quality and more accurate bioinformatics analysis results. Analysis software: Cutadapt(v1.9.1), Vsearch(1.9.6), and Qiime(1.9.1).

Steps and parameters for optimization:

(1) The two sequences of each read pair were merged according to overlapping sequences. The read merge was deemed to be successful only if the overlapping sequence is least 20bp long. After merging, undetermined bases (N) were removed from the resulting sequence.

(2) Primer and adapter sequences were removed. Then the 5' and 3' bases with Q score lower than 20 were also removed. The resulting sequences with length > 200bp would pass this step of processing.

(3) The sequences obtained were then aligned to UCHIME 'Gold' database to identify and remove chimera sequence. Sequences passed this filtering step are deemed as clean data ready for analysis.

Section S15. 16S and ITS gene sequencing results

SI Table S4. 16S gene sequencing read count data for each sample day

Sample	Read Length [70]	# Reads	# Bases [70]	Q20 (%)	Q30 (%)	GC (%)	N (ppm)
S1	248.17	141,318	35,070,865	95.3	88.27	54.01	78.41
S2	233.31	5,360	1,250,555	94.53	87.46	49.2	2,804.35
S3	248.74	133,502	33,206,784	95.59	88.8	56.54	116.27
S4	236.86	8,854	2,097,158	94.95	88.1	51.85	2,592.56
S5	240.26	13,924	3,345,430	95.32	88.46	54.33	1,466.18
S6	247.09	61,778	15,264,916	95.48	88.54	56.38	159.65
S7	248.63	152,180	37,836,394	95.46	88.52	57.33	58.7
S8	247.02	86,540	21,377,094	95.64	88.91	56.1	134.3
S9	247.95	62,352	15,460,314	95.13	87.99	55.79	200

SI Table S5. 16S gene sequencing data optimization

Sample	# PE Reads	# Reads with no chimera	Average Read Length [70]	GC (%)	Effective (%)
S1	70,659	61,636	423.43	54.71	87.23
S2	2,680	2,251	370.22	50.12	83.99
S3	66,751	61,837	436.4	56.67	92.64
S4	4,427	3,822	409.44	53.01	86.33
S5	6,962	6,237	420.52	55.04	89.59
S6	30,889	28,350	432.76	56.74	91.78
S7	76,090	70,032	434.12	57.42	92.04
S8	43,270	38,976	422.79	56.47	90.08
S9	31,176	28,486	426.25	55.92	91.37

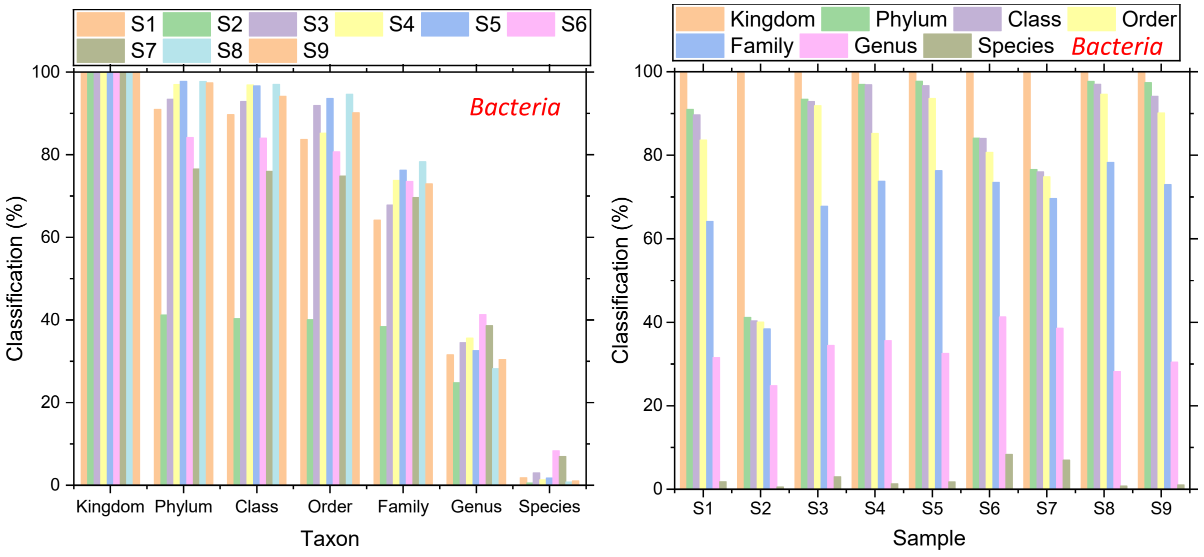

SI Figure S15. Success of taxonomic identification efforts of bacterial OTU reads. 60% of reads in S2 could not be classified past the phylum level. The bar graph on the left shows results grouped by taxa and the graph on the right shows results grouped by sampling date.

SI Table S6. ITS gene sequencing read count data for each sample

Sample	Read Length [70]	# Reads	# Bases [70]	Q20 (%)	Q30 (%)	GC (%)	N (ppm)
S1	248.83	192,860	47,989,241	96	89.34	51.24	62.47
S2	204.76	283,480	58,046,161	94.96	88.43	52.37	9,921.84
S3	245.98	230,390	56,671,786	95.64	88.87	53.47	604.46
S4	215.33	338,624	72,915,964	95.28	88.68	53.01	7,482.19
S5	193.3	282,330	54,573,834	94.9	88.52	50.99	13,679.72
S6	246.27	229,490	56,515,409	95.65	88.84	53.25	523.73
S7	244.73	261,980	64,115,168	95.72	89.1	54.19	847.21
S8	245.1	325,558	79,793,330	95.76	89.16	53.25	798.12
S9	243.91	235,048	57,330,107	95.66	88.98	53.38	1019.95

SI Table S7. ITS gene sequencing data quality optimization

Sample	# PE Reads	# Reads with no chimera	Average Read Length [70]	GC (%)	Effective (%)
S1	96,430	92,331	331.05	50.58	95.75
S2	141,740	108,290	310.23	51.82	76.4
S3	115,195	105,022	306.37	52.15	91.17
S4	169,312	131,815	310.03	52.13	77.85
S5	141,165	97,741	316.72	50.5	69.24
S6	114,745	103,077	314.28	51.84	89.83
S7	130,990	113,828	306.1	52.78	86.9
S8	162,779	143,655	306.58	51.99	88.25
S9	117,524	101,883	305.14	52.11	86.69

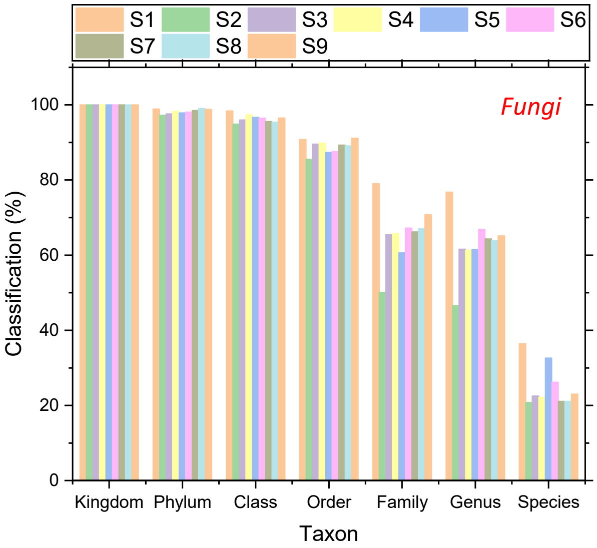

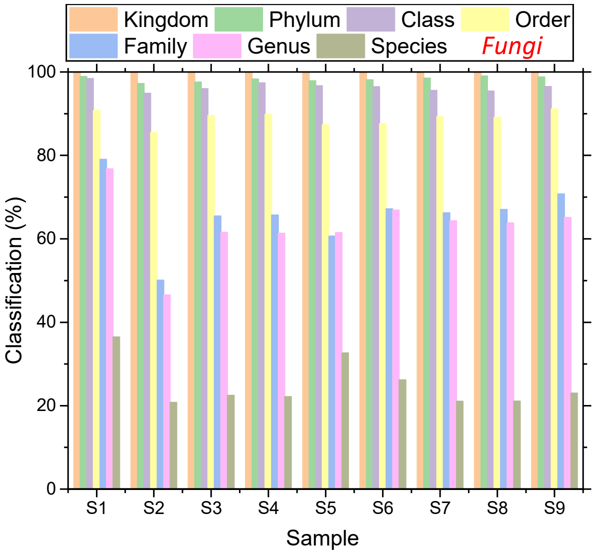

SI Figure S16. Success of taxonomic identification efforts of fungal OTU reads. 20% of reads on every sample were able to be classified to the species level. The bar graph on the left shows results grouped by taxa and the graph on the right shows results grouped by sampling date

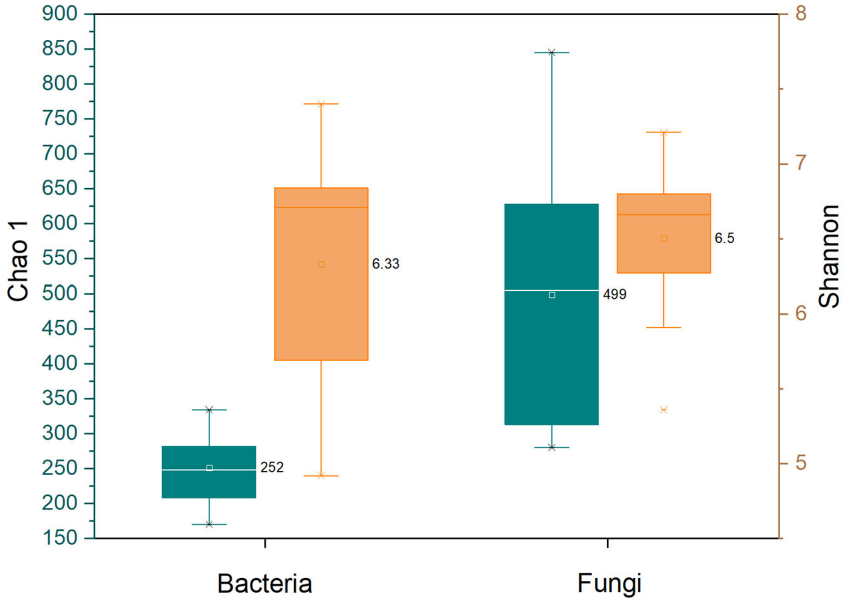

SI Figure S17. Total species (OTUs) estimated by the Chao1 Index (depicted using the green colored box on the left y-axis and the Shannon Index (orange colored box on the right y-axis) for microbial diversity for bacterial and fungal communities. The box encompasses the 25% and 75% percentiles, and the whiskers span 1.5 times the interquartile range. Inside each box, the horizontal line is the median value. The hollow square symbol (□) is the average value, which is also shown numerically.

Chao1 and Shannon indices are the current state of the art for summarizing the richness and biodiversity of ecological samples [71-73]. The Chao1 algorithm is an estimator of species richness that

has been applied in this case to estimate the true number of OTUs in each sample. It differs from other species richness indices by considering abundance data in place of presence/absence, or occurrence, data. It is dependent on the total number of species/OTUs observed and the ratio of singletons to doubletons [74].

$$S_{Chao1} = S_{obs} + \frac{{F_1}^2}{2{F_2}^2}$$

where

S_{obs} is the number of observed OTUs

F_1 is the number of singletons (OTUs represented by one individual)

F_2 is the number of doubletons (OTUs represented by two individuals)

The Shannon Index is a popular measure of heterogeneity, taking into account both richness and evenness of species. Our reported values for Shannon's index are reasonable in comparison with values reported in similar studies [53, 54, 61, 75]. As with any single metric, neither Chao nor Shannon faithfully capture all the nuances of the dataset, and there is vigorous discussion within the bioinformatics community, which is why we report both indices.

$$H' = -\sum p_i \ln p_i$$

where,

p_i is the proportion of individuals found in the ith OTU

On average, the fungal community registered more unique OTUs identified than the bacterial community and was more diverse, meaning OTUs were also present at more even frequencies. The number of bacterial OTUs from sample to sample were relatively stable compared to fungal OTUs. However, diversity, or heterogeneity, fluctuated more in the bacterial community, suggesting that a few bacterial OTUs dominated in some samples.

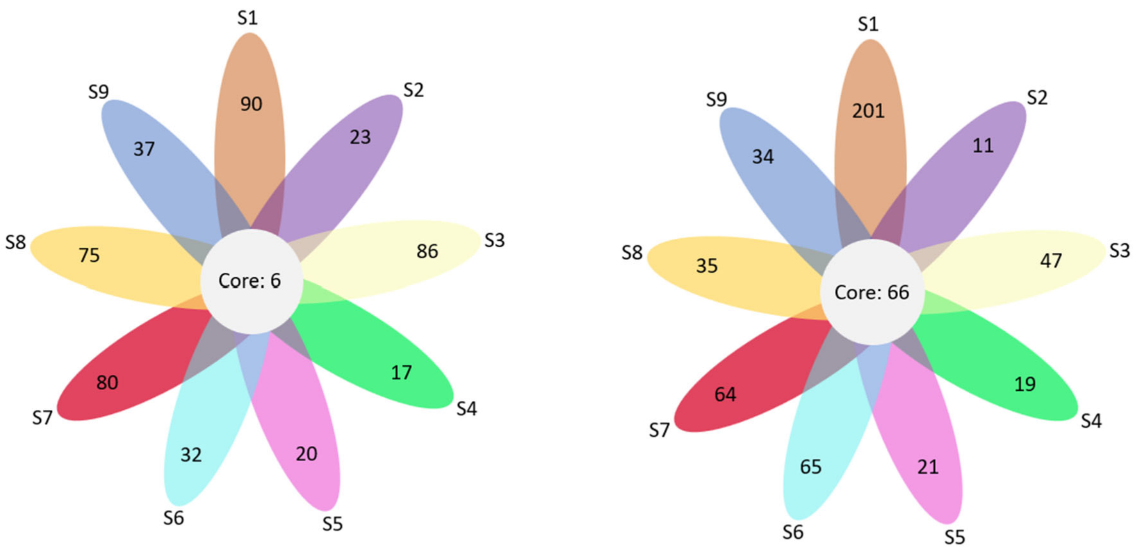

SI Figure S18. Unique and background OTUs for bacteria (left) and fungi (right). Each colored petal represents the number of OTUs that were exclusively detected in that sample. The center circle represents the number of OTUs whose abundance was non-zero for every sample. Fungi showed a larger set of core OTUs than bacteria. S1 had the largest set of exclusive OTUs with respect to both bacteria and fungi.

Samples S2, S4, and S5, which constituted three of four sample days with the highest Saharan dust presence (average 41 $\mu g/m^3$), showed substantially fewer reads (2,005, 3,412, and 5,639 counts, respectively). In contrast, S3, which was most influenced by Saharan dust registered over 56,000 successful reads. S7 and S1 showed the most reads over the sampling period (65,154 and 57,304 counts, respectively).

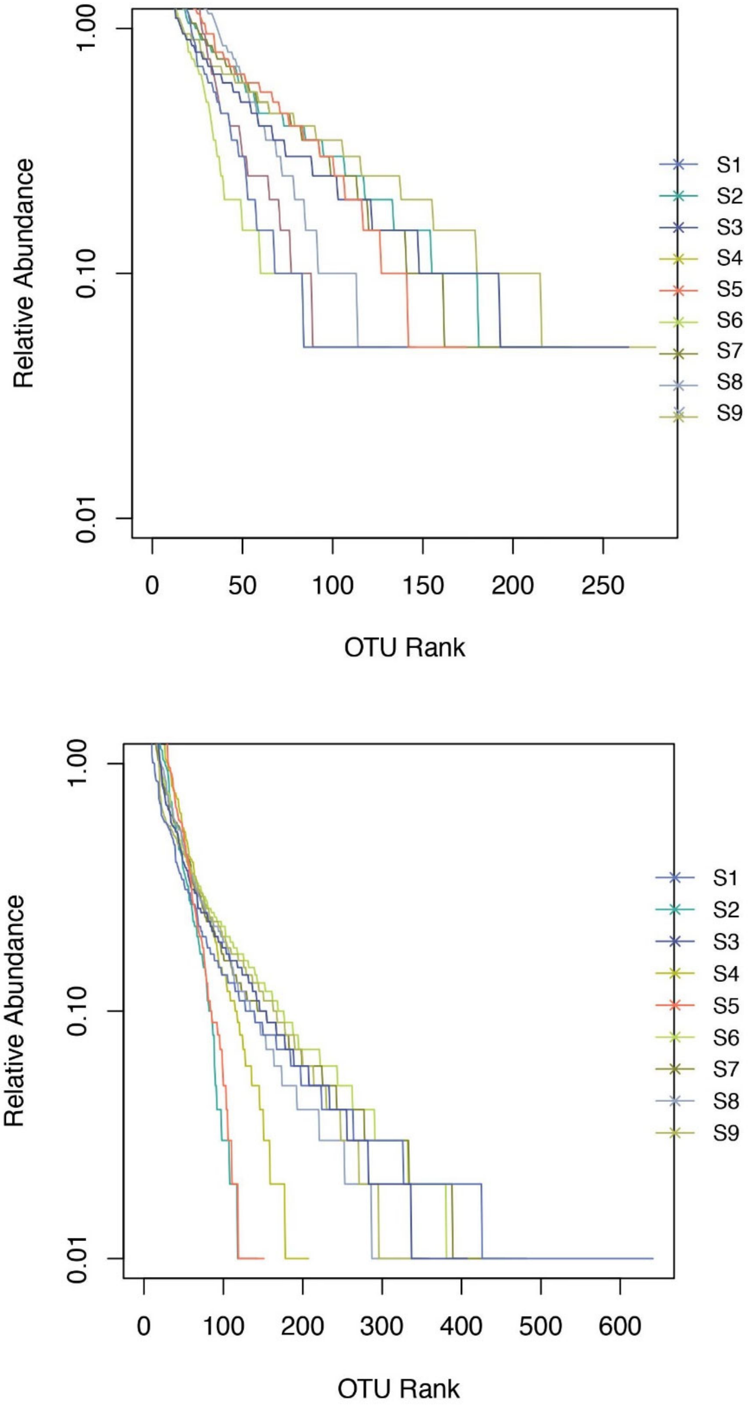

SI Figure S19. Rank-Abundance of bacteria (top) and fungi (bottom). Each curve in the figures above corresponds to an individual sample. The x axis is the relative abundance of the OTU in descending order. The y axis is the relative abundance of the OTU. '100' on the X axis indicates the OTU in the sample is ranked as the 100th abundant in descending order, and the corresponding value on the y axis is the percentage of the sequence count in the OTU (the number of sequences of the OTU divided by the total number of sequences). Rank-abundance curve reflects both species abundance and species uniformity. The abundance of species is reflected by the length of the curve on the x axis. The more extended on the x axis, the more abundant the species is. Species uniformity is reflected by the shape of the curve. The smoother the curve, the higher the species uniformity.

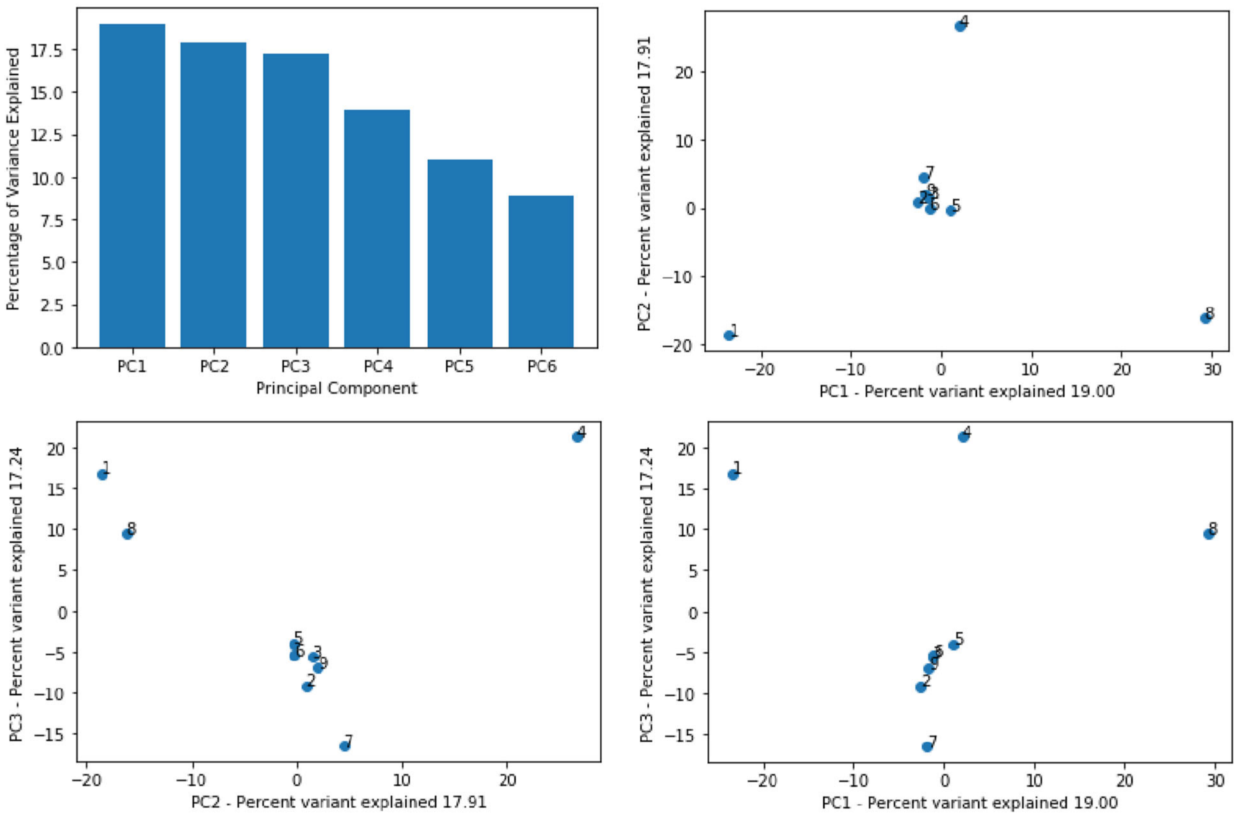

SI Figure S20. Principal component analysis performed on bacterial OTU relative abundance data. Scree plot (Top Left) showing the explanatory weight of the first 6 principal components. Biplots between PC1 and PC2 (Top Right), PC2 and PC3 (Bottom Left), and PC1 and PC3 (Bottom Right). S1, S4, and S8 were consistently dissimilar from the other samples.

Principal Component Analysis (PCA) is an ordination method that reduces complex data into terms of summary variables, or eigenvalues. As with any statistical analyses, PCA works best under certain assumed conditions, including Gaussian distributions and linearity among variables.[76] These conditions, as well as the type of distance matrix used to perform the analysis, differ from those of Principal Coordinate Analysis (PCoA) which is shown in SI Section S18 (SI Figures S26a and b and S27a and b). Note that both ordination methods have been used in previous studies on long-range transported bioaerosols.[53, 77, 78]

The first three principal components for bacteria explained roughly 54% of the total variance in the data. S1, S4, and S8 which correspond to both pre-, mid- and post-peak conditions were consistently plotted further away from the main cluster. The first three principal components for fungi also explained roughly

54% of the total variance in the data. With respect to PC1, S1, a low-dust sample corresponding with pre-peak conditions, was dissimilar from the rest of the samples. S6 was also consistently plotted further from the main cluster in all combinations of the first three principal components. S7, a low-dust sample corresponding to post-peak conditions was dissimilar from the rest of the samples with respect to PC2.

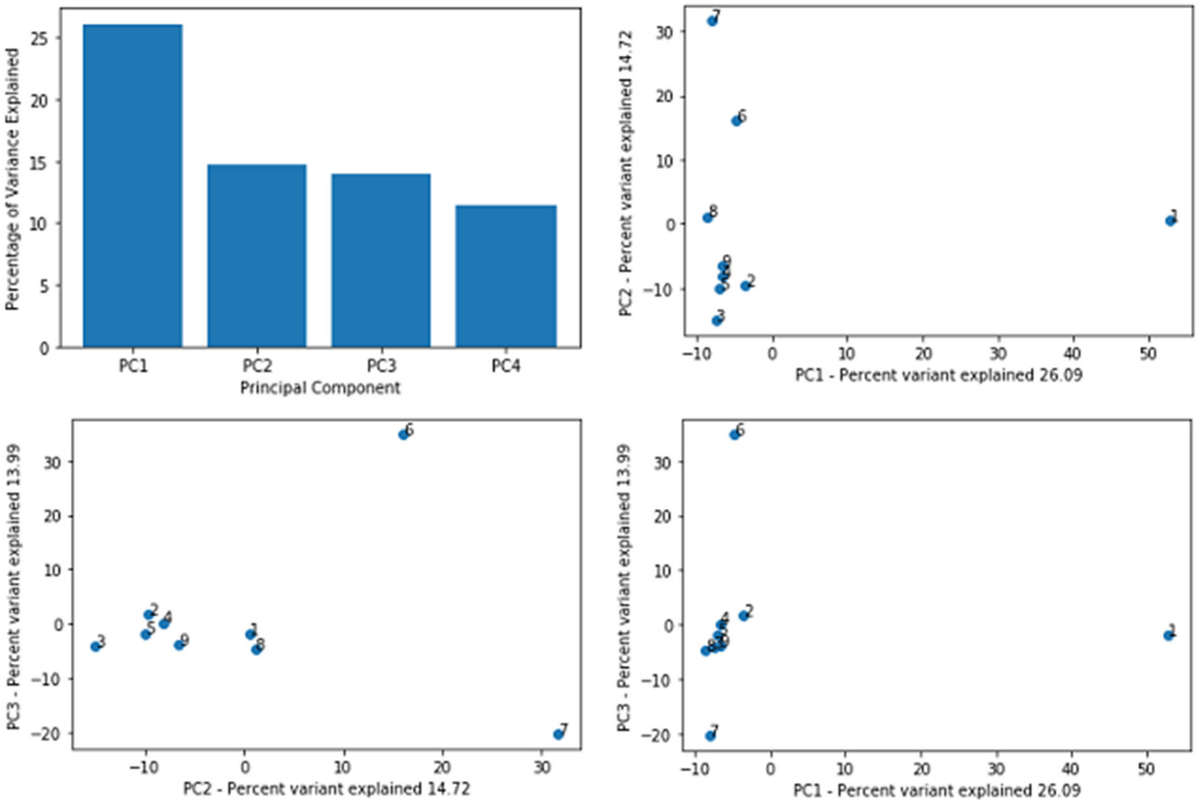

SI Figure S21. Principal component analysis performed on fungal OTU relative abundance data. Scree plot (Top Left) showing the explanatory weight of the first 4 principal components. Biplots between PC1 and PC2 (Top Right), PC2 and PC3 (Bottom Left), and PC1 and PC3 (Bottom Right). S6 was consistently dissimilar from the other samples. With respect to PC1, S1 was also highly dissimilar from the main cluster. With respect to PC2, S7 was also highly dissimilar from the main cluster.

PCA analysis revealed complex signals for both bacteria and fungi, which was our motivation for employing linear regression to examine daily fluctuations of individual OTU abundance, measuring phylogenetic signal to assess patterns of evolutionary conserved responses, and using GDM to present a model of turnover in community composition using both source apportionment and individual elemental concentrations.

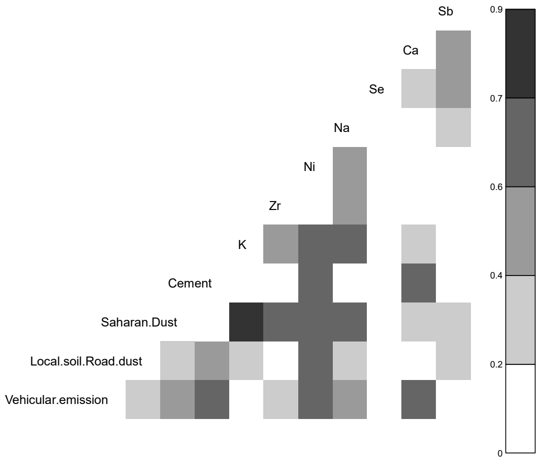

SI Figure S22. Collinearity among explanatory variables as determined using linear regressions. Darker colors signify stronger correlation between two explanatory variables. True white signifies a correlation coefficient between 0 and 0.2, and true black signifies correlation coefficients between 0.7 and 0.9. Grey shades represent correlation coefficients between o.2 and 0.7 as indicated in the vertical bar on the far right of the figure.

In comparing correlations between elemental and source concentrations, cement is positively associated ($r > 0.5$) with concentrations of Ni and Ca, vehicular emissions with Ni, Na, and Ca, Saharan dust with K, Zr, Ni, and Na, and local road dust with Na (**SI Figure S21**), indicating collinearity among these individual elements and composite aerosol sources.

SI Table S8. Complete list of significant relationships (p-value < 0.05) between bacteria and fungi source apportionments

Fungi			Bacteria		
OTU	p-value	r	OTU	p-value	r
Cement					
OTU162	0.024	0.735	OTU31	0.021	0.747
OTU212	0.005	0.838	OTU40	0.013	0.781
OTU215	0.012	0.788	OTU58	0.031	0.714
OTU421	0.009	0.807	OTU104	0.034	0.705
OTU337	0.008	0.809	OTU261	0.036	0.698
OTU424	0.044	0.680	OTU108	0.039	0.692
OTU453	0.003	0.862	OTU791	0.008	0.813
OTU581	0.010	0.799	OTU260	0.002	0.882
OTU959	0.007	0.817	OTU121	0.045	0.678
OTU416	0.010	0.800	OTU287	0.005	0.837
OTU507	0.011	0.793	OTU75	0.002	0.872
OTU509	0.025	0.732	OTU726	0.019	0.752
OTU376	0.024	0.735	OTU796	0.027	0.726

OTU508	0.004	0.843	OTU268	0.048	0.671
OTU503	0.002	0.877	OTU512	0.026	0.728
OTU514	0.004	0.848	OTU490	0.047	0.674
OTU562	0.039	0.692	OTU743	0.006	0.830
OTU603	0.006	0.826	OTU816	0.011	0.789
OTU381	0.048	0.671	OTU700	0.009	0.801
OTU721	0.019	0.755			
OTU730	0.006	0.829			
OTU377	0.003	0.857			
OTU564	0.012	0.785			
OTU515	0.002	0.874			
OTU580	0.011	0.794			
OTU738	0.010	0.798			
OTU1110	0.016	0.767			
OTU511	0.004	0.841			
OTU1230	0.008	0.813			
OTU628	0.024	0.736			
OTU876	0.005	0.839			
OTU1196	0.008	0.814			
OTU505	0.012	0.787			
OTU1270	0.005	0.833			
OTU717	0.034	0.703			
OTU646	0.023	0.737			
OTU955	0.016	0.766			
OTU993	0.005	0.834			
OTU1263	0.010	0.800			
OTU1215	0.002	0.884			
OTU904	0.012	0.786			
OTU743	0.008	0.813			
OTU1036	0.013	0.783			
OTU1284	0.002	0.875			
OTU596	0.047	0.674			
OTU1275	0.015	0.769			
OTU952	0.017	0.760			
**Local Soil & Road Dust**					
OTU22	0.010	-0.795	OTU261	0.038	0.694
OTU239	0.033	-0.708	OTU99	0.036	-0.698
OTU526	0.037	-0.697	OTU309	0.048	-0.671
OTU521	0.033	-0.707			
OTU338	0.038	-0.693			
OTU1038	0.025	0.732			
OTU1063	0.034	-0.706			
OTU1055	0.042	-0.685			

**Saharan Dust**					
OTU35	0.036	0.699	OTU102	0.007	0.817
OTU136	0.031	0.714	OTU9	0.018	0.759
OTU246	0.039	-0.692	OTU494	0.011	0.791
OTU260	0.021	0.746	OTU450	0.018	-0.760
OTU443	0.024	0.736	OTU29	0.022	0.741
OTU458	0.044	0.681			
OTU241	0.042	-0.685			
OTU654	0.026	0.727			
OTU335	0.050	-0.666			
OTU692	0.044	-0.680			
OTU657	0.012	0.785			
OTU1067	0.032	0.709			
**Vehicular Emissions**					
OTU264	0.011	-0.792	OTU86	0.044	0.679
OTU268	0.044	-0.680	OTU169	0.028	0.721
OTU399	0.024	0.736	OTU104	0.027	0.726
OTU337	0.025	0.730	OTU791	0.046	0.675
OTU424	0.043	0.681	OTU9	0.032	-0.709
OTU453	0.046	0.676	OTU43	0.046	-0.675
OTU581	0.016	0.767	OTU75	0.044	0.680
OTU959	0.038	0.694	OTU10	0.040	-0.688
OTU507	0.032	0.712	OTU450	0.039	0.692
OTU509	0.032	0.709	OTU280	0.036	0.698
OTU597	0.027	0.727	OTU435	0.044	0.679
OTU508	0.043	0.681	OTU796	0.020	0.751
OTU514	0.035	0.702	OTU13	0.036	-0.698
OTU550	0.019	0.752	OTU12	0.035	-0.702
OTU771	0.033	0.708	OTU82	0.046	-0.675
OTU562	0.016	0.768	OTU189	0.007	-0.816
OTU603	0.034	0.706			
OTU381	0.043	0.682			
OTU721	0.040	0.690			
OTU564	0.024	0.734			
OTU515	0.043	0.682			
OTU580	0.020	0.749			
OTU738	0.025	0.733			
OTU511	0.043	0.681			
OTU1230	0.031	0.713			
OTU876	0.042	0.685			
OTU718	0.017	0.764			
OTU505	0.039	0.693			
OTU1270	0.037	0.696			

OTU717	0.041	0.687	
OTU955	0.017	0.764	
OTU993	0.038	0.694	
OTU589	0.045	0.677	
OTU904	0.006	0.827	
OTU743	0.028	0.721	
OTU1036	0.017	0.762	
OTU596	0.016	0.766	
OTU1285	0.049	0.669	
OTU1275	0.020	0.749	
OTU952	0.019	0.752	
OTU1282	0.018	0.758	

SI Table S9. Complete list of significant relationships (p-value < 0.05) between bacteria and fungi to elemental data.

Fungi							
**OTU**	**Element**	**p-value**	**r**	**OTU**	**Element**	**p-value**	**r**
OTU35	Al	0.009	0.806	OTU1272	Mo	0.000	0.946
OTU136	Al	0.011	0.791	OTU871	Mo	0.004	0.843
OTU3	Al	0.027	0.725	OTU880	Mo	0.000	0.983
OTU443	Al	0.005	0.840	OTU609	Mo	0.046	0.675
OTU458	Al	0.038	0.694	OTU961	Mo	0.005	0.833
OTU5	Al	0.017	0.761	OTU589	Mo	0.000	0.947
OTU241	Al	0.035	-0.703	OTU596	Mo	0.039	0.691
OTU335	Al	0.043	-0.682	OTU1282	Mo	0.012	0.785
OTU692	Al	0.047	-0.673	OTU16	Na	0.048	-0.672
OTU657	Al	0.017	0.763	OTU174	Na	0.040	-0.690
OTU81	Al	0.028	-0.721	OTU202	Na	0.030	0.715
OTU177	Al	0.019	0.755	OTU420	Na	0.004	-0.849
OTU1067	Al	0.006	0.826	OTU768	Na	0.041	-0.686
OTU249	Al	0.013	0.779	OTU692	Na	0.015	-0.772
OTU675	Al	0.044	0.681	OTU857	Na	0.011	-0.791
OTU145	Al	0.047	0.673	OTU761	Na	0.034	-0.705
OTU1078	Al	0.033	0.708	OTU774	Na	0.034	-0.703
OTU2	As	0.016	-0.764	OTU431	Na	0.032	-0.710
OTU7	As	0.022	-0.741	OTU1306	Na	0.033	-0.708
OTU16	As	0.043	-0.682	OTU78	Ni	0.048	0.670
OTU68	As	0.016	-0.765	OTU54	Ni	0.020	0.752
OTU76	As	0.000	-0.944	OTU180	Ni	0.018	0.756
OTU204	As	0.007	-0.818	OTU323	Ni	0.045	0.678
OTU132	As	0.031	-0.713	OTU212	Ni	0.018	0.757
OTU272	As	0.025	-0.733	OTU342	Ni	0.024	0.735
OTU154	As	0.015	-0.769	OTU242	Ni	0.027	0.727
OTU359	As	0.043	0.682	OTU421	Ni	0.042	0.684
OTU241	As	0.015	-0.770	OTU399	Ni	0.001	0.902
OTU273	As	0.033	-0.707	OTU380	Ni	0.032	0.710
OTU335	As	0.032	-0.710	OTU150	Ni	0.033	0.707
OTU314	As	0.034	-0.704	OTU337	Ni	0.004	0.844
OTU494	As	0.030	-0.715	OTU424	Ni	0.000	0.922
OTU474	As	0.048	-0.672	OTU453	Ni	0.019	0.755
OTU92	As	0.043	-0.683	OTU581	Ni	0.009	0.805
OTU488	As	0.034	-0.704	OTU959	Ni	0.019	0.755
OTU4	Ba	0.011	-0.790	OTU570	Ni	0.017	0.762
OTU7	Ba	0.022	-0.744	OTU507	Ni	0.006	0.823
OTU204	Ba	0.030	-0.717	OTU509	Ni	0.048	0.671
OTU153	Ba	0.021	0.747	OTU914	Ni	0.003	0.866

OTU285	Ba	0.042	0.685	OTU376	Ni	0.024	0.737
OTU283	Ba	0.033	0.708	OTU597	Ni	0.004	0.849
OTU333	Ba	0.028	0.723	OTU508	Ni	0.015	0.773
OTU400	Ba	0.017	0.764	OTU503	Ni	0.008	0.814
OTU341	Ba	0.034	0.703	OTU514	Ni	0.007	0.821
OTU561	Ba	0.040	0.688	OTU550	Ni	0.002	0.875
OTU413	Ba	0.045	0.678	OTU562	Ni	0.001	0.894
OTU345	Ba	0.024	0.734	OTU603	Ni	0.008	0.810
OTU334	Ba	0.029	0.718	OTU381	Ni	0.038	0.695
OTU435	Ba	0.044	-0.681	OTU721	Ni	0.025	0.732
OTU595	Ba	0.043	0.682	OTU730	Ni	0.026	0.728
OTU434	Ba	0.044	0.679	OTU377	Ni	0.004	0.849
OTU336	Ba	0.046	0.675	OTU741	Ni	0.039	0.693
OTU551	Ba	0.038	0.694	OTU515	Ni	0.006	0.826
OTU875	Ba	0.019	0.754	OTU580	Ni	0.002	0.874
OTU613	Ba	0.015	0.769	OTU738	Ni	0.004	0.843
OTU863	Ba	0.036	0.699	OTU511	Ni	0.013	0.780
OTU961	Ba	0.019	0.755	OTU1230	Ni	0.010	0.801
OTU12	Ca	0.036	0.700	OTU876	Ni	0.012	0.785
OTU15	Ca	0.007	0.815	OTU1196	Ni	0.033	0.708
OTU37	Ca	0.009	0.807	OTU718	Ni	0.001	0.887
OTU36	Ca	0.001	0.894	OTU505	Ni	0.019	0.754
OTU26	Ca	0.028	0.721	OTU1272	Ni	0.045	0.678
OTU33	Ca	0.005	0.832	OTU1270	Ni	0.010	0.798
OTU38	Ca	0.015	0.773	OTU717	Ni	0.025	0.731
OTU45	Ca	0.025	0.733	OTU955	Ni	0.004	0.849
OTU42	Ca	0.027	0.727	OTU880	Ni	0.040	0.688
OTU63	Ca	0.016	0.768	OTU993	Ni	0.010	0.796
OTU61	Ca	0.049	0.669	OTU1263	Ni	0.026	0.728
OTU55	Ca	0.008	0.811	OTU589	Ni	0.013	0.780
OTU670	Ca	0.021	0.745	OTU904	Ni	0.027	0.724
OTU53	Ca	0.021	0.746	OTU743	Ni	0.006	0.828
OTU66	Ca	0.025	0.730	OTU1036	Ni	0.014	0.778
OTU46	Ca	0.020	0.749	OTU596	Ni	0.001	0.887
OTU50	Ca	0.020	0.748	OTU1275	Ni	0.003	0.864
OTU73	Ca	0.039	0.691	OTU952	Ni	0.002	0.868
OTU64	Ca	0.006	0.826	OTU1282	Ni	0.002	0.881
OTU65	Ca	0.025	0.733	OTU18	Pb	0.041	-0.687
OTU95	Ca	0.010	0.797	OTU15	Pb	0.035	0.701
OTU59	Ca	0.003	0.859	OTU37	Pb	0.040	0.689
OTU136	Ca	0.030	0.716	OTU35	Pb	0.048	0.670
OTU1	Ca	0.004	0.850	OTU36	Pb	0.036	0.700
OTU149	Ca	0.001	0.887	OTU33	Pb	0.034	0.704

OTU103	Ca	0.023	0.740	OTU38	Pb	0.035	0.702
OTU87	Ca	0.033	0.706	OTU48	Pb	0.032	-0.709
OTU125	Ca	0.013	0.782	OTU45	Pb	0.044	0.680
OTU116	Ca	0.008	0.809	OTU42	Pb	0.041	0.687
OTU118	Ca	0.023	0.738	OTU55	Pb	0.017	0.761
OTU114	Ca	0.032	0.710	OTU670	Pb	0.038	0.695
OTU237	Ca	0.038	0.695	OTU53	Pb	0.030	0.717
OTU298	Ca	0.012	0.784	OTU46	Pb	0.024	0.735
OTU162	Ca	0.036	0.700	OTU50	Pb	0.035	0.701
OTU168	Ca	0.022	0.742	OTU65	Pb	0.043	0.682
OTU144	Ca	0.018	0.760	OTU95	Pb	0.013	0.782
OTU206	Ca	0.024	0.735	OTU59	Pb	0.002	0.867
OTU169	Ca	0.021	0.745	OTU1	Pb	0.029	0.719
OTU537	Ca	0.047	0.674	OTU149	Pb	0.005	0.837
OTU198	Ca	0.033	0.707	OTU103	Pb	0.047	0.673
OTU248	Ca	0.008	0.811	OTU146	Pb	0.042	0.684
OTU268	Ca	0.042	-0.684	OTU87	Pb	0.038	0.693
OTU232	Ca	0.022	0.742	OTU125	Pb	0.037	0.696
OTU355	Ca	0.013	0.783	OTU116	Pb	0.037	0.697
OTU471	Ca	0.023	0.737	OTU114	Pb	0.039	0.692
OTU423	Ca	0.007	0.819	OTU237	Pb	0.029	0.720
OTU364	Ca	0.025	0.733	OTU298	Pb	0.000	0.955
OTU10	Ca	0.028	0.724	OTU162	Pb	0.034	0.706
OTU669	Ca	0.008	0.812	OTU168	Pb	0.040	0.690
OTU676	Ca	0.004	0.842	OTU206	Pb	0.041	0.686
OTU472	Ca	0.022	0.744	OTU225	Pb	0.010	0.801
OTU671	Ca	0.048	0.671	OTU169	Pb	0.046	0.674
OTU564	Ca	0.040	0.688	OTU537	Pb	0.037	0.696
OTU367	Ca	0.021	0.748	OTU248	Pb	0.001	0.889
OTU52	Ca	0.028	-0.722	OTU232	Pb	0.039	0.692
OTU674	Ca	0.009	0.803	OTU355	Pb	0.017	0.760
OTU404	Ca	0.045	0.678	OTU471	Pb	0.024	0.736
OTU93	Ca	0.035	-0.702	OTU423	Pb	0.019	0.753
OTU1142	Ca	0.024	0.734	OTU10	Pb	0.047	0.673
OTU105	Ca	0.050	-0.667	OTU669	Pb	0.014	0.776
OTU97	Ca	0.006	0.827	OTU676	Pb	0.020	0.749
OTU177	Ca	0.015	0.769	OTU671	Pb	0.033	0.706
OTU104	Ca	0.018	0.758	OTU564	Pb	0.037	0.696
OTU233	Ca	0.010	0.800	OTU674	Pb	0.013	0.779
OTU675	Ca	0.009	0.807	OTU404	Pb	0.047	0.673
OTU1078	Ca	0.009	0.806	OTU177	Pb	0.013	0.782
OTU1145	Ca	0.032	0.710	OTU233	Pb	0.023	0.738
OTU56	Cd	0.008	0.813	OTU675	Pb	0.017	0.762

OTU49	Cd	0.009	0.804	OTU1078	Pb	0.014	0.776
OTU51	Cd	0.037	0.698	OTU9	Sb	0.012	-0.785
OTU71	Cd	0.031	0.713	OTU17	Sb	0.014	-0.777
OTU70	Cd	0.033	0.707	OTU20	Sb	0.047	-0.673
OTU102	Cd	0.013	0.783	OTU62	Sb	0.030	-0.717
OTU83	Cd	0.006	0.831	OTU67	Sb	0.037	0.697
OTU173	Cd	0.030	0.717	OTU85	Sb	0.025	0.731
OTU91	Cd	0.017	0.762	OTU228	Sb	0.044	-0.679
OTU96	Cd	0.039	0.691	OTU99	Sb	0.017	0.763
OTU163	Cd	0.014	0.776	OTU146	Sb	0.019	-0.753
OTU100	Cd	0.003	0.863	OTU119	Sb	0.012	0.785
OTU79	Cd	0.021	0.746	OTU147	Sb	0.030	0.716
OTU181	Cd	0.031	0.713	OTU127	Sb	0.012	0.784
OTU222	Cd	0.022	0.742	OTU199	Sb	0.024	0.734
OTU217	Cd	0.015	0.769	OTU159	Sb	0.025	0.733
OTU124	Cd	0.021	0.745	OTU179	Sb	0.044	0.679
OTU141	Cd	0.036	0.698	OTU151	Sb	0.007	0.815
OTU302	Cd	0.024	0.734	OTU250	Sb	0.020	0.751
OTU164	Cd	0.028	0.724	OTU290	Sb	0.021	0.747
OTU183	Cd	0.022	0.741	OTU348	Sb	0.024	0.734
OTU160	Cd	0.034	0.705	OTU618	Sb	0.017	0.764
OTU185	Cd	0.023	0.738	OTU455	Sb	0.044	-0.680
OTU184	Cd	0.021	0.746	OTU430	Sb	0.005	0.832
OTU143	Cd	0.017	0.763	OTU111	Se	0.045	0.678
OTU263	Cd	0.019	0.753	OTU196	Se	0.021	0.745
OTU208	Cd	0.024	0.736	OTU218	Se	0.042	0.684
OTU207	Cd	0.023	0.738	OTU197	Se	0.025	0.733
OTU287	Cd	0.038	0.694	OTU179	Se	0.014	-0.776
OTU261	Cd	0.011	0.793	OTU332	Se	0.028	0.721
OTU265	Cd	0.016	0.765	OTU403	Se	0.035	0.701
OTU303	Cd	0.028	0.723	OTU559	Se	0.025	0.730
OTU466	Cd	0.023	0.739	OTU601	Se	0.030	0.715
OTU266	Cd	0.012	0.786	OTU481	Se	0.031	0.714
OTU661	Cd	0.030	0.717	OTU852	Se	0.026	0.727
OTU463	Cd	0.024	0.734	OTU422	Se	0.027	0.725
OTU1127	Cd	0.039	0.691	OTU861	Se	0.033	0.708
OTU465	Cd	0.024	0.735	OTU280	Se	0.038	0.694
OTU165	Cd	0.028	0.721	OTU433	Se	0.025	0.732
OTU35	Ce	0.023	0.737	OTU497	Se	0.040	0.690
OTU136	Ce	0.013	0.779	OTU563	Se	0.022	0.744
OTU76	Ce	0.046	-0.674	OTU815	Se	0.027	0.726
OTU101	Ce	0.047	0.672	OTU411	Se	0.017	0.763
OTU246	Ce	0.039	-0.692	OTU574	Se	0.035	0.703

OTU260	Ce	0.019	0.754	OTU872	Se	0.019	0.754
OTU443	Ce	0.040	0.689	OTU711	Se	0.019	0.755
OTU241	Ce	0.031	-0.713	OTU1020	Se	0.024	0.736
OTU654	Ce	0.019	0.753	OTU988	Se	0.019	0.752
OTU335	Ce	0.034	-0.705	OTU882	Se	0.031	0.712
OTU692	Ce	0.048	-0.670	OTU15	Si	0.047	0.673
OTU657	Ce	0.031	0.713	OTU35	Si	0.016	0.766
OTU610	Ce	0.046	-0.675	OTU43	Si	0.035	0.701
OTU1067	Ce	0.048	0.670	OTU95	Si	0.035	0.702
OTU27	Co	0.039	0.691	OTU59	Si	0.027	0.727
OTU51	Co	0.038	0.694	OTU136	Si	0.004	0.843
OTU71	Co	0.044	0.680	OTU149	Si	0.039	0.691
OTU70	Co	0.016	0.767	OTU174	Si	0.050	-0.667
OTU136	Co	0.025	0.731	OTU298	Si	0.008	0.809
OTU76	Co	0.045	-0.678	OTU443	Si	0.039	0.691
OTU101	Co	0.019	0.755	OTU423	Si	0.046	0.676
OTU1025	Co	0.047	0.673	OTU420	Si	0.026	-0.730
OTU141	Co	0.021	0.746	OTU768	Si	0.035	-0.703
OTU164	Co	0.030	0.716	OTU759	Si	0.011	-0.794
OTU183	Co	0.041	0.687	OTU530	Si	0.022	-0.744
OTU160	Co	0.050	0.667	OTU967	Si	0.038	-0.694
OTU185	Co	0.041	0.686	OTU760	Si	0.047	-0.672
OTU246	Co	0.040	-0.689	OTU761	Si	0.035	-0.701
OTU208	Co	0.041	0.686	OTU177	Si	0.022	0.742
OTU207	Co	0.032	0.711	OTU1067	Si	0.048	0.671
OTU260	Co	0.007	0.821	OTU249	Si	0.049	0.669
OTU241	Co	0.036	-0.699	OTU675	Si	0.049	0.668
OTU654	Co	0.007	0.819	OTU774	Si	0.028	-0.722
OTU335	Co	0.036	-0.700	OTU431	Si	0.026	-0.729
OTU466	Co	0.045	0.678	OTU1078	Si	0.038	0.694
OTU463	Co	0.049	0.669	OTU1306	Si	0.027	-0.727
OTU353	Co	0.047	0.673	OTU4	Sn	0.021	-0.748
OTU668	Co	0.027	0.727	OTU25	Sn	0.002	0.879
OTU610	Co	0.037	-0.696	OTU9	Sn	0.020	-0.749
OTU465	Co	0.049	0.668	OTU14	Sn	0.024	-0.735
OTU76	Cr	0.002	-0.872	OTU30	Sn	0.000	0.952
OTU204	Cr	0.029	-0.719	OTU28	Sn	0.025	0.732
OTU132	Cr	0.030	-0.717	OTU291	Sn	0.002	0.876
OTU272	Cr	0.024	-0.735	OTU85	Sn	0.012	0.787
OTU154	Cr	0.041	-0.687	OTU172	Sn	0.000	0.922
OTU191	Cr	0.029	-0.719	OTU197	Sn	0.030	0.715
OTU268	Cr	0.009	-0.806	OTU246	Sn	0.039	0.692
OTU529	Cr	0.033	-0.708	OTU285	Sn	0.000	0.954

OTU528	Cr	0.048	-0.671	OTU195	Sn	0.006	0.828
OTU435	Cr	0.030	-0.717	OTU283	Sn	0.000	0.969
OTU390	Cr	0.033	-0.707	OTU333	Sn	0.000	0.979
OTU773	Cr	0.030	-0.716	OTU1320	Sn	0.037	-0.698
OTU799	Cr	0.041	-0.688	OTU400	Sn	0.004	0.841
OTU772	Cr	0.033	-0.707	OTU193	Sn	0.000	0.972
OTU1067	Cr	0.043	0.682	OTU341	Sn	0.000	0.974
OTU145	Cr	0.034	0.704	OTU561	Sn	0.000	0.935
OTU18	Cu	0.018	-0.757	OTU342	Sn	0.031	0.713
OTU204	Cu	0.024	-0.736	OTU242	Sn	0.003	0.859
OTU248	Cu	0.007	0.819	OTU413	Sn	0.000	0.950
OTU268	Cu	0.008	-0.814	OTU345	Sn	0.000	0.977
OTU247	Cu	0.050	-0.667	OTU150	Sn	0.007	0.821
OTU564	Cu	0.023	0.739	OTU403	Sn	0.019	0.754
OTU628	Cu	0.042	0.683	OTU334	Sn	0.009	0.806
OTU674	Cu	0.050	0.667	OTU408	Sn	0.005	0.839
OTU904	Cu	0.020	0.748	OTU570	Sn	0.042	0.683
OTU1036	Cu	0.046	0.674	OTU552	Sn	0.001	0.896
OTU35	Fe	0.010	0.795	OTU595	Sn	0.000	0.931
OTU68	Fe	0.050	-0.667	OTU597	Sn	0.049	0.668
OTU136	Fe	0.013	0.783	OTU434	Sn	0.000	0.945
OTU76	Fe	0.029	-0.719	OTU616	Sn	0.000	0.948
OTU132	Fe	0.044	-0.679	OTU336	Sn	0.000	0.934
OTU801	Fe	0.045	-0.676	OTU551	Sn	0.000	0.957
OTU154	Fe	0.026	-0.729	OTU741	Sn	0.001	0.886
OTU246	Fe	0.043	-0.681	OTU875	Sn	0.000	0.955
OTU3	Fe	0.029	0.718	OTU613	Sn	0.009	0.806
OTU709	Fe	0.039	-0.693	OTU690	Sn	0.002	0.869
OTU443	Fe	0.016	0.768	OTU600	Sn	0.001	0.894
OTU5	Fe	0.024	0.734	OTU574	Sn	0.020	0.750
OTU241	Fe	0.021	-0.748	OTU910	Sn	0.024	0.734
OTU335	Fe	0.014	-0.774	OTU812	Sn	0.000	0.959
OTU494	Fe	0.047	-0.673	OTU863	Sn	0.001	0.888
OTU657	Fe	0.043	0.681	OTU1272	Sn	0.007	0.818
OTU81	Fe	0.049	-0.668	OTU871	Sn	0.001	0.887
OTU591	Fe	0.041	-0.688	OTU880	Sn	0.001	0.890
OTU573	Fe	0.042	-0.685	OTU609	Sn	0.017	0.763
OTU857	Fe	0.041	-0.687	OTU961	Sn	0.001	0.902
OTU610	Fe	0.030	-0.715	OTU589	Sn	0.010	0.798
OTU177	Fe	0.027	0.725	OTU15	Sr	0.012	0.788
OTU1067	Fe	0.018	0.758	OTU27	Sr	0.018	0.757
OTU249	Fe	0.020	0.752	OTU35	Sr	0.006	0.826
OTU675	Fe	0.048	0.671	OTU36	Sr	0.031	0.713

OTU1078	Fe	0.038	0.694	OTU43	Sr	0.046	0.676
OTU75	K	0.020	0.749	OTU73	Sr	0.038	0.694
OTU443	K	0.002	0.876	OTU95	Sr	0.017	0.762
OTU458	K	0.003	0.865	OTU59	Sr	0.019	0.755
OTU5	K	0.028	0.724	OTU136	Sr	0.001	0.905
OTU444	K	0.006	0.824	OTU149	Sr	0.027	0.726
OTU351	K	0.013	0.779	OTU116	Sr	0.030	0.717
OTU294	K	0.021	0.746	OTU3	Sr	0.043	0.683
OTU627	K	0.043	0.681	OTU443	Sr	0.023	0.739
OTU340	K	0.011	0.794	OTU423	Sr	0.029	0.719
OTU446	K	0.018	0.757	OTU676	Sr	0.039	0.691
OTU292	K	0.050	0.667	OTU106	Sr	0.047	-0.672
OTU357	K	0.005	0.831	OTU84	Sr	0.033	-0.707
OTU554	K	0.003	0.854	OTU105	Sr	0.025	-0.733
OTU629	K	0.009	0.805	OTU177	Sr	0.010	0.795
OTU457	K	0.035	0.703	OTU1067	Sr	0.027	0.727
OTU352	K	0.004	0.852	OTU233	Sr	0.038	0.693
OTU624	K	0.004	0.844	OTU249	Sr	0.031	0.712
OTU631	K	0.004	0.846	OTU675	Sr	0.016	0.766
OTU451	K	0.004	0.845	OTU1078	Sr	0.013	0.782
OTU346	K	0.009	0.804	OTU35	Ti	0.023	0.739
OTU417	K	0.018	0.758	OTU136	Ti	0.016	0.768
OTU452	K	0.005	0.836	OTU76	Ti	0.049	-0.669
OTU657	K	0.000	0.917	OTU801	Ti	0.050	-0.667
OTU81	K	0.042	-0.684	OTU154	Ti	0.039	-0.691
OTU459	K	0.003	0.854	OTU246	Ti	0.029	-0.720
OTU432	K	0.010	0.795	OTU709	Ti	0.050	-0.667
OTU406	K	0.006	0.831	OTU260	Ti	0.023	0.737
OTU448	K	0.021	0.744	OTU241	Ti	0.034	-0.704
OTU447	K	0.044	0.680	OTU654	Ti	0.024	0.735
OTU582	K	0.013	0.780	OTU335	Ti	0.025	-0.733
OTU652	K	0.003	0.856	OTU692	Ti	0.038	-0.695
OTU445	K	0.022	0.741	OTU657	Ti	0.048	0.670
OTU649	K	0.004	0.843	OTU857	Ti	0.045	-0.677
OTU644	K	0.008	0.809	OTU610	Ti	0.035	-0.701
OTU655	K	0.013	0.783	OTU1338	V	0.047	0.674
OTU639	K	0.004	0.853	OTU15	V	0.050	0.667
OTU991	K	0.004	0.846	OTU27	V	0.032	0.710
OTU626	K	0.004	0.843	OTU35	V	0.010	0.796
OTU1095	K	0.004	0.841	OTU136	V	0.006	0.829
OTU660	K	0.004	0.852	OTU801	V	0.041	-0.687
OTU634	K	0.012	0.784	OTU141	V	0.048	0.670
OTU1088	K	0.004	0.843	OTU246	V	0.027	-0.726

OTU656	K	0.027	0.727	OTU260	V	0.047	0.672
OTU1079	K	0.049	0.668	OTU443	V	0.042	0.684
OTU1098	K	0.005	0.836	OTU241	V	0.038	-0.695
OTU1067	K	0.003	0.860	OTU654	V	0.047	0.674
OTU249	K	0.034	0.705	OTU177	V	0.032	0.710
OTU1100	K	0.005	0.837	OTU1078	V	0.047	0.674
OTU35	La	0.016	0.764	OTU4	Zn	0.014	-0.777
OTU136	La	0.015	0.773	OTU25	Zn	0.000	0.924
OTU76	La	0.030	-0.716	OTU14	Zn	0.047	-0.673
OTU154	La	0.039	-0.691	OTU30	Zn	0.000	0.947
OTU709	La	0.049	-0.668	OTU28	Zn	0.015	0.771
OTU260	La	0.024	0.734	OTU291	Zn	0.006	0.826
OTU443	La	0.036	0.700	OTU111	Zn	0.024	0.734
OTU241	La	0.018	-0.757	OTU172	Zn	0.003	0.858
OTU654	La	0.026	0.728	OTU218	Zn	0.042	0.684
OTU335	La	0.023	-0.739	OTU197	Zn	0.013	0.779
OTU494	La	0.044	-0.679	OTU246	Zn	0.031	0.712
OTU692	La	0.041	-0.686	OTU285	Zn	0.000	0.987
OTU657	La	0.036	0.700	OTU195	Zn	0.012	0.786
OTU610	La	0.045	-0.678	OTU283	Zn	0.000	0.987
OTU1067	La	0.040	0.689	OTU333	Zn	0.000	0.985
OTU27	Mg	0.049	0.669	OTU400	Zn	0.003	0.855
OTU35	Mg	0.010	0.798	OTU193	Zn	0.000	0.962
OTU136	Mg	0.003	0.858	OTU341	Zn	0.000	0.980
OTU246	Mg	0.026	-0.727	OTU561	Zn	0.000	0.972
OTU443	Mg	0.030	0.716	OTU342	Zn	0.004	0.848
OTU241	Mg	0.050	-0.667	OTU242	Zn	0.001	0.900
OTU335	Mg	0.046	-0.675	OTU413	Zn	0.000	0.975
OTU857	Mg	0.037	-0.697	OTU345	Zn	0.000	0.979
OTU610	Mg	0.038	-0.695	OTU150	Zn	0.003	0.854
OTU177	Mg	0.033	0.708	OTU403	Zn	0.003	0.861
OTU1067	Mg	0.038	0.695	OTU601	Zn	0.047	0.674
OTU249	Mg	0.043	0.681	OTU334	Zn	0.012	0.784
OTU1078	Mg	0.048	0.672	OTU408	Zn	0.000	0.926
OTU15	Mn	0.039	0.692	OTU570	Zn	0.008	0.808
OTU35	Mn	0.008	0.808	OTU552	Zn	0.000	0.961
OTU95	Mn	0.021	0.747	OTU595	Zn	0.000	0.946
OTU59	Mn	0.023	0.737	OTU597	Zn	0.037	0.697
OTU136	Mn	0.016	0.768	OTU434	Zn	0.000	0.978
OTU76	Mn	0.008	-0.812	OTU852	Zn	0.024	0.734
OTU149	Mn	0.050	0.667	OTU616	Zn	0.004	0.842
OTU204	Mn	0.035	-0.701	OTU336	Zn	0.000	0.978
OTU132	Mn	0.034	-0.705	OTU551	Zn	0.000	0.973

OTU272	Mn	0.041	-0.687	OTU741	Zn	0.001	0.905
OTU154	Mn	0.029	-0.719	OTU875	Zn	0.000	0.970
OTU3	Mn	0.023	0.740	OTU613	Zn	0.007	0.820
OTU443	Mn	0.017	0.762	OTU433	Zn	0.011	0.795
OTU248	Mn	0.045	0.677	OTU690	Zn	0.000	0.943
OTU268	Mn	0.020	-0.750	OTU568	Zn	0.041	0.688
OTU5	Mn	0.024	0.735	OTU600	Zn	0.000	0.958
OTU241	Mn	0.021	-0.748	OTU574	Zn	0.003	0.861
OTU335	Mn	0.025	-0.732	OTU910	Zn	0.018	0.757
OTU423	Mn	0.049	0.668	OTU812	Zn	0.003	0.861
OTU81	Mn	0.045	-0.677	OTU863	Zn	0.000	0.917
OTU573	Mn	0.045	-0.678	OTU1272	Zn	0.003	0.854
OTU84	Mn	0.048	-0.670	OTU871	Zn	0.000	0.957
OTU177	Mn	0.013	0.780	OTU880	Zn	0.001	0.908
OTU1067	Mn	0.014	0.777	OTU609	Zn	0.022	0.742
OTU249	Mn	0.012	0.784	OTU961	Zn	0.000	0.928
OTU675	Mn	0.019	0.755	OTU589	Zn	0.006	0.823
OTU145	Mn	0.024	0.735	OTU49	Zr	0.008	0.813
OTU1078	Mn	0.015	0.773	OTU71	Zr	0.007	0.822
OTU4	Mo	0.027	-0.726	OTU70	Zr	0.000	0.931
OTU25	Mo	0.009	0.803	OTU88	Zr	0.008	0.810
OTU30	Mo	0.003	0.860	OTU102	Zr	0.003	0.865
OTU28	Mo	0.044	0.679	OTU76	Zr	0.025	-0.733
OTU291	Mo	0.038	0.695	OTU173	Zr	0.003	0.863
OTU172	Mo	0.023	0.739	OTU101	Zr	0.015	0.770
OTU246	Mo	0.039	0.692	OTU91	Zr	0.002	0.881
OTU285	Mo	0.001	0.903	OTU96	Zr	0.040	0.688
OTU283	Mo	0.001	0.906	OTU163	Zr	0.002	0.870
OTU333	Mo	0.000	0.921	OTU1025	Zr	0.004	0.852
OTU400	Mo	0.016	0.768	OTU100	Zr	0.010	0.800
OTU193	Mo	0.003	0.861	OTU79	Zr	0.003	0.862
OTU341	Mo	0.000	0.954	OTU128	Zr	0.038	0.693
OTU561	Mo	0.003	0.863	OTU124	Zr	0.005	0.840
OTU342	Mo	0.009	0.804	OTU141	Zr	0.003	0.862
OTU242	Mo	0.000	0.963	OTU302	Zr	0.001	0.891
OTU399	Mo	0.039	0.691	OTU164	Zr	0.001	0.909
OTU413	Mo	0.000	0.969	OTU183	Zr	0.001	0.900
OTU345	Mo	0.001	0.913	OTU160	Zr	0.028	0.721
OTU150	Mo	0.000	0.950	OTU185	Zr	0.001	0.903
OTU403	Mo	0.035	0.702	OTU184	Zr	0.007	0.818
OTU408	Mo	0.000	0.918	OTU264	Zr	0.027	0.726
OTU570	Mo	0.012	0.784	OTU263	Zr	0.001	0.886
OTU552	Mo	0.005	0.834	OTU208	Zr	0.001	0.903

OTU595	Mo	0.000	0.984	OTU207	Zr	0.001	0.906
OTU597	Mo	0.002	0.877	OTU287	Zr	0.001	0.905
OTU434	Mo	0.000	0.964	OTU260	Zr	0.004	0.842
OTU550	Mo	0.008	0.812	OTU261	Zr	0.017	0.762
OTU562	Mo	0.048	0.671	OTU265	Zr	0.002	0.871
OTU616	Mo	0.017	0.762	OTU359	Zr	0.020	0.750
OTU336	Mo	0.002	0.872	OTU303	Zr	0.006	0.830
OTU551	Mo	0.000	0.975	OTU267	Zr	0.004	0.844
OTU741	Mo	0.000	0.982	OTU654	Zr	0.006	0.830
OTU875	Mo	0.001	0.887	OTU466	Zr	0.001	0.905
OTU613	Mo	0.026	0.730	OTU266	Zr	0.005	0.836
OTU690	Mo	0.008	0.811	OTU469	Zr	0.022	0.742
OTU568	Mo	0.036	0.701	OTU463	Zr	0.001	0.907
OTU600	Mo	0.005	0.834	OTU353	Zr	0.029	0.719
OTU574	Mo	0.038	0.693	OTU668	Zr	0.008	0.810
OTU718	Mo	0.023	0.740	OTU1127	Zr	0.021	0.747
OTU812	Mo	0.013	0.782	OTU465	Zr	0.003	0.864
OTU863	Mo	0.000	0.970	OTU666	Zr	0.001	0.911
				OTU1113	Zr	0.001	0.909
**Bacteria**							
**OTU**	**Element**	**p-value**	**r**	**OTU**	**Element**	**p-value**	**r**
OTU267	Al	0.025	0.731	OTU43	Na	0.007	0.822
OTU105	Al	0.047	0.672	OTU10	Na	0.007	0.821
OTU110	Al	0.045	0.676	OTU450	Na	0.007	-0.819
OTU102	Al	0.014	0.774	OTU147	Na	0.016	-0.768
OTU494	Al	0.021	0.746	OTU4	Na	0.032	0.709
OTU208	Al	0.031	0.714	OTU29	Na	0.049	0.669
OTU450	Al	0.044	-0.680	OTU6	Na	0.007	0.818
OTU21	Al	0.018	0.756	OTU11	Na	0.045	0.678
OTU116	As	0.035	-0.703	OTU13	Na	0.041	0.686
OTU247	As	0.031	-0.713	OTU186	Na	0.031	0.713
OTU170	As	0.046	-0.676	OTU91	Ni	0.044	0.681
OTU30	Ba	0.038	0.695	OTU261	Ni	0.033	0.708
OTU142	Ba	0.011	0.791	OTU791	Ni	0.010	0.799
OTU89	Ba	0.006	0.825	OTU260	Ni	0.049	0.669
OTU342	Ba	0.009	0.801	OTU121	Ni	0.031	0.714
OTU304	Ba	0.018	0.757	OTU134	Ni	0.038	0.695
OTU104	Ca	0.025	0.733	OTU148	Ni	0.011	0.791
OTU267	Ca	0.004	0.850	OTU287	Ni	0.002	0.873
OTU105	Ca	0.007	0.815	OTU165	Ni	0.022	0.743
OTU248	Ca	0.016	0.767	OTU136	Ni	0.019	0.756
OTU272	Ca	0.014	0.776	OTU75	Ni	0.027	0.727
OTU517	Ca	0.023	0.737	OTU450	Ni	0.007	0.817

OTU642	Ca	0.034	0.705	OTU280	Ni	0.007	0.818
OTU512	Ca	0.019	0.755	OTU726	Ni	0.022	0.743
OTU21	Ca	0.026	0.728	OTU268	Ni	0.047	0.673
OTU189	Ca	0.041	-0.687	OTU29	Ni	0.043	-0.682
OTU20	Cd	0.025	0.732	OTU743	Ni	0.027	0.726
OTU231	Cd	0.031	0.714	OTU816	Ni	0.045	0.678
OTU624	Cd	0.013	0.781	OTU700	Ni	0.018	0.758
OTU638	Cd	0.019	0.754	OTU104	Pb	0.023	0.738
OTU102	Ce	0.019	0.753	OTU267	Pb	0.001	0.896
OTU9	Ce	0.021	0.747	OTU105	Pb	0.028	0.721
OTU494	Ce	0.015	0.769	OTU272	Pb	0.026	0.730
OTU450	Ce	0.041	-0.687	OTU517	Pb	0.026	0.730
OTU29	Ce	0.021	0.746	OTU642	Pb	0.037	0.697
OTU7	Ce	0.041	0.688	OTU208	Pb	0.030	0.716
OTU20	Co	0.041	0.688	OTU323	Pb	0.046	0.676
OTU175	Co	0.030	0.717	OTU512	Pb	0.022	0.744
OTU102	Co	0.049	0.669	OTU21	Pb	0.022	0.742
OTU9	Co	0.008	0.814	OTU5	Sb	0.023	0.740
OTU494	Co	0.024	0.735	OTU54	Se	0.046	0.676
OTU29	Co	0.018	0.757	OTU53	Se	0.020	0.751
OTU7	Co	0.015	0.772	OTU89	Se	0.027	0.724
OTU626	Co	0.046	0.675	OTU251	Se	0.045	0.677
OTU79	Cr	0.029	-0.719	OTU183	Se	0.030	0.717
OTU267	Cr	0.023	0.737	OTU157	Se	0.047	0.672
OTU367	Cr	0.050	0.667	OTU421	Se	0.038	0.695
OTU208	Cr	0.030	0.715	OTU166	Se	0.043	0.682
OTU316	Cr	0.033	-0.708	OTU384	Se	0.019	0.754
OTU885	Cr	0.033	-0.708	OTU453	Se	0.019	0.754
OTU104	Cu	0.009	0.803	OTU125	Si	0.049	-0.668
OTU267	Cu	0.024	0.735	OTU267	Si	0.008	0.809
OTU367	Cu	0.050	0.666	OTU208	Si	0.037	0.697
OTU796	Cu	0.019	0.753	OTU147	Si	0.023	-0.739
OTU512	Cu	0.033	0.707	OTU21	Si	0.025	0.731
OTU130	Fe	0.045	-0.677	OTU54	Sn	0.018	0.757
OTU267	Fe	0.024	0.736	OTU88	Sn	0.003	0.853
OTU102	Fe	0.037	0.698	OTU142	Sn	0.003	0.857
OTU494	Fe	0.038	0.693	OTU94	Sn	0.001	0.889
OTU208	Fe	0.042	0.685	OTU138	Sn	0.001	0.897
OTU21	Fe	0.023	0.738	OTU342	Sn	0.000	0.923
OTU33	K	0.019	0.755	OTU149	Sn	0.000	0.920
OTU109	K	0.013	0.779	OTU134	Sn	0.002	0.885
OTU191	K	0.042	0.684	OTU140	Sn	0.001	0.914
OTU201	K	0.014	0.775	OTU148	Sn	0.016	0.768

OTU90	K	0.013	0.781	OTU165	Sn	0.005	0.834
OTU110	K	0.003	0.856	OTU136	Sn	0.006	0.829
OTU220	K	0.037	0.697	OTU280	Sn	0.023	0.739
OTU447	K	0.032	0.710	OTU147	Sn	0.020	0.750
OTU102	K	0.001	0.912	OTU304	Sn	0.008	0.811
OTU340	K	0.017	0.762	OTU267	Sr	0.007	0.816
OTU502	K	0.008	0.808	OTU105	Sr	0.018	0.756
OTU202	K	0.014	0.778	OTU208	Sr	0.035	0.702
OTU494	K	0.004	0.842	OTU647	Sr	0.040	0.689
OTU575	K	0.040	0.688	OTU626	Sr	0.035	0.701
OTU224	K	0.017	0.762	OTU21	Sr	0.014	0.775
OTU437	K	0.005	0.836	OTU130	Ti	0.049	-0.669
OTU178	K	0.029	0.720	OTU102	Ti	0.031	0.712
OTU351	K	0.031	0.712	OTU9	Ti	0.012	0.785
OTU208	K	0.013	0.779	OTU494	Ti	0.026	0.730
OTU533	K	0.028	0.721	OTU450	Ti	0.032	-0.710
OTU556	K	0.010	0.795	OTU29	Ti	0.012	0.785
OTU205	K	0.027	0.726	OTU7	Ti	0.027	0.727
OTU228	K	0.008	0.814	OTU267	V	0.047	0.673
OTU611	K	0.023	0.738	OTU9	V	0.036	0.700
OTU359	K	0.022	0.743	OTU494	V	0.040	0.689
OTU320	K	0.020	0.752	OTU450	V	0.042	-0.686
OTU102	La	0.023	0.738	OTU29	V	0.017	0.760
OTU9	La	0.026	0.730	OTU7	V	0.046	0.675
OTU494	La	0.023	0.740	OTU626	V	0.031	0.715
OTU29	La	0.023	0.740	OTU21	V	0.038	0.694
OTU7	La	0.046	0.674	OTU54	Zn	0.002	0.875
OTU267	Mg	0.041	0.686	OTU88	Zn	0.001	0.894
OTU102	Mg	0.041	0.687	OTU142	Zn	0.001	0.902
OTU9	Mg	0.038	0.693	OTU94	Zn	0.000	0.935
OTU494	Mg	0.025	0.732	OTU89	Zn	0.025	0.731
OTU450	Mg	0.038	-0.695	OTU138	Zn	0.001	0.892
OTU29	Mg	0.028	0.721	OTU342	Zn	0.000	0.947
OTU626	Mg	0.048	0.670	OTU149	Zn	0.000	0.972
OTU21	Mg	0.033	0.706	OTU183	Zn	0.040	0.689
OTU30	Mn	0.048	0.672	OTU134	Zn	0.001	0.903
OTU267	Mn	0.004	0.846	OTU140	Zn	0.000	0.951
OTU105	Mn	0.020	0.749	OTU148	Zn	0.012	0.787
OTU208	Mn	0.022	0.743	OTU165	Zn	0.004	0.845
OTU170	Mn	0.044	-0.681	OTU136	Zn	0.004	0.851
OTU21	Mn	0.013	0.781	OTU280	Zn	0.016	0.765
OTU54	Mo	0.022	0.741	OTU147	Zn	0.011	0.793
OTU88	Mo	0.021	0.745	OTU304	Zn	0.006	0.827

OTU142	Mo	0.009	0.803	OTU453	Zn	0.049	0.667
OTU94	Mo	0.001	0.904	OTU20	Zr	0.001	0.901
OTU138	Mo	0.008	0.809	OTU2	Zr	0.036	0.699
OTU342	Mo	0.001	0.894	OTU62	Zr	0.041	0.687
OTU149	Mo	0.003	0.859	OTU144	Zr	0.001	0.909
OTU134	Mo	0.000	0.981	OTU175	Zr	0.001	0.916
OTU140	Mo	0.000	0.975	OTU9	Zr	0.016	0.768
OTU148	Mo	0.000	0.921	OTU231	Zr	0.003	0.858
OTU165	Mo	0.000	0.971	OTU624	Zr	0.019	0.755
OTU136	Mo	0.000	0.963	OTU623	Zr	0.025	0.732
OTU280	Mo	0.000	0.919	OTU29	Zr	0.039	0.691
OTU147	Mo	0.035	0.703	OTU22	Zr	0.029	0.718
OTU304	Mo	0.023	0.738	OTU7	Zr	0.006	0.831
OTU1	Na	0.011	0.793	OTU68	Zr	0.031	0.712
OTU2	Na	0.049	0.670	OTU143	Zr	0.039	0.691
OTU9	Na	0.017	0.760	OTU12	Zr	0.042	0.684
			0.822	OTU82	Zr	0.008	0.811

Section S16. Incomplete list of potentially pathogenic bacteria and fungi identified.

SI Table S10. Incomplete list of potentially pathogenic bacteria and fungi measured in our study.

Species	Average Abundance	Impact	Description
**Bacteria**			
Escherichia coli	0.76%	Animal<br>Human	Avian pathogen<br>Human intestinal pathogen
Propionibacterium acnes	0.16%	Human	Opportunistic: acne vulgaris, endocarditis, endophthalmitis, prosthetic joint infections
Roseomonas mucosa	0.05%	Human	Opportunistic/nosocomial infector: peritonitis, bacteremia, endocarditis, endophthalmitis
Haemophilus parainfluenzae	0.02%	Human	Opportunistic: upper respiratory tract infections, urogenital infections, gastroenteritis, endocarditis
**Fungi**			
Fomes fasciatus	1.38%	Plant	Wood decay
Funalia floccosa	1.14%	Plant	White rot
Curvularia lunata	1.03%	Plant<br>Human	Tomato early blight, leaf spot<br>Phaeohyphomycosis
Tropicoporus tropicalis	0.49%	Plant<br>Human	White rot<br>Keratitis, Mycosis
Schizophyllum commune	0.38%	Plant<br>Human	Wood rot<br>Lung infection
Choanephora cucurbitarum	0.37%	Plant	Seedling rot, blight, fruit rot, leaf wilt, flower rot, stem necrosis, leaf spot
Rhizopus microsporus	0.33%	Human	Rhinocerebral mucormycosis, lung infection
Aspergillus fumigatus	0.29%	Human<br>Animal	Aspergillosis<br>Aspergillosis
Penicillium sclerotiorum	0.26%	Plant	Leaf spot, post-harvest decay
Phellinus gilvus	0.17%	Plant	Wood decay
Exserohilum rostratum	0.15%	Plant<br>Human	Leaf spot, rice brown spot, lettuce root rot<br>Sinusitis, Keratitis
Stemphylium herbarum	<0.1%	Plant	Brown spot, leaf spot
Penicillium sumatraense	<0.1%	Plant	Post-harvest decay
Aureobasidium pullulans	<0.1%	Human	Opportunistic: subcutaneous phaeohyphomycosis, fungemia, peritonitis, pneumonia
Fusarium oxysporum	<0.1%	Plant<br>Human	Stem rot, wilt, dieback<br>Fusariosis
Phanerochaete chrysosporium	<0.1%	Plant	White rot
Amphobotrys ricini	<0.1%	Plant	Gray mold

Penicllium georgiense	<0.1%	Plant	Post-harvest decay
Curvularia trifolii	<0.1%	Plant	Blight, leaf spot
Cladosporium sphaerospermum	<0.1%	Plant	Leaf spot
		Human	Opportunistic: abscesses, cutaneous infections
Tilletia barclayana	<0.1%	Plant	Rice/grain smut
Hyphodermella rosae	<0.1%	Plant	White rot, dry fruit rot
Nigrospora oryzae	<0.1%	Plant	Leaf spot, leaf blight
Aurantiporus fissilis	<0.1%	Plant	Wood decay
Purpureocillium lilacinum	<0.1%	Human	Opportunistic: hyalohyphomycosis, ocular infections
		Animal	Nematicide
Wallemia sebi	<0.1%	Human	Subcutaneous phaeohyphomycosis
Trichothecium roseum	<0.1%	Plant	Fruit rot, post-harvest decay, root rot, dieback, leaf spot
Periconia macrospinosa	<0.1%	Plant	Leaf necrosis
Aspergillus penicillioides	<0.1%	Human	Opportunistic: subcutaneous infections, keratomycosis, aspergillosis
Pilidium concavum	<0.1%	Plant	Tan-brown rot, fruit rot, leaf necrosis
Saccharomyces cerevisiae	<0.1%	Human	Opportunistic in immunocompromised patients: vaginitis, blood stream infections, essential organ infections
Eutypa leptoplaca	<0.1%	Plant	Grapevine pathologies; dieback
Rhizopus arrhizus	<0.1%	Plant	Root rot, soft rot, post-harvest rot
		Human	Rhinosinusitis mucormycosis
Favolus grammocephalus	<0.1%	Plant	White rot
Biatriospora mackinnonii	<0.1%	Human	Eumycetoma, cutaneous phaeohyphomycosis
Gjaerumia minor	<0.1%	Human	Keratitis
Antrodia pini-cubensis	<0.1%	Plant	Brown rot
Trametes versicolor	<0.1%	Plant	White rot
Punctularia strigosozonata	<0.1%	Plant	White rot
Tolypocladium inflatum	<0.1%	Animal	Insecticidal
Monocillium indicum	<0.1%	Animal	Lymphadenitis, splenitis
Quambalaria cyanscens	<0.1%	Plant	Smut
Cordyceps bassiana	<0.1%	Animal	Insecticidal
Trichaptum abietinum	<0.1%	Plant	Wood decay
Thermomyces lanuginosus	<0.1%	Human	Opportunistic: endocarditis
Malassezia restricta	<0.1%	Human	Opportunistic: endocarditis, pneumonia, dermatitis
Bjerkandera adusta	<0.1%	Human	Bronchopulmonary mycosis
Waitea circinata var. circinata	<0.1%	Plant	Brown ring patch
Phlebia chrysocreas	<0.1%	Plant	Heartrot
Neopestalotiopsis foedans	<0.1%	Plant	Leaf spot
Phlebiopsis gigantea	<0.1%	Plant	Wood decay
Ceratobasidium ramicola	<0.1%	Plant	Dieback, leaf blight, leaf rot, damping off, root rot

Rigidoporus ulmarius	<0.1%	Plant	White rot
Postia caesia	<0.1%	Plant	Brown rot
Exidia glandulosa	<0.1%	Plant	Wood decay
Botryosphaeria dothidea	<0.1%	Plant	Blueberry canker, brown rot, pear ring rot, kiwi soft rot
Gonatobotryum apiculatum	<0.1%	Plant	Leaf spot, blight
Fibroporia radiculosa	<0.1%	Plant	Wood decay, brown rot
Daldinia eschscholtzii	<0.1%	Plant	Wood decay
Gloeophyllum trabeum	<0.1%	Plant	Wood decay, brown rot
Thanatephorus cucumeris	<0.1%	Plant	Rice sheath blight, tomato seedling damping off, potato black scurf, stem canker, root rot
Phaeoacremonium croatiense	<0.1%	Plant	Citrus tree diseases

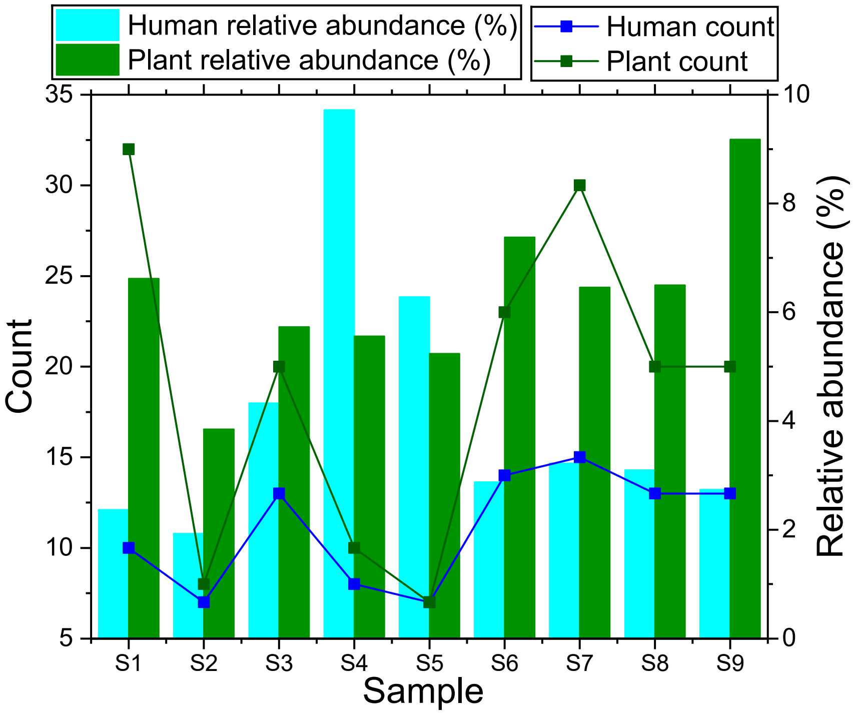

SI Figure S23. Relative abundance and presence/absence of fungal pathogens affecting plants and humans across the sampling period.

The number of unique pathogens present in each sample and the cumulative abundance of each type of pathogen are summarized in the following plot, incorporating data from SI Table S10 and relative abundance data previously discussed in this manuscript. Plant pathogen abundance was elevated on S1 and from S6 to S9, the latter mostly due to a spike in *Fomes fasciatus*, and the number of unique plant pathogens present was highest in S1 and S7. The period corresponding with the height of the dust event appears to have lessened presence and abundance of plant pathogens, but there was an increase in both

measures from pre-peak sample S2 to S3. Inversely, human pathogen abundance was elevated on S4 and S5 and lowest in pre-peak samples S1 and S2. The number of unique human pathogens was greatest in S6 through S9. *Curvularia lunata*, *Tropicoporus tropicalis*, and *Rhizopus microsporus* were the main drivers of the abundance spike in S4 and S5. Repeated observations of the baseline and African dust events are needed to better interpret such trends. As this paper is more of an observational report, we prefer not to overstate these trends as direct effects of Saharan dust intrusion.

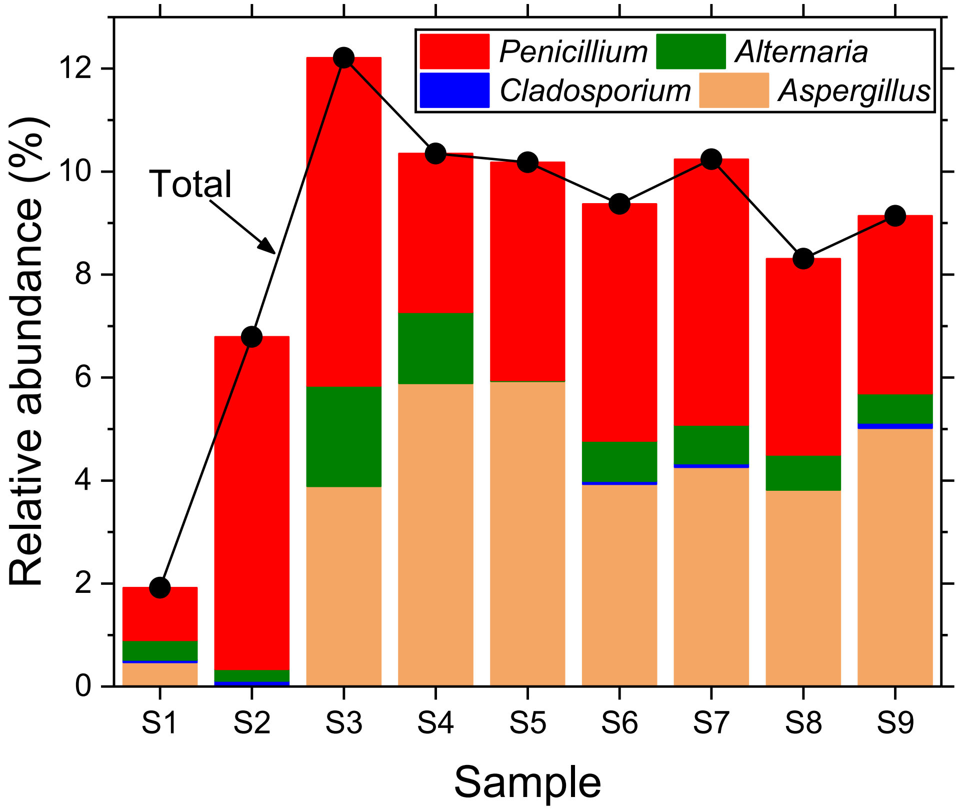

SI Figure S24. Relative abundance of the four fungal genera that are well-known for allergenic effects.

SI Table S11. Summary of Pagel's λ values for the entire dataset and corresponding p values.

Sample	Fungi		Bacteria	
	lambda	p	lambda	p
S1	0.34	<0.01	0.00	NS
S2	0.13	<0.01	0.16	<0.01
S3	0.28	<0.01	0.11	NS
S4	0.22	<0.01	0.08	NS
S5	0.19	<0.01	0.08	<0.01
S6	0.25	<0.01	0.11	<0.01
S7	0.23	<0.01	0.06	NS
S8	0.36	<0.01	0.26	<0.01
S9	0.26	<0.01	0.17	<0.01

Section S16. Bioinformatics analysis of bacterial diversity

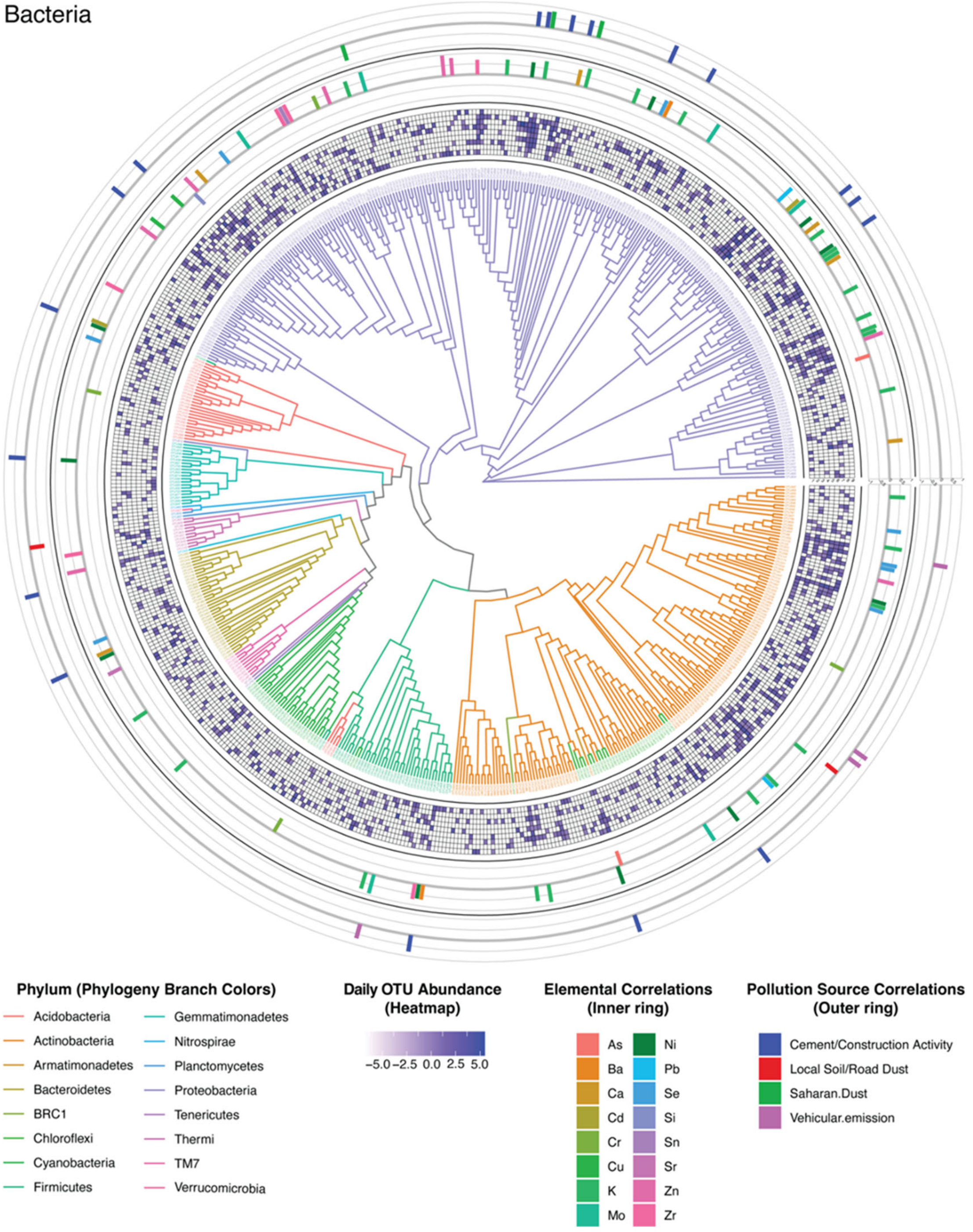

SI Figure S25. From inner most ring to outermost: Bacterial phylogenetic tree; Bacterial OTU heatmap (2nd from center) indicating relative abundances of each OTU from samples S1 (inner) to S9 (outer); Positive (pointing outward) and negative (pointing inward) correlations between elemental concentrations and bacterial OTU variations (3rd from center); Positive (pointing outward) and negative (pointing inward) correlations between aerosol pollution source and bacterial OTU variations (outermost). Some OTUs were significantly correlated to more than one element or source, in which case only the strongest correlations are visualized here. SI Tables S8 and S9 summarize all significant correlations.

Section S17. Additional statements of novelty and relevance to environmental science

Whereas African dust has been evaluated as a source of microorganisms in Europe (including Canary Islands and Israel) [52, 71, 79-84] and the Caribbean [85-87] we, for the first time, comprehensively investigated its role in aerosol elemental composition and microbial community structure at a populous industrialized metroplex in the North American mainland. Our measurements of 46 representative and (inner)transition elements coupled with bacterial and fungal diversity add to our knowledge of aerosol health effects, long-range microbial dispersal [88] and further informs epidemiological links drawn between Saharan dust and morbidity and mortality close to its source in Europe [79, 81, 89-92] and sub-Saharan Africa [93]. Elemental measurements quantitatively captured Saharan-Sahelian contributions to ambient PM10 mass and sensitively tracked its ground-level mixing with locally-sourced aerosols [2, 3, 25].

We would like to emphasize that this study is unique and novel on at least two fronts: (i) it is the very first dataset of its kind combining detailed elemental and genomic measurements coincident with a Saharan dust event in North America and (ii) we report both 16S rDNA and 18S/ITS sequencing, i.e., information on bacteria and fungi, respectively. Crucially, since culturable microbes represent $< 1\%$ of the total microbial population, we employed next-generation sequencing (NGS) to obtain a complete taxonomic profile of microbial communities (both bacteria and fungi). Hence, this is the first comprehensive report of the metals, prokaryotes, and eukaryotes associated with urban aerosols mixed with North African dust. We detected numerous human pathogens on the World Health Organization's priority list along with multiple plant pathogens demonstrating a new aspect to aerosol health effects. Furthermore, studies such as ours have been identified as an important gap and research need in the literature [80, 90, 91, 94]. Importantly, our research has broader implications for environmental science and engineering, long-distance dispersal of microorganisms, public health, and the planet's radiation budget , climate, etc.

The proposed mechanism being tested is simply that microorganisms are being carried along with windblown dust all the way from Africa, across the Atlantic Ocean and the Gulf of Mexico to Houston. This has been shown to occur at more local scales [80, 94-97] including locations close to the Saharan and Arabian deserts, [54, 82, 83, 98-101] and in global scales [102-105]. However, it is not well-established whether microbiota originating from North Africa are able to disperse across the Atlantic Ocean during dust storm events and if so, to what extent and what is their genetic diversity (both prokaryotes and eukaryotes). While very limited information is available using conventional plating methods from remote locations, our study is the first to examine these questions in detail using state-of-the-art techniques and in a metroplex where more than 7 million people are exposed to dust and its components. We emphasize that culturable microbes represent < 1% of the total microbial population. Hence, we used next-generation sequencing (NGS) to obtain a complete taxonomic profile of microbial communities. We show for the first time that the abundance of some microbiota increases with the mass concentration of Saharan dust that has migrated across the Atlantic and that changes in overall community compositions also occur in concert with changes in Saharan dust concentrations. However, we were unable to find direct quantitative evidence with our dataset that microbiota are actually being carried with the dust. Subsequent studies building off the framework established here, comparing source and destination microbiomes, are planned.

Section S18. Principal Coordinate Analyses (PCoA) plots

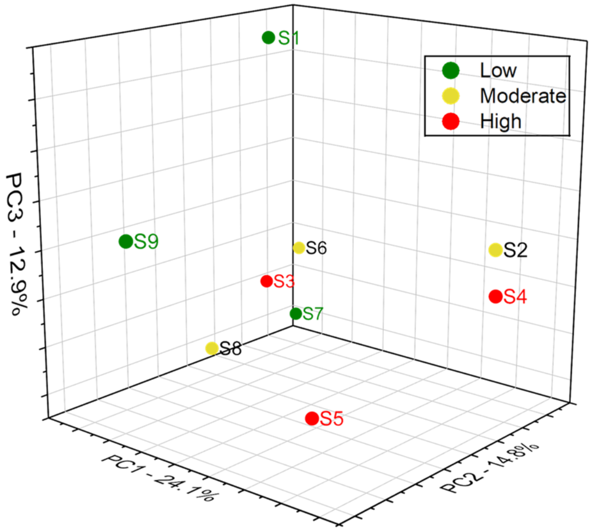

SI Figure S26a. Three dimensional principal coordinate analyses (PCoA) of bacterial communities. Each sample is labelled with its corresponding number shown in Table 1. Samples are also categorized by North African dust content; red dots correspond to high estimated contributions (> 40 µg/m³ or peak samples, S3, S4, and S5), orange dots correspond to African dust mass between 10 and 40 µg/m³ (samples S2, S6, and S8), and green symbols represent low African contributions (< 10 µg/m³, samples collected at the beginning and end of the episode, S1, S7, and S9).

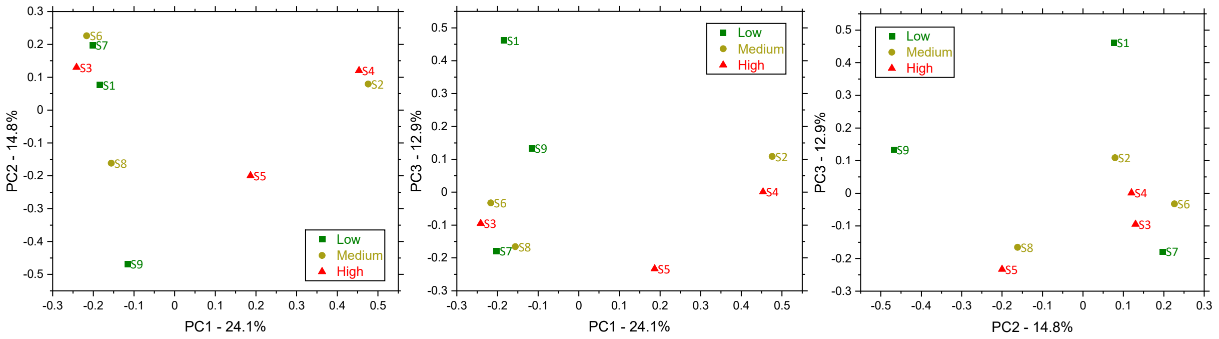

SI Figure S26b. Two dimensional principal coordinate analyses (PCoA) of bacterial communities. The data used for these figures are identical to the figure above (SI Figure S26a). They are depicted in 2-D herein because that may be a better visual representation of clustering, which is sometimes difficult to discern in a 3-D representation.

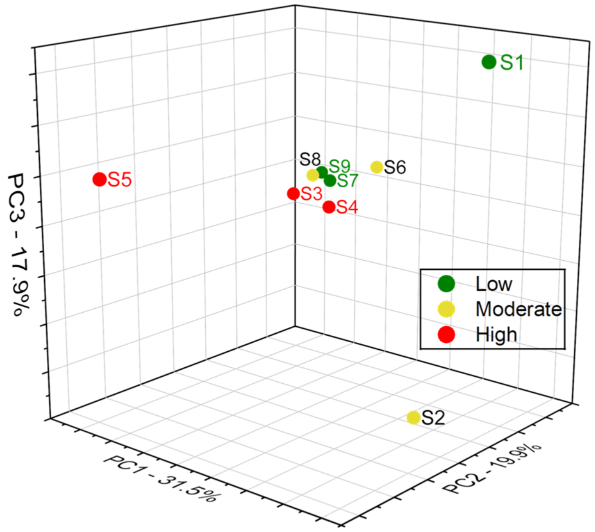

SI Figure S27a. Three dimensional principal coordinate analyses (PCoA) of fungal communities. Each sample is labelled with its corresponding number shown in Table 1. Samples are also categorized by North African dust content; red dots correspond to high estimated contributions (> 40 µg/m^3 or peak samples, S3, S4, and S5), orange dots correspond to African dust mass between 10 and 40 µg/m^3 (samples S2, S6, and S8), and green symbols represent low African contributions (< 10 µg/m^3, samples collected at the beginning and end of the episode, S1, S7, and S9).

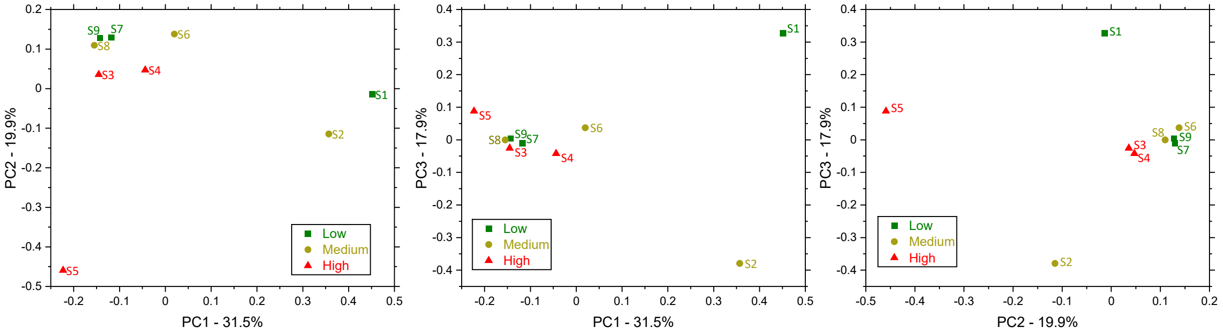

SI Figure S27b. Two dimensional principal coordinate analyses (PCoA) of fungal communities. The data used for these figures are identical to the figure above (SI Figure S27a). They are depicted in 2-D herein because that may be a better visual representation of clustering, which is sometimes difficult to discern in a 3-D representation.

REFERENCES

1. Sullivan, D.W., J.H. Price, B. Lambeth, K.A. Sheedy, K. Savanich, and R.J. Tropp, *Field study and source attribution for $PM_{2.5}$ and PM_{10} with resulting reduction in concentrations in the neighborhood north of the Houston Ship Channel based on voluntary efforts.* Journal of the Air & Waste Management Association, 2013. **63**(9): p. 1070-1082.
2. Bozlaker, A., J.M. Prospero, J. Price, and S. Chellam, *Identifying and Quantifying the Impacts of Advected North African Dust on the Concentration and Composition of Airborne Fine Particulate Matter in Houston and Galveston, Texas.* Journal of Geophysical Research: Atmospheres, 2019. **124**(22): p. 12282-12300.
3. Bozlaker, A., J.M. Prospero, M.P. Fraser, and S. Chellam, *Quantifying the contribution of long-range Saharan Dust transport on particulate matter concentrations in Houston, Texas, using detailed elemental analysis.* Environmental Science & Technology, 2013. **47**(18): p. 10179-10187.
4. Kulkarni, P., S. Chellam, and M.P. Fraser, *Tracking petroleum refinery emission events using Lanthanum and Lanthanides as elemental markers for $PM_{2.5}$.* Environmental Science & Technology, 2007. **41**(19): p. 6748-6754.
5. Bozlaker, A., J.M. Prospero, J. Price, and S. Chellam, *Linking Barbados mineral dust aerosols to North African sources using elemental composition and radiogenic Sr, Nd, and Pb Isotope signatures.* Journal of Geophysical Research: Atmospheres, 2018. **123**(2): p. 1384-1400.
6. Das, S., B.V. Miller, J. Prospero, and S. Chellam, *Sr-Nd-Hf isotopic analysis of reference materials and natural and anthropogenic particulate matter sources: Implications for accurately tracing North African dust in complex urban atmospheres.* Talanta, 2022. **241**: p. 123236.
7. Danadurai, K.S.K., S. Chellam, C.T. Lee, and M.P. Fraser, *Trace elemental analysis of airborne particulate matter using dynamic reaction cell inductively coupled plasma - mass spectrometry: application to monitoring episodic industrial emission events.* Analytica Chimica Acta, 2011. **686**(1-2): p. 40-49.
8. Bozlaker, A., B. Buzcu-Guven, M.P. Fraser, and S. Chellam, *Insights into PM_{10} sources in Houston, Texas: Role of petroleum refineries in enriching lanthanoid metals during episodic emission events.* Atmospheric Environment, 2013. **69**: p. 109-117.
9. NASA World View. *NASA World View*. 2017 06 Feb 2022]; Available from: https://worldview.earthdata.nasa.gov/.
10. Hogan, T.F. and T.E. Rosmond, *The description of the Navy Operational Global Atmospheric Prediction System's spectral forecast model.* Monthly Weather Review, 1991. **119**(8): p. 1786-1815.
11. Draxler, R.R. and G.D. Rolph. *HYSPLIT (HYbrid Single-Particle Lagrangian Integrated Trajectory) model*. 2010; Available from: https://www.ready.noaa.gov/HYSPLIT.php.
12. Stein, A.F., R.R. Draxler, G.D. Rolph, B.J.B. Stunder, M.D. Cohen, and F. Ngan, *NOAA's HYSPLIT atmospheric transport and dispersion modeling system.* Bulletin of the American Meteorological Society, 2015. **96**(12): p. 2059-2077.
13. NASA. *Global Modeling and Assimilation Office (GMAO) Framework for Live User-Invoked Data (FLUID) - Atmospheric composition (2D) maps*. 2018; Available from: https://fluid.nccs.nasa.gov/wxmaps/chem2d/.
14. Watson, J.G., N.F. Robinson, J.C. Chow, R.C. Henry, B.M. Kim, T.G. Pace, E.L. Meyer, and Q. Nguyen, *The USEPA/DRI chemical mass balance receptor model, CMB 7.0.* Environmental Software, 1990. **5**(1): p. 38-49.

15. Das, S. and S. Chellam, *Estimating light-duty vehicles' contributions to ambient $PM_{2.5}$ and PM_{10} at a near-highway urban elementary school via elemental characterization emphasizing Rhodium, Palladium, and Platinum.* Science of the Total Environment, 2020. **747**: p. 141268.
16. Bozlaker, A., N.J. Spada, M.P. Fraser, and S. Chellam, *Elemental characterization of $PM_{2.5}$ and PM_{10} emitted from light duty vehicles in the Washburn tunnel of Houston, Texas: Release of rhodium, palladium, and platinum.* Environmental Science & Technology, 2014. **48**(1): p. 54-62.
17. Reff, A., P.V. Bhave, H. Simon, T.G. Pace, G.A. Pouliot, J.D. Mobley, and M. Houyoux, *Emissions inventory of $PM_{2.5}$ trace elements across the United States.* Environmental Science & Technology, 2009. **43**(15): p. 5790-5796.
18. Moreno, T., X. Querol, A. Alastuey, J. de la Rosa, A.M. Sánchez de la Campa, M. Minguillón, M. Pandolfi, Y. González-Castanedo, E. Monfort, and W. Gibbons, *Variations in vanadium, nickel and lanthanoid element concentrations in urban air.* Science of the Total Environment, 2010. **408**(20): p. 4569-4579.
19. Kulkarni, P., S. Chellam, and M.P. Fraser, *Lanthanum and lanthanides in atmospheric fine particles and their apportionment to refinery and petrochemical operations in Houston, TX.* Atmospheric Environment, 2006. **40**(3): p. 508-520.
20. Bakker, N.L., N.A. Drake, and C.S. Bristow, *Evaluating the relative importance of northern African mineral dust sources using remote sensing.* Atmos. Chem. Phys., 2019. **19**(16): p. 10525-10535.
21. Formenti, P., S. Caquineau, K. Desboeufs, A. Klaver, S. Chevaillier, E. Journet, and J.L. Rajot, *Mapping the physico-chemical properties of mineral dust in western Africa: mineralogical composition.* Atmos. Chem. Phys., 2014. **14**(19): p. 10663-10686.
22. Formenti, P., L. Schütz, Y. Balkanski, K. Desboeufs, M. Ebert, K. Kandler, A. Petzold, D. Scheuvens, S. Weinbruch, and D. Zhang, *Recent progress in understanding physical and chemical properties of African and Asian mineral dust.* Atmos. Chem. Phys., 2011. **11**(16): p. 8231-8256.
23. Guinoiseau, D., S.P. Singh, S.J.G. Galer, W. Abouchami, R. Bhattacharyya, K. Kandler, C. Bristow, and M.O. Andreae, *Characterization of Saharan and Sahelian dust sources based on geochemical and radiogenic isotope signatures.* Quaternary Science Reviews, 2022. **293**: p. 107729.
24. Jewell, A.M., N. Drake, A.J. Crocker, N.L. Bakker, T. Kunkelova, C.S. Bristow, M.J. Cooper, J.A. Milton, P.S. Breeze, and P.A. Wilson, *Three North African dust source areas and their geochemical fingerprint.* Earth and Planetary Science Letters, 2021. **554**: p. 116645.
25. Das, S., B.V. Miller, J.M. Prospero, C.J. Gaston, H.M. Royer, E. Blades, P. Sealy, and S. Chellam, *Coupling Sr–Nd–Hf Isotope Ratios and Elemental Analysis to Accurately Quantify North African Dust Contributions to $PM_{2.5}$ in a Complex Urban Atmosphere by Reducing Mineral Dust Collinearity.* Environmental Science & Technology, 2022. **56**(12): p. 7729-7740.
26. Rudnick, R.L. and S. Gao, *Composition of the continental crust*, in *Treatise on Geochemistry*, H.D. Holland and K.K. Turekian, Editors. 2003, Elsevier-Pergamon: Oxford. p. 1-64.
27. Moreno, T., X. Querol, A. Alastuey, and W. Gibbons, *Identification of FCC refinery atmospheric pollution events using lanthanoid- and vanadium-bearing aerosols.* Atmospheric Environment, 2008. **42**(34): p. 7851-7861.
28. Williamson, K., S. Das, A.R. Ferro, and S. Chellam, *Elemental composition of indoor and outdoor coarse particulate matter at an inner-city high school.* Atmospheric Environment, 2021. **261**: p. 118559.
29. TCEQ-Forecast. *TCEQ daily air quality forecast*. Available from: https://www.tceq.texas.gov/airquality/monops/forecast_today.html (Accessed 30 January 2022).
30. Bozlaker, A., B. Buzcu-Güven, M.P. Fraser, and S. Chellam, *Insights into PM10 sources in Houston, Texas: Role of petroleum refineries in enriching lanthanoid metals during episodic emission events.* Atmospheric Environment, 2013. **69**: p. 109-117.

31. Sullivan, D.W., J.H. Price, B. Lambeth, K.A. Sheedy, K. Savanich, and R.J. Tropp, *Field study and source attribution for PM2.5 and PM10 with resulting reduction in concentrations in the neighborhood north of the Houston Ship Channel based on voluntary efforts.* Journal of the Air and Waste Management Association 2013. **63**(9): p. 1070-82.
32. Han, I., D. Richner, H. An Han, L. Hopkins, D. James, and E. Symanski, *Evaluation of metal aerosols in four communities adjacent to metal recyclers in Houston, Texas, USA.* J Air Waste Manag Assoc, 2020. **70**(5): p. 568-579.
33. Olmez, I. and G.E. Gordon, *Rare earths: Atmospheric signatures for oil-fired power plants and refineries.* Science, 1985. **229**(4717): p. 966-968.
34. Moreno, T., N. Perez, X. Querol, F. Amato, A. Alastuey, R. Bhatia, B. Spiro, M. Hanvey, and W. Gibbons, *Physicochemical variations in atmospheric aerosols recorded at sea onboard the Atlantic-Mediterranean 2008 Scholar Ship cruise (Part II): Natural versus anthropogenic influences revealed by PM_{10} trace element geochemistry.* Atmospheric Environment, 2010. **44**: p. 2563-2576.
35. Moreno, T., X. Querol, A. Alastuey, J. de la Rosa, A.M.S. de la Campa, M. Minguillon, M. Pandolfi, Y. González-Castanedo, E. Monfort, and W. Gibbons, *Variations in vanadium, nickel and lanthanoid element concentrations in urban air.* Science of the Total Environment, 2010. **408**: p. 4569–4579.
36. Celo, V., E. Dabek-Zlotorzynska, and M. McCurdy, *Chemical characterization of exhaust emissions from selected Canadian marine vessels: The case of trace metals and lanthanoids.* Environmental Science and Technology, 2015. **49**(8): p. 5220-5226.
37. Abouchami, W., K. Nathe, A. Kumar, S.J.G. Galer, K.P. Jochum, E. Williams, A.M.C. Horbe, J.W.C. Rosa, W. Balsam, D. Adams, K. Mezger, and M.O. Andreae, *Geochemical and isotopic characterization of the Bodele Depression dust source and implications for transatlantic dust transport to the Amazon Basin.* Earth and Planetary Science Letters, 2013. **380**: p. 112-123.
38. Zhao, W., W. Balsam, E. Williams, X. Long, and J. Ji, *Sr–Nd–Hf isotopic fingerprinting of transatlantic dust derived from North Africa.* Earth and Planetary Science Letters, 2018. **486**: p. 23-31.
39. Scheuvens, D., L. Schütz, K. Kandler, M. Ebert, and S. Weinbruch, *Bulk composition of northern African dust and its source sediments — A compilation.* Earth-Science Reviews, 2013. **116**: p. 170-194.
40. Castillo, S., *Trace element variation in size-fractionated African desert dusts.* Journal of Arid Environments, 2008. **72**(6): p. 1034-1045.
41. Spada, N., A. Bozlaker, and S. Chellam, *Multi-elemental characterization of tunnel and road dusts in Houston, Texas using dynamic reaction cell-quadrupole-inductively coupled plasma–mass spectrometry: Evidence for the release of platinum group and anthropogenic metals from motor vehicles.* Analytica Chimica Acta, 2012. **735**: p. 1-8.
42. Gaschnig, R.M., R.L. Rudnick, W.F. McDonough, A.J. Kaufman, J.W. Valley, Z. Hu, S. Gao, and M.L. Beck, *Compositional evolution of the upper continental crust through time, as constrained by ancient glacial diamictites.* Geochimica et Cosmochimica Acta, 2016. **186**: p. 316-343.
43. Dillner, A.M., J.J. Schauer, W.F. Christensen, and G.R. Cass, *A quantitative method for clustering size distributions of elements.* Atmospheric Environment, 2005. **39**(8): p. 1525-1537.
44. Querol, X., N. Perez, C. Reche, M. Ealo, A. Ripoll, J. Tur, M. Pandolfi, J. Pey, P. Salvador, T. Moreno, and A. Alastuey, *African dust and air quality over Spain: Is it only dust that matters?* Science of the Total Environment, 2019. **686**: p. 737-752.
45. Moreno, T., N. Perez, X. Querol, F. Amato, A. Alastuey, R. Bhatia, B. Spiro, M. Hanvey, and W. Gibbons, *Physicochemical variations in atmospheric aerosols recorded at sea onboard the Atlantic-Mediterranean 2008 Scholar Ship cruise (Part II): Natural versus anthropogenic*

influences revealed by PM_{10} trace element geochemistry. Atmospheric Environment, 2010. **44**(21-22): p. 2563-2576.
46. Du, L. and J. Turner, *Using $PM_{2.5}$ lanthanoid elements and nonparametric wind regression to track petroleum refinery FCC emissions.* Science of the Total Environment, 2015. **529**: p. 65-71.
47. Chen, L.W.A., J.G. Watson, J.C. Chow, D.W. DuBois, and L. Herschberger, *Chemical mass balance source apportionment for combined $PM_{2.5}$ measurements from US non-urban and urban long-term networks.* Atmospheric Environment, 2010. **44**(38): p. 4908-4918.
48. Marconi, M., D.M. Sferlazzo, S. Becagli, C. Bommarito, G. Calzolai, M. Chiari, A. di Sarra, C. Ghedini, J.L. Gómez-Amo, F. Lucarelli, D. Meloni, F. Monteleone, S. Nava, G. Pace, S. Piacentino, F. Rugi, M. Severi, R. Traversi, and R. Udisti, *Saharan dust aerosol over the central Mediterranean Sea: PM_{10} chemical composition and concentration versus optical columnar measurements.* Atmospheric Chemistry and Physics, 2014. **14**(4): p. 2039-2054.
49. López-Aladid, R., L. Fernández-Barat, V. Alcaraz-Serrano, L. Bueno-Freire, N. Vázquez, R. Pastor-Ibáñez, A. Palomeque, P. Oscanoa, and A. Torres, *Determining the most accurate 16S rRNA hypervariable region for taxonomic identification from respiratory samples.* Scientific Reports, 2023. **13**(1): p. 3974.
50. Chakravorty, S., D. Helb, M. Burday, N. Connell, and D. Alland, *A detailed analysis of 16S ribosomal RNA gene segments for the diagnosis of pathogenic bacteria.* Journal of Microbiological Methods, 2007. **69**(2): p. 330-339.
51. Romano, S., M. Di Salvo, G. Rispoli, P. Alifano, M.R. Perrone, and A. Talà, *Airborne bacteria in the Central Mediterranean: Structure and role of meteorology and air mass transport.* Science of The Total Environment, 2019. **697**: p. 134020.
52. Gonzalez-Martin, C., C.J. Perez-Gonzalez, E. Gonzalez-Toril, F.J. Exposito, A. Aguilera, and J.P. Diaz, *Airborne Bacterial Community Composition According to Their Origin in Tenerife, Canary Islands.* Frontiers in Microbiology, 2021. **12**: p. 732961.
53. Gonzalez-Toril, E., S. Osuna, D. Viudez-Moreiras, I. Navarro-Cid, S.D.D. Toro, S. Sor, R. Bardera, F. Puente-Sanchez, G. de Diego-Castilla, and A. Aguilera, *Impacts of Saharan Dust Intrusions on Bacterial Communities of the Low Troposphere.* Scientific Reports, 2020. **10**(1): p. 6837.
54. Iakovides, M., G. Tsiamis, T. Tziaras, P. Stathopoulou, S. Nikolaki, G. Iakovides, and E.G. Stephanou, *Two-year systematic investigation reveals alterations induced on chemical and bacteriome profile of $PM_{2.5}$ by African dust incursions to the Mediterranean atmosphere.* Science of the Total Environment, 2022. **815**: p. 151976.
55. Caporaso, J.G., J. Kuczynski, J. Stombaugh, K. Bittinger, F.D. Bushman, E.K. Costello, N. Fierer, A.G. Peña, J.K. Goodrich, J.I. Gordon, G.A. Huttley, S.T. Kelley, D. Knights, J.E. Koenig, R.E. Ley, C.A. Lozupone, D. McDonald, B.D. Muegge, M. Pirrung, J. Reeder, J.R. Sevinsky, P.J. Turnbaugh, W.A. Walters, J. Widmann, T. Yatsunenko, J. Zaneveld, and R. Knight, *QIIME allows analysis of high-throughput community sequencing data.* Nature Methods, 2010. **7**(5): p. 335-336.
56. Rognes, T., T. Flouri, B. Nichols, C. Quince, and F. Mahé, *VSEARCH: a versatile open source tool for metagenomics.* PeerJ, 2016. **4**: p. e2584.
57. Quast, C., E. Pruesse, P. Yilmaz, J. Gerken, T. Schweer, P. Yarza, J. Peplies, and F.O. Glöckner, *The SILVA ribosomal RNA gene database project: improved data processing and web-based tools.* Nucleic Acids Research, 2013. **41**(Database issue): p. D590-6.
58. Kõljalg, U., H.R. Nilsson, D. Schigel, L. Tedersoo, K.H. Larsson, T.W. May, A.F.S. Taylor, T.S. Jeppesen, T.G. Frøslev, B.D. Lindahl, K. Põldmaa, I. Saar, A. Suija, A. Savchenko, I. Yatsiuk, K. Adojaan, F. Ivanov, T. Piirmann, R. Pöhönen, A. Zirk, and K. Abarenkov, *The Taxon Hypothesis Paradigm-On the Unambiguous Detection and Communication of Taxa.* Microorganisms, 2020. **8**(12): p. 1910.

59. Mazar, Y., E. Cytryn, Y. Erel, and Y. Rudich, *Effect of Dust Storms on the Atmospheric Microbiome in the Eastern Mediterranean.* Environmental Science & Technology, 2016. **50**(8): p. 4194-202.
60. Petroselli, C., E. Montalbani, G. La Porta, S. Crocchianti, B. Moroni, C. Casagrande, E. Ceci, R. Selvaggi, B. Sebastiani, I. Gandolfi, A. Franzetti, E. Federici, and D. Cappelletti, *Characterization of long-range transported bioaerosols in the Central Mediterranean.* Science of the Total Environment, 2021. **763**: p. 143010.
61. Qi, Y., Y. Li, W. Xie, R. Lu, F. Mu, W. Bai, and S. Du, *Temporal-spatial variations of fungal composition in $PM_{2.5}$ and source tracking of airborne fungi in mountainous and urban regions.* Science of The Total Environment, 2020. **708**: p. 135027.
62. Stern, R.A., N. Mahmoudi, C.O. Buckee, A.T. Schartup, P. Koutrakis, S.T. Ferguson, J.M. Wolfson, S.C. Wofsy, B.C. Daube, and E.M. Sunderland, *The Microbiome of Size-Fractionated Airborne Particles from the Sahara Region.* Environmental Science & Technology, 2021. **55**(3): p. 1487-1496.
63. Legendre, P. and L.F.J. Legendre, *Numerical Ecology*. 2nd Edition ed. 1998: Elsevier, Amsterdam. 852.
64. Fitzpatrick, M., K. Mokany, G. Manion, D. Nieto-Lugilde, and S. Ferrier, *gdm: Generalized Dissimilarity Modeling. R package version 1.5.0-9.1*. 2022: https://CRAN.R-project.org/package=gdm.
65. Ferrier, S., G. Manion, J. Elith, and K. Richardson, *Using generalized dissimilarity modelling to analyse and predict patterns of beta diversity in regional biodiversity assessment.* Diversity and Distributions, 2007. **13**(3): p. 252-264.
66. Fitzpatrick, M.C. and S.R. Keller, *Ecological genomics meets community-level modelling of biodiversity: mapping the genomic landscape of current and future environmental adaptation.* Ecology Letters, 2015. **18**(1): p. 1-16.
67. Fitzpatrick, M., K. Mokany, G. Manion, D. Nieto-Lugilde, and S. Ferrier. *gdm: Generalized Dissimilarity Modeling. R package version 1.5.* 2022 March 12, 2023]; Available from: https://mfitzpatrick.al.umces.edu/gdm/.
68. Revell, L.J., *phytools: an R package for phylogenetic comparative biology (and other things).* Methods in Ecology and Evolution, 2012. **3**(2): p. 217-223.
69. Yu, G., *Using ggtree to Visualize Data on Tree-Like Structures.* Current Protocols in Bioinformatics, 2020. **69**(1): p. e96.
70. Yang, E.F., R. Phookamsak, H.B. Jiang, S. Tibpromma, D.J. Bhat, S.C. Karunarathna, D.Q. Dai, J.C. Xu, and I. Promputtha, *Taxonomic Reappraisal of Periconiaceae with the Description of Three New Periconia Species from China.* J Fungi (Basel), 2022. **8**(3).
71. de la Campa, A.S., A. Garcia-Salamanca, J. Solano, J. de la Rosa, and J.L. Ramos, *Chemical and Microbiological Characterization of Atmospheric Particulate Matter during an Intense African Dust Event in Southern Spain.* Environmental Science & Technology, 2013. **47**(8): p. 3630-3638.
72. Al Salameen, F., N. Habibi, S. Uddin, K. Al Mataqi, V. Kumar, B. Al Doaij, S. Al Amad, E. Al Ali, and F. Shirshikhar, *Spatio-temporal variations in bacterial and fungal community associated with dust aerosol in Kuwait.* PLoS One, 2020. **15**(11): p. e0241283.
73. Du, P., R. Du, W. Ren, Z. Lu, Y. Zhang, and P. Fu, *Variations of bacteria and fungi in PM2.5 in Beijing, China.* Atmospheric Environment, 2018. **172**: p. 55-64.
74. Magurran, A.E., *Measuring biological diversity.* Current Biology, 2021. **31**(19): p. R1174-R1177.
75. Jiang, S., B. Sun, R. Zhu, C. Che, D. Ma, R. Wang, and H. Dai, *Airborne microbial community structure and potential pathogen identification across the PM size fractions and seasons in the urban atmosphere.* Science of The Total Environment, 2022. **831**: p. 154665.

76. Schlens, J. *A Tutorial On Principal Component Analysis: Derivation, Discussion and Singular Value Decomposition*. 2003 [cited 2023 September 2023]; Available from: https://www.cs.princeton.edu/picasso/mats/PCA-Tutorial-Intuition_jp.pdf.
77. Favet, J., A. Lapanje, A. Giongo, S. Kennedy, Y.Y. Aung, A. Cattaneo, A.G. Davis-Richardson, C.T. Brown, R. Kort, H.J. Brumsack, B. Schnetger, A. Chappell, J. Kroijenga, A. Beck, K. Schwibbert, A.H. Mohamed, T. Kirchner, P.D. de Quadros, E.W. Triplett, W.J. Broughton, and A.A. Gorbushina, *Microbial hitchhikers on intercontinental dust: Catching a lift in Chad.* The ISME Journal, 2013. **7**(4): p. 850-867.
78. Calderón-Ezquerro, M.d.C., N. Serrano-Silva, and C. Brunner-Mendoza, *Aerobiological study of bacterial and fungal community composition in the atmosphere of Mexico City throughout an annual cycle.* Environmental Pollution, 2021. **278**: p. 116858.
79. Rodriguez-Arias, R.M., J. Rojo, F. Fernandez-Gonzalez, and R. Perez-Badia, *Desert dust intrusions and their incidence on airborne biological content. Review and case study in the Iberian Peninsula.* Environmental Pollution, 2023. **316**.
80. Fuzzi, S., U. Baltensperger, K. Carslaw, S. Decesari, H. Denier van der Gon, M.C. Facchini, D. Fowler, I. Koren, B. Langford, U. Lohmann, E. Nemitz, S. Pandis, I. Riipinen, Y. Rudich, M. Schaap, J.G. Slowik, D.V. Spracklen, E. Vignati, M. Wild, M. Williams, and S. Gilardoni, *Particulate matter, air quality and climate: lessons learned and future needs.* Atmosperic Chemistry and Physics, 2015. **15**(14): p. 8217-8299.
81. Griffin, D.W., *Atmospheric movement of microorganisms in clouds of desert dust and implications for human health.* Clinical Microbiology Reviews, 2007. **20**(3): p. 459-477.
82. Gat, D., Y. Mazar, E. Cytryn, and Y. Rudich, *Origin-Dependent Variations in the Atmospheric Microbiome Community in Eastern Mediterranean Dust Storms.* Environmental Science & Technology, 2017. **51**(12): p. 6709-6718.
83. Gat, D., N. Reicher, S. Schechter, M. Alayof, M.D. Tarn, B.V. Wyld, R. Zimmermann, and Y. Rudich, *Size-Resolved Community Structure of Bacteria and Fungi Transported by Dust in the Middle East.* Frontiers in Microbiology, 2021. **12**: p. 744117.
84. Gat, D., R. Zimmermann, and Y. Rudich, *Functional Genes Profile of Atmospheric Dust in the East Mediterranean Suggests Widespread Anthropogenic Influence on Aerobiome Composition.* Journal of Geophysical Research: Biogeosciences, 2022. **127**(10): p. e2022JG007022.
85. Kellogg, C.A. and D.W. Griffin, *Aerobiology and the global transport of desert dust.* Trends in Ecology and Evolution, 2006. **21**(11): p. 638-644.
86. Prospero, J.M., E. Blades, G. Mathison, and R. Naidu, *Interhemispheric transport of viable fungi and bacteria from Africa to the Caribbean with soil dust.* Aerobiologia, 2005. **21**(1): p. 1-19.
87. Waters, S.M., S.K. Purdue, R. Armstrong, and Y. Detres, *Metagenomic investigation of African dust events in the Caribbean.* FEMS Microbiology Letters, 2020. **367**(7).
88. Schuerger, A.C., D.J. Smith, D.W. Griffin, D.A. Jaffe, B. Wawrik, S.M. Burrows, B.C. Christner, C. Gonzalez-Martin, E.K. Lipp, D.G. Schmale Iii, and H. Yu, *Science questions and knowledge gaps to study microbial transport and survival in Asian and African dust plumes reaching North America.* Aerobiologia, 2018. **34**(4): p. 425-435.
89. Stafoggia, M., S. Zauli-Sajani, J. Pey, E. Samoli, E. Alessandrini, X. Basagaña, A. Cernigliaro, M. Chiusolo, M. Demaria, J. Díaz, A. Faustini, K. Katsouyanni, A.G. Kelessis, C. Linares, S. Marchesi, S. Medina, P. Pandolfi, N. Pérez, X. Querol, G. Randi, A. Ranzi, A. Tobias, and F. Forastiere, *Desert Dust Outbreaks in Southern Europe: Contribution to Daily PM_{10} Concentrations and Short-Term Associations with Mortality and Hospital Admissions.* Environmental Health Perspectives, 2016. **124**(4): p. 413-419.

90. Querol, X., A. Tobías, N. Pérez, A. Karanasiou, F. Amato, M. Stafoggia, C. Pérez García-Pando, P. Ginoux, F. Forastiere, S. Gumy, P. Mudu, and A. Alastuey, *Monitoring the impact of desert dust outbreaks for air quality for health studies.* Environment International, 2019. **130**: p. 104867.
91. Karanasiou, A., N. Moreno, T. Moreno, M. Viana, F. de Leeuw, and X. Querol, *Health effects from Sahara dust episodes in Europe: literature review and research gaps.* Environment International, 2012. **47**: p. 107-14.
92. Fussell, J.C. and F.J. Kelly, *Mechanisms underlying the health effects of desert sand dust.* Environment International, 2021. **157**: p. 106790.
93. Heft-Neal, S., J. Burney, E. Bendavid, K.K. Voss, and M. Burke, *Dust pollution from the Sahara and African infant mortality.* Nature Sustainability, 2020. **3**(10): p. 863-871.
94. Shiraiwa, M., K. Ueda, A. Pozzer, G. Lammel, C.J. Kampf, A. Fushimi, S. Enami, A.M. Arangio, J. Frohlich-Nowoisky, Y. Fujitani, A. Furuyama, P.S.J. Lakey, J. Lelieveld, K. Lucas, Y. Morino, U. Poschl, S. Takaharna, A. Takami, H.J. Tong, B. Weber, A. Yoshino, and K. Sato, *Aerosol Health Effects from Molecular to Global Scales.* Environmental Science & Technology, 2017. **51**(23): p. 13545-13567.
95. Elmassry, M.M., N. Ray, S. Sorge, J. Webster, K. Merry, A. Caserio, D.J. Vecellio, C. Kruczek, S. Dowd, K. Ardon-Dryer, J. Vanos, and M.J. San Francisco, *Investigating the culturable atmospheric fungal and bacterial microbiome in West Texas: Implication of dust storms and origins of the air parcels.* FEMS Microbes, 2020. **1**(1): p. xtaa009.
96. Li, H., X.Y. Zhou, X.R. Yang, Y.G. Zhu, Y.W. Hong, and J.Q. Su, *Spatial and seasonal variation of the airborne microbiome in a rapidly developing city of China.* Science of the Total Environment, 2019. **665**: p. 61-68.
97. Soleimani, Z., P. Teymouri, A. Darvishi Boloorani, A. Mesdaghinia, N. Middleton, and D.W. Griffin, *An overview of bioaerosol load and health impacts associated with dust storms: A focus on the Middle East.* Atmospheric Environment, 2020. **223**: p. 117187.
98. de Longueville, F., P. Ozer, S. Doumbia, and S. Henry, *Desert dust impacts on human health: an alarming worldwide reality and a need for studies in West Africa.* International Journal of Biometeorology, 2013. **57**(1): p. 1-19.
99. Marone, A., C.T. Kane, M. Mbengue, G.S. Jenkins, D.N. Niang, M.S. Drame, and J.M. Gernand, *Characterization of Bacteria on Aerosols From Dust Events in Dakar, Senegal, West Africa.* Geohealth, 2020. **4**(6).
100. Polymenakou, P.N., M. Mandalakis, E.G. Stephanou, and A. Tselepides, *Particle size distribution of airborne microorganisms and pathogens during an Intense African dust event in the eastern Mediterranean.* Environmental Health Perspectives, 2008. **116**(3): p. 292-296.
101. Schlesinger, P., Y. Mamane, and I. Grishkan, *Transport of microorganisms to Israel during Saharan dust events.* Aerobiologia, 2006. **22**(4): p. 259-273.
102. Fröhlich-Nowoisky, J., C.J. Kampf, B. Weber, J.A. Huffman, C. Pöhlker, M.O. Andreae, N. Lang-Yona, S.M. Burrows, S.S. Gunthe, W. Elbert, H. Su, P. Hoor, E. Thines, T. Hoffmann, V.R. Després, and U. Pöschl, *Bioaerosols in the Earth system: Climate, health, and ecosystem interactions.* Atmospheric Research, 2016. **182**: p. 346-376.
103. Goudie, A.S., *Desert dust and human health disorders.* Environment International, 2014. **63**: p. 101-113.
104. Middleton, N.J., *Desert dust hazards: A global review.* Aeolian Research, 2017. **24**: p. 53-63.
105. Zhao, J., L. Jin, D. Wu, J.-w. Xie, J. Li, X.-w. Fu, Z.-y. Cong, P.-q. Fu, Y. Zhang, X.-s. Luo, X.-b. Feng, G. Zhang, J.M. Tiedje, and X.-d. Li, *Global airborne bacterial community—interactions with Earth's microbiomes and anthropogenic activities.* Proceedings of the National Academy of Sciences, 2022. **119**(42): p. e2204465119.
